# Body Size Diversity and Frequency Distributions of Neotropical Cichlid Fishes (Cichliformes: Cichlidae: Cichlinae)

**DOI:** 10.1371/journal.pone.0106336

**Published:** 2014-09-02

**Authors:** Sarah E. Steele, Hernán López-Fernández

**Affiliations:** 1 Department of Ecology and Evolutionary Biology, University of Toronto, Toronto, Ontario, Canada; 2 Department of Natural History, Royal Ontario Museum, Toronto, Ontario, Canada; Royal Ontario Museum, Canada

## Abstract

Body size is an important correlate of life history, ecology and distribution of species. Despite this, very little is known about body size evolution in fishes, particularly freshwater fishes of the Neotropics where species and body size diversity are relatively high. Phylogenetic history and body size data were used to explore body size frequency distributions in Neotropical cichlids, a broadly distributed and ecologically diverse group of fishes that is highly representative of body size diversity in Neotropical freshwater fishes. We test for divergence, phylogenetic autocorrelation and among-clade partitioning of body size space. Neotropical cichlids show low phylogenetic autocorrelation and divergence within and among taxonomic levels. Three distinct regions of body size space were identified from body size frequency distributions at various taxonomic levels corresponding to subclades of the most diverse tribe, Geophagini. These regions suggest that lineages may be evolving towards particular size optima that may be tied to specific ecological roles. The diversification of Geophagini appears to constrain the evolution of body size among other Neotropical cichlid lineages; non-Geophagini clades show lower species-richness in body size regions shared with Geophagini. Neotropical cichlid genera show less divergence and extreme body size than expected within and among tribes. Body size divergence among species may instead be present or linked to ecology at the community assembly scale.

## Introduction

The importance of body size on the life history, ecology and distribution of species has been highlighted continuously in the literature [1]–[4]. Nevertheless, little work has been completed to answer broad questions of body size evolution and its importance. In addition, few empirical studies have addressed the evolutionary processes that underlie body size distributions across geographic space and time [5]. Many studies, particularly in mammals, have addressed how body size is distributed on a broad geographic scale [6]–[8] or across the fossil record [9], but an understanding of how body size is distributed in a phylogenetic context among organisms is far from complete [5], [10]. Cope's Rule, the phyletic increase in body size over evolutionary time [11]–[12], and Bergmann's Rule, the increase of body size with increases in latitude [13], have been proposed based on the mammalian fossil record to outline fundamental patterns of body size distribution. Recent studies from other taxa have suggested that these “rules” do not always apply, and may be the exception rather than the rule [14]–[16].

Body size, like other phenotypic traits, is expected to be similar among closely related taxa due to evolutionary constraints on morphology tied to biologically and ecologically relevant characters [17], [18]. Yet there are likely many exceptions where closely related taxa are highly divergent in body size: body size divergence could allow habitat or resource partitioning in coexisting congeneric species [19], [20] or other closely related taxa, while body size shifts associated with ecomorphological differentiation could allow access to novel habitats or unused resources [21], [22]. Furthermore, if body size is so important for physiological and ecological processes, evolution towards extreme body size, especially small body size, must result in one or several evolutionary trade-offs in life-history and ecological characteristics of these species [21], [23]. But at what taxonomic resolution should we see these trade-offs occurring? How closely related would we expect species to be that share both the same body size and the same suite of behaviours, reproductive modes, diet preferences or morphologies?

Only recently has body size distribution been examined in fishes, with several studies primarily investigating the distribution of body size across geographic space [16], [24] or with regard to basic ecological characters [25]–. The evolutionary history of body size has rarely been addressed in fishes on a broad geographic scale that links possible phylogenetic constraints of body size evolution over time with the ecological and geographic distribution of extant taxa [29]–[31]. Body size reduction in fishes has been shown to be a common phenomenon in tropical systems [27], [32], particularly in freshwater environments [33], [34]. As a consequence, distributions of fishes in the tropics tend to be right-skewed [27], though direction and intensity of skew varies depending on evolutionary history, environmental characteristics and ecology [35]. Though previous work has begun to outline the link between body size and ecology in fishes, very little is known about body size distributions, evolution and the consequences of occupying a particular body size space in Neotropical fishes.

In this study we examine the distribution of body size in a phylogenetic context across Neotropical cichlids, a group of fishes with a broad geographic distribution that is also highly diverse in species and ecological roles. By examining this system we intend to outline potentially important drivers that may influence body size evolution in Neotropical fishes as a whole. Cichlids have been used as a model to study a number of biological questions in fishes due to their ecological versatility and life history traits [36]–[38]. The radiation of cichlids generated extraordinary diversity both taxonomically and ecologically within a single family. The clade of Neotropical cichlids (subfamily Cichlinae) is the third most species-rich lineage in South America following Characidae and Loricariidae [39], but a robust hypothesis of evolutionary history for these latter two groups has yet to be developed. Moreover, Cichlinae shows a high degree of ecological and body size diversity in addition to high species diversity. Despite this diversity, very little is known about Neotropical cichlid body size diversification. Sexual dimorphism does occur in several genera of Neotropical cichlids [39], [40] but it is poorly characterized, and where present, it is not known whether it is associated with sexually selected behavioural traits (e.g. sneaker males, shell-dwelling) or if it has marked impacts on ecological strategies as seen in dimorphic species of African cichlids [41]. We are not familiar with studies that have examined body size in all described Neotropical cichlids, and very few studies have examined the association between body size and ecological or life history traits that could be driving patterns we identify in this study.

Most of the species richness in Cichlinae is distributed among three tribes. Geophagini is the most species-rich (243 species [42]) and, along with Cichlasomatini (74 species [42]), is primarily restricted to South America. Heroini (176 species [42]) is the second most diverse tribe following Geophagini and has expanded from South America into Central and North America. Recent work on Neotropical cichlids has shown that the process underlying body size evolution in Cichlinae may vary among tribes, but that body size may have diverged early in the evolution of this group, which may have resulted in the accumulation of higher body size diversity over time and possibly greater divergence among distantly related lineages [43]. These analyses, however, were performed on a relatively small subset of taxa, with distributions and evolutionary patterns of body size below the tribe level not explicitly explored. Body size in Cichlinae spans a large portion of the body size space occupied by Neotropical fishes, ranging from 21 mm standard length (SL) in *Apistogramma staecki* to 990 mm total length (TL) in *Cichla temensis*. Such body size diversity in Cichlinae provides a case study to investigate how extreme body size impacts various aspects of life history, ecology and distribution of freshwater fishes. High diversity of ecology in this group presents morphological disparity and ecological diversification ideal for examining factors affecting body size evolution and a strong understanding of phylogenetic relationships within Cichlinae [44] provides the phylogenetic framework for addressing associations with body size in an evolutionary context. As a prerequisite for identifying the underlying processes and forces driving body size evolution in Cichlinae, we need a clear understanding of patterns of body size diversity at relevant phylogenetic levels and across the geographical distribution of the group. To this end, the purpose of this study is to 1) quantitatively characterize body size frequency distributions and space occupation in various clades, 2) determine if body size is randomly distributed at and among various phylogenetic levels, 3) determine if body size variation correlates with phylogenetic relatedness, and 4) to distinguish small and large-bodied taxa as a foundation for future work in body size evolution of Neotropical cichlids.

## Methods

### Data Collection and body size frequency distributions

Maximum body size available for valid fish species within the subfamily Cichlinae were taken from FishBase (see Table S1) [42]. To ensure data accuracy, body size data provided in FishBase were compared against original sources provided within the database (see Table S2). Maximum body sizes previously found from museum specimens to be larger than published data in the literature [43] were used in this study (See Table S1). Measurements were given either in standard length (SL, length from the tip of the upper lip to end of caudal peduncle), or total length (TL, length from snout to posterior edge of caudal fin). Total length data were concentrated in Heroini species. To maximize taxon sampling in our dataset we included both SL and TL data to incorporate all variation available at the genus level and representatives from all genera within Cichlinae. We tested the effect of using the two different measures and determined that exclusion of species for which only TL was available did not change the results found at the tribe or major clade (See Appendix S1, Table S3), and only affected results at the genus level (see below) in a few taxa. Exclusion only resulted in significant changes at the genus level within some heroines (12 genera, e.g. *Hoplarchus*, *Parachromis*, *Archocentrus*) in which most or all body size was given in TL. Untransformed body size data was typically skewed, and therefore log-transformed body size data were used in all analyses unless otherwise noted. Log transformation of body size data also reduces the potential bias of TL data within distributions and analyses. Unless otherwise noted, discussions of body size with regards to the results of the study and the interpretation of the figures refer to log-body size (LBS).

**Figure 1:**
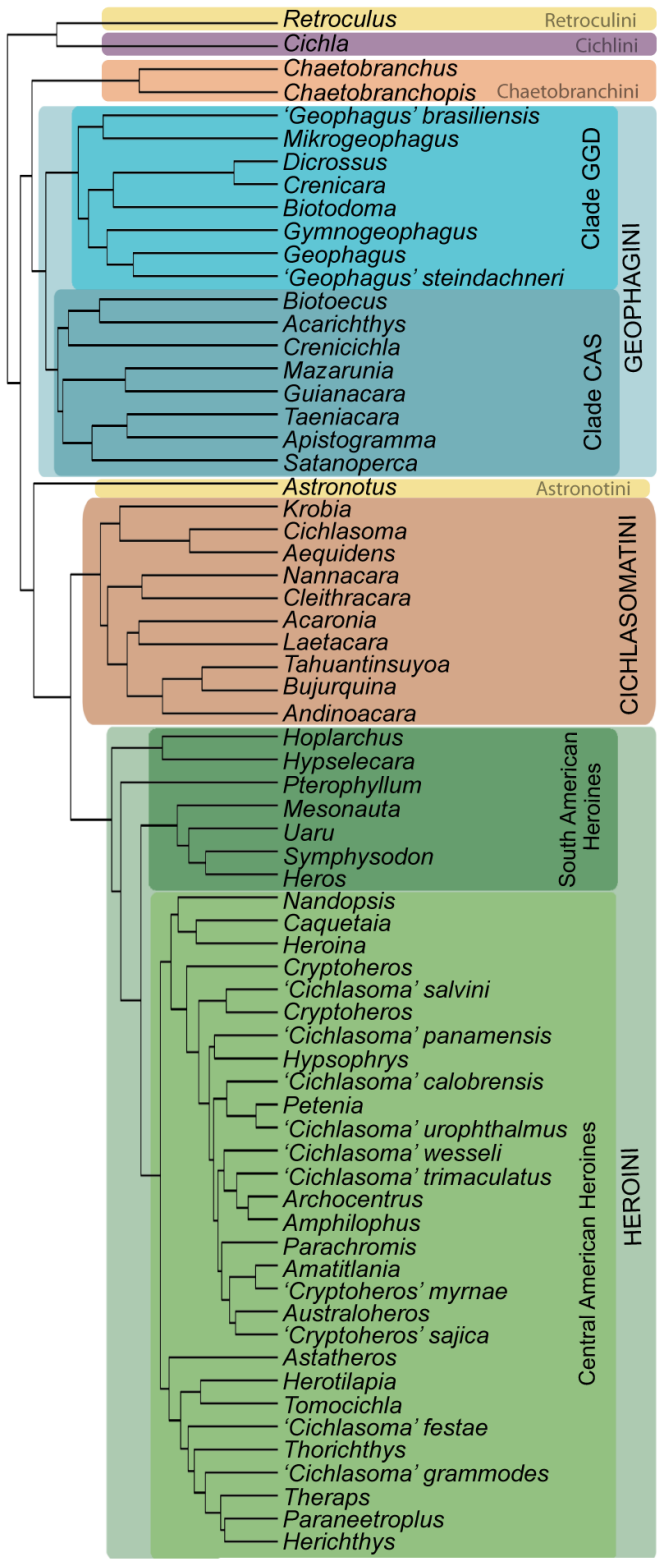
Phylogeny and taxonomic designation of Cichlinae. A chronogram of Neotropical cichlid fishes [43]. Phylogenetic nomenclature used in this paper is represented by coloured boxes for the tribes and major clades. See [44] for details on phylogenetic reconstruction and taxonomic conventions. CAS and GGD refer to the *Crenicichla-Apistogramma-Satanoperca* and *Geophagus-Gymnogeophagus-Dicrossus* clades, respectively, of Geophagini.

Species assignment and phylogenetic partitioning of the data for subclade analyses within Cichlinae followed [44]. Body size data for the subfamily Cichlinae was first partitioned by tribe. The three most diverse tribes Geophagini, Heroini and Cichlasomatini following [44] represent monophyletic clades that encompass the vast majority of taxonomic diversity and were used for further subdivision into less inclusive taxonomic units (Fig. 1), while all other tribes contain too few taxa to be partitioned further. Geophagini is composed of two major clades [44], *Crenicichla*-*Apistogramma*-*Satanoperca* (CAS) that is more species-rich and has higher morphological disparity than the *Geophagus*-*Gymnogeophagus*-*Dicrossus* (GGD) clade [45], [46]. Body size distributions of genera were analyzed separately in these two clades due to high species diversity as well as differences in ecological attributes of species. Heroini is the only tribe of Neotropical cichlids that inhabits both South and Central America, even extending into the very southern regions of North America. To test if body size frequency distributions (BSFDs) were influenced by geographic expansion, we also analyzed Heroini by separating species into South and Central American groups (hereby referred to as SA or CA heroines) (Fig. 1) Although we separate heroines geographically, it is relevant to clarify that SA heroines do not comprise a monophyletic clade. Central American heroines are monophyletic at the most basal level, but include several lineages distributed in South America that have Central American affinities [44]. The CAS clade, GGD clade, CA heroines, SA heroines and Cichlasomatini as a whole were then subdivided into the genera identified by ([44], their Fig. 1).

### Analysis of body size frequency distributions within Cichlinae

Ecologically relevant morphological traits in Neotropical cichlids are more similar within genera than among genera [45] and phenotypic divergence that resulted in currently recognized genera often followed an early-burst pattern of evolution [43], [46]. We expected to observe body size distribution patterns that were consistent with other phenotypic data which found higher similarity within clades than among-clades. Therefore we wanted to characterize body spaces occupation of clades and determine if divergence in body size is present within or among clades in Cichlinae. We analyzed the BSFDs of Neotropical cichlids with available body size data at the subfamily, tribe, major clade and genus level (Fig. 1). Analyses were only conducted on monophyletic groups with two or more taxa, with the exception of the SA Heroini (see above). The mean, standard deviation, range, 25% and 75% quantiles and interquartile range (IQR) were calculated for each BSFD. Significant deviations in mean body size may indicate shifts in body space occupation among clades. Significantly lowered standard deviation, range and interquartile range would indicate a lowering of body size diversity within clades, suggesting constrained size, while increases would indicate expansions in body size diversity. Location of 25% and 75% quantiles together also indicate information about body size diversity, proportion of taxa within certain body size ranges and skew, but interpretation is not as clear. In addition, distributions were tested for unimodality using Hartigan's dip test [47] and characterized by kurtosis and skew. Platykurtosis, flatter as compared to a normal distribution, and leptokurtosis, more peaked than a normal distribution, give indications of constraints around the mean body size. Right-skew indicates a higher proportion of small-bodied species while left-skew indicates a higher proportion of large-bodied species within a distribution.

To determine if BSFDs were different among phylogenetic levels, distributions were first compared using the Kolmogorov-Smirnov (K-S) two-sample test. This test identifies differences between two observed frequency distributions and is particularly sensitive to deviations in skew, kurtosis and location along the body size gradient. We employed a Bonferroni correction to account for multiple comparisons among genera in the K-S analysis (P_adj_<0.00004). The K-S only determines whether two distributions differ, but does not identify what aspects of the distribution drive those differences.

We employed a bootstrapping method to test for random distribution of BSFDs between phylogenetic levels [5], [48]. The BSFD of the higher taxonomic unit containing the focal clade was resampled to create 1000 randomly assembled BSFDs equal in size to the focal clade (see above). The mean, standard deviation, range, 25% and 75% quantiles, interquartile range, kurtosis and skew were then calculated for each of the1000 BSFDs. A distribution of each summary statistics expected under a random phylogenetic distribution was then obtained. We assumed that the summary statistics of the observed data could either be higher or lower than the simulated data, so a two-tailed adjusted alpha level of 0.05 was applied. Summary statistics for observed body size distributions found below and above the 2.5% and 97.5% quantiles of the simulated summary statistic distributions were considered to significantly deviate from summary statistics describing randomly distributed simulated data. A p-value was not directly calculated for each bootstrap simulation, but all deviations under or over the above thresholds were reported as significant (p<0.05). We also compared observed data to the 0.25% and 99.75% quantiles (P<0.005) (see Table S4). The BSFDs of subclades were compared to bootstrap pseudo-distributions created from respective clades in each successive phylogenetic level. If clades showed few or no deviations from pseudo-distributions, body size was considered a random subset of the containing higher taxonomic level, suggesting low phylogenetic autocorrelation. Clades that show a number of significantly different summary statistics have a specialized or partitioned body size space occupation as compared to distributions at higher taxonomic levels. We also tested for phylogenetic autocorrelation in body size using Moran's *I* [5], [10] to determine if body size was randomly distributed within a given taxonomic level. Values of Moran's *I* fall between -1 and 1, with higher values indicating the trait is more similar within taxonomic units than expected at random, 0 indicating random distribution, and values approaching -1 indicating the trait is more different than random.

To account for potential taxonomic error, all genera were compared to the higher clade and tribe that contained them (Fig. 1). The distributions of major clades were then compared to their respective tribe. Finally the BSFDs of each tribe were compared to the BSFD of Cichlinae. The genera *Crenicichla* and *Teleocichla* of Geophagini are known to form a monophyletic clade with *Teleocichla* potentially interspersed among *Crenicichla* species [44], [49], therefore body size of all species in both genera were analyzed together. Heroini contains a paraphyletic, catch-all genus ‘*Cichlasoma*’ which was not analyzed at the genus level [50], [51]. Species of *‘Cichlasoma’* with body size data available were included in the Heroini and CA Heroini BSFDs to be resampled with phylogenetic assignment following [44].

## Results

### Characterization of cichlid body size frequency distributions

Based on the data available from FishBase [42], we were able to include 498 cichlid species in our analyses of BSFDs (Table S1). This represents approximately 88% of the valid Neotropical cichlid species listed on FishBase at the time of the study. The bootstrap analyses found that only nine genera across the three main tribes had significantly smaller means than expected if body size was randomly distributed throughout the phylogeny, while eight genera had higher than expected mean body size (Table S4). These findings were generally consistent when comparing distributions of genera to respective major clades as well as at the tribe level (Table S4). Occurrences of mean body size deviation were not more frequent in any particular tribe (Geophagini 6/16; Heroini 8/25; Cichlasomatini 3/10) or major clade and no tribe was biased towards smaller or larger body size (Table S4). Standard deviation was typically lower in all significant results, and lowering of body size diversity was particularly apparent in the CAS clade of Geophagini (5/9 cases; GGD 2/7; SA heroines 2/7; CA heroines 6/18; Cichlasomatini 1/10) (Table S4). Deviations in skew and kurtosis were not typically found at any phylogenetic level (Table 1).

**Table 1:** Quantifying cichlid body size and testing for divergence.

Taxa		*N*	Mean	St Dev	Minimum	Maximum	25% Quantile	75% Quantile	Kurtosis	Skew	IQR
Cichlinae (Subfamily)		498	2.08	0.29	1.36	3.00	1.89	2.28	−0.27	−0.01	0.39
Geophagini (Tribe)*		226	1.97**	0.31**	1.32**	2.49**	1.69**	2.23*	−1.25*	−0.15	0.54**
Clade CAS		180	1.94*	0.33**	1.32	2.49	1.62**	2.34**	−1.38**	0.01**	0.61**
	Apistogramma*	67	1.60**	0.13**	1.32	2.14**	1.53**	1.69**	2.93**	0.87**	0.17**
	Crenicichla*	91	2.17**	0.22**	1.60**	2.49	2.05**	2.35**	−0.53**	−0.62**	0.29**
	*Satanoperca*	8	2.25*	0.08**	2.15**	2.41	2.21**	2.28	−0.64	0.65	0.07**
Clade GGD*		46	2.07*	0.21**	1.53**	2.44	1.99**	2.23	−0.10**	−0.62**	0.25**
	*Gymnogeophagus*	11	2.06	0.09**	1.93	2.19*	2.00	2.12	−1.51	−0.01	0.13
	Dicrossus*	5	1.71**	0.11	1.58	1.85**	1.62	1.78**	−1.99	0.01	0.15
	*Geophagus*	23	2.19**	0.13**	1.88**	2.38	2.10**	2.30*	−0.79	−0.53	0.20
	*Mikrogeophagus*	2	1.63*	0.15	1.53	1.75*	1.59*	1.69*	−2.75	0.00	0.11
Heroini (Tribe)*		176	2.18**	0.21**	1.70**	2.70	2.01**	2.35*	−0.57	−0.014	0.34*
SA Heroines		21	2.09*	0.18	1.70	2.40	1.97	2.20	−0.81	−0.22	0.23
CA Heroines		153	2.19*	0.21	1.72	2.70	2.04	2.38*	−0.63	−0.01	0.34
Cichlasomatini (Tribe)*		72	1.98**	0.15**	1.56	2.30**	1.87	2.08**	−0.20	−0.47	0.21**
	*Laetacara*	6	1.77**	0.14	1.56	1.91**	1.69*	1.86**	−1.66	−0.44	0.17
	Nannacara*	5	1.72**	0.07	1.65	1.83**	1.69*	1.75**	−1.61	0.48	0.06

Summary statistics for Cichlinae, tribes, major clades and genera with Body Size Frequency Distributions (BSFDs) deviating from that of their containing clades (log_10_ transformed length in mm). An asterisk next to subclade names indicates a significant difference from the more inclusive clade above (Kolmogorov-Smirnov Test). An asterisk next to statistical values indicates significantly different values as compared to a random phylogenetic distribution attained by bootstrap simulations (* indicates p<0.05; ** indicates p<0.005). Direction of deviation can be found by comparing the value of summary statistics between clades and respective subclades. *Crenicichla-Apistogramma-Satanoperca* (CAS); *Geophagus-Gymnogeophagus-Dicrossus* (GGD); Number of species (N); Standard Deviation (St Dev); Interquartile Range (IQR).

**Figure 2:**
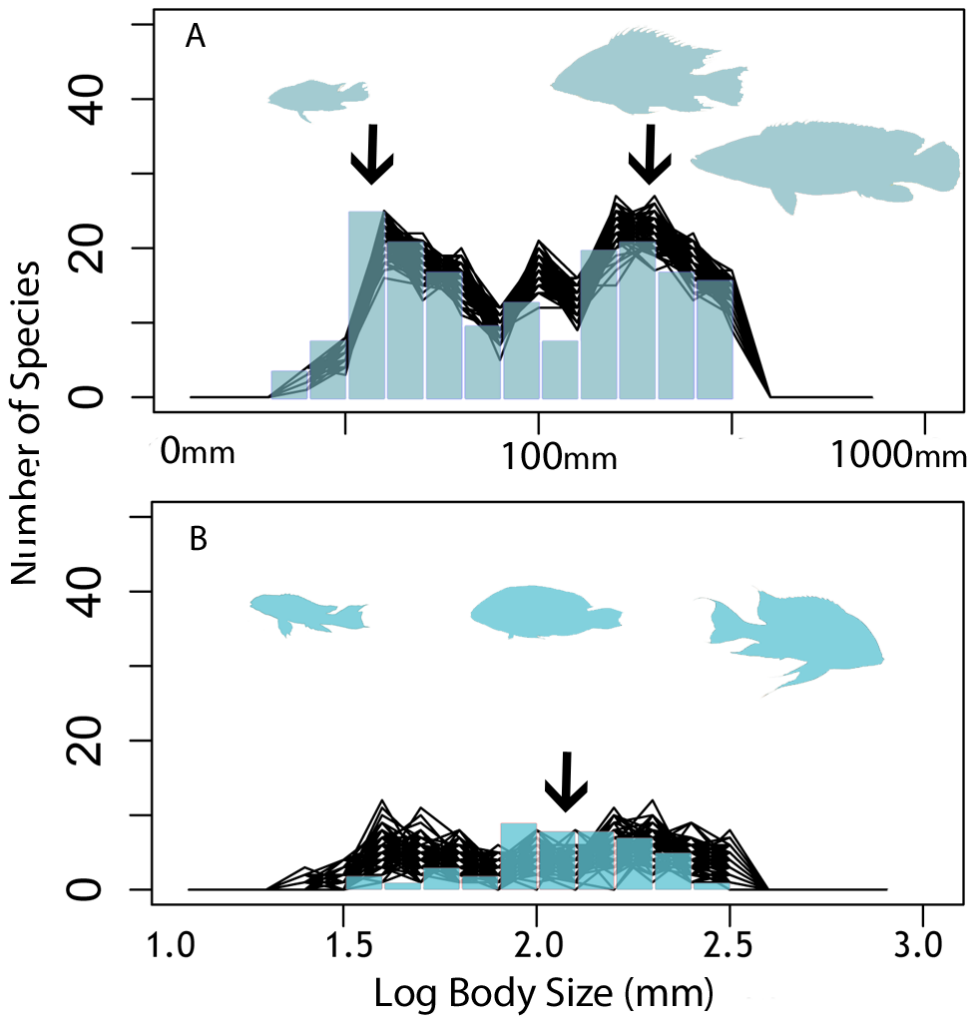
Body size occupation in subclades of Geophagini. Body size frequency distributions of the *Crenicichla-Apistogramma-Satanoperca* (CAS) Clade (A) and the *Geophagus-Gymnogeophagus-Dicrossus* (GGD) Clade (B) of Geophagini. Coloured columns show observed data fitted to 1000 random subsamples of Geophagini each represented by a black line. Silhouettes depict the three most diverse genera for each clade and the primary region of body space occupation, from top-left to bottom right: *Apistogramma*, *Satanoperca*, *Crenicichla*, *Dicrossus*, *Gymnogeophagus*, *Geophagus*. Black arrows indicate potential body size optima supported by the bootstrap analyses.

**Figure 3:**
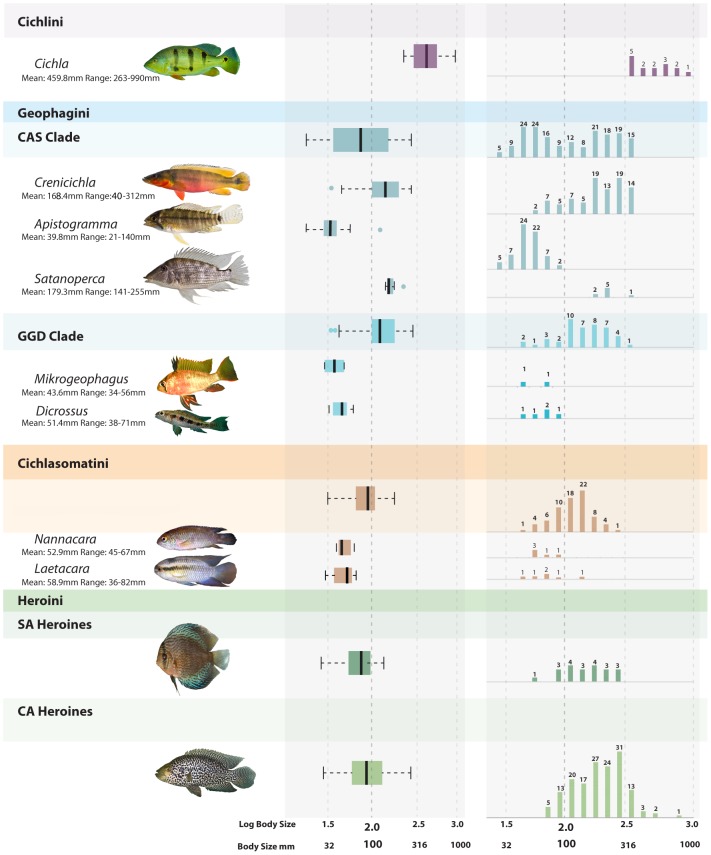
Body size diversity and occupation of Cichlinae. Distributions of body size within Cichlinae and the major subclades of Geophagini and Heroini. Body size distributions of genera with BSFDs deviating from their containing clades are included. Representatives of each clade are given; photographs not to scale. Dots to the left and right of boxplots indicate outliers of the distribution. Numbers above bars indicate the number of species within that particular body size bin. CAS and GGD refer to the *Crenicichla-Apistogramma-Satanoperca* and *Geophagus-Gymnogeophagus-Dicrossus* clades, respectively, of Geophagini. Photographs by Hernán López-Fernández and courtesy of Anton Lamboj. Body size threshold proposed [46] identified by prominent dashed line and bold font on axis. See Figure S1 for additional distributions.

The mean of the CAS BSFD was significantly lower than expected while the standard deviation and IQR were higher (Table 1). The maximum and minimum body sizes are not significantly different than expected. Despite this, the 25% and 75% quantiles were closer to the extremes of the distribution than expected (Table 1; Fig. 2A). The distribution was also significantly more platykurtic and with a higher right-skew than Geophagini. The BSFD of CAS is also strongly bimodal (p = 0.0026) (Fig. 2A), with the small-bodied peak (LBS 1.54, 35 mm) coinciding with the distribution of *Apistogramma* (Fig. 3), and the large-bodied peak (LBS 2.40, 250 mm) coinciding with the peak of *Crenicichla* (Fig. 3). Despite the bimodality of CAS, none of its subclades deviate from a unimodal distribution. The mean of GGD was significantly higher than expected at LBS 2.07(118.2 mm) while standard deviation and IQR were lower than expected (Table 1; Fig. 2B). Minimum body size and 25% quantile were higher than expected. The BSFD of GGD did not significantly deviate from unimodality, however it was more platykurtic and left-skewed than expected. SA heroines had a significantly lower mean than expected, and a slight trend towards lower standard deviation than expected (Table 1). CA heroines did not deviate from the BSFD of Heroini in any summary statistic, except for a higher mean and 75% quantile than expected (Table 1).

**Figure 4:**
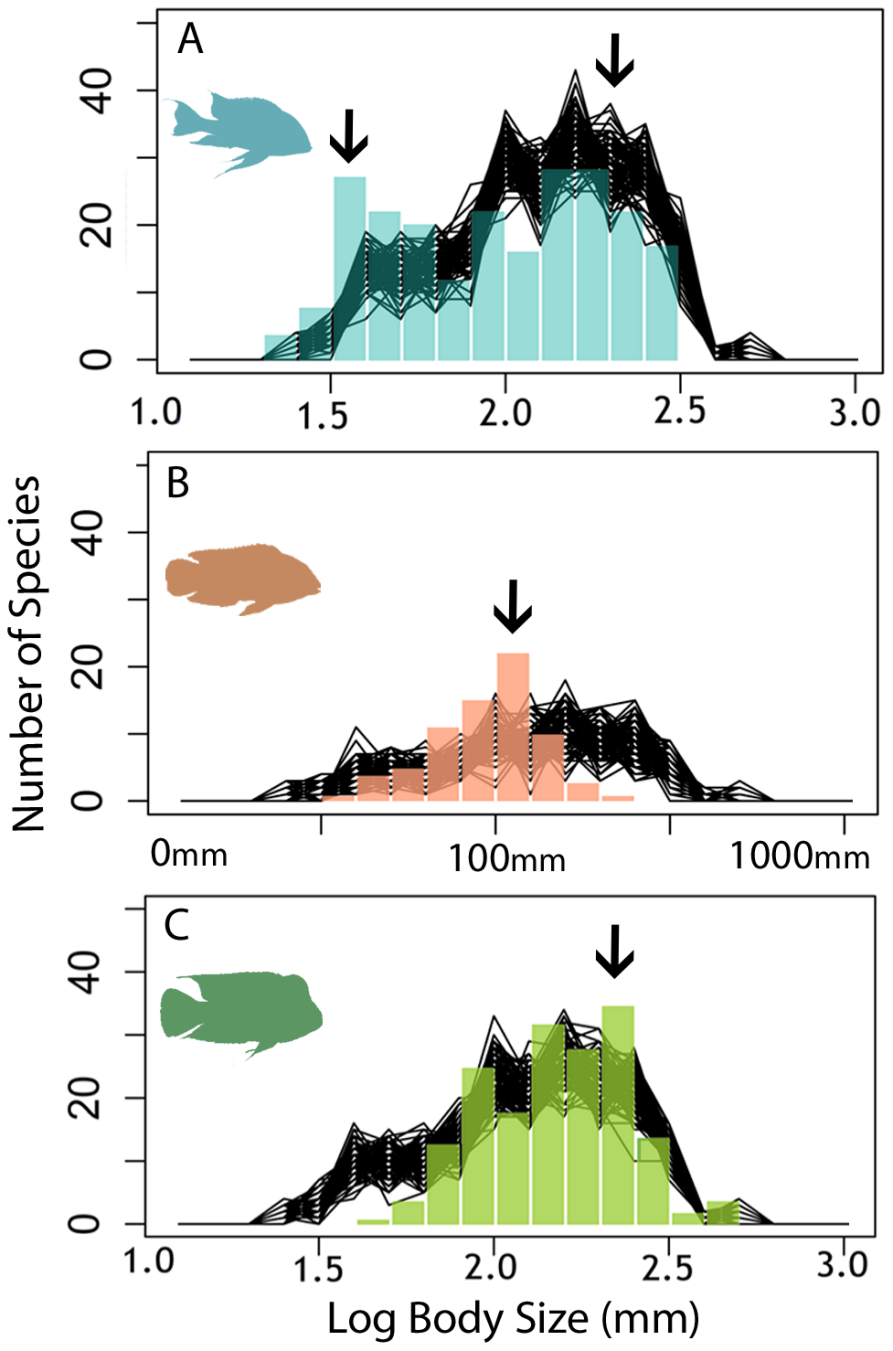
Body size occupation of major cichlid tribes. Body size frequency distributions for major tribes of Cichlinae: A) Geophagini, B) Cichlasomatini, C) Heroini. The coloured columns show observed data, black lines represent the distributions of 1000 random bootstrap subsamples of Cichlinae. Black arrows indicate potential body size optima supported by the bootstrap analyses.

The BSFD of Cichlinae was unimodal, slightly left-skewed with a mean of LBS 2.08 (120.2 mm), and IQR from LBS 1.89 (77.6 mm) to 2.28 (190.5 mm). Bootstrap analyses revealed Geophagini had a significantly lower mean of LBS 1.97 (93.3 mm), accompanied by significantly lower minimum body size, maximum body size, 25% quantile and 75% quantile (Table 1). Geophagini tended towards a bimodal distribution (p = 0.0642) (Fig. 4A), which is also reflected by a significantly higher standard deviation and IQR as well as being significantly platykurtic. The mean of Heroini BSFD was significantly higher, at LBS 2.18 (151.0 mm), than expected (Table 1). Heroini shows significantly lower standard deviation and IQR, suggesting a restricted range of body size. This is supported by a significantly larger minimum body size and 25% quantile, however the 75% quantile is higher than expected. The mean of Cichlasomatini of LBS 1.98 (94.6 mm) and standard deviation were significantly lower than expected (Table 1). Maximum body size, 75% quantile and IQR were also significantly lower than expected while the minimum body size was higher.

### Distribution of body size among taxonomic levels

If body size is randomly distributed across a phylogeny (i.e. no phylogenetic autocorrelation), we would expect little deviation of BSFDs between clades and their subclades, as well as high similarity between distributions of related subclades at the same taxonomic level [5], [10]. High correlation within clades (phylogenetic autocorrelation) should result in considerable partitioning of body size space. Only 46 out of 1249 pairwise comparisons between Cichlinae genera showed significantly different BSFDs from each other (p_adj_<0.00004) using the K-S test (for comparisons of major clades see Table 2; results at genus level not shown), supporting randomly distributed body size across genera. Of these 46 cases, 14 comparisons involved *Cichla* (Cichlini), a large-bodied piscivorous genus occupying a body size space that few other taxa occupy. In addition, 17 other cases involved the “dwarf” cichlid genus *Apistogramma* (Geophagini), which significantly differed from several genera across all three tribes. The remaining 15 cases typically involved comparisons between genera from different tribe affinities rather than divergence of genera within the same tribe. Analysis of phylogenetic autocorrelation in Cichlinae showed strong body size correlation with phylogenetic history at the genus level (I = 0.7010, p<<0.05), however at more inclusive taxonomic levels (subclade, tribe) body size was not correlated with phylogenetic history. Species within a genus are more similar in body size to each other than expected at random, however at higher taxonomic levels body size may or may not be similar in closely related groups.

**Table 2:** Partitioning of body size within Cichlinae and major clades.

Taxa	Cichlinae		Geophagini		Clade GGD		Heroini		SA Heroines		CA Heroines	
	*D*	*P*	*D*	*P*	*D*	*P*	*D*	*P*	*D*	*P*	*D*	*P*
Geophagini	0.1586	**<0.001**	-	-	-	-	-	-	-	-	-	-
Clade CAS	-	-	0.0686	0.7327	0.3372	**0.0005**	-	-	-	-	-	-
Clade GGD	-	-	0.2686	**0.0081**	-	-	-	-	-	-	-	-
Heroini	0.1885	**<0.0005**	0.342	**<0.0001**	-	-	-	-	0.2108	0.3751	0.0053	1
Cichlasomatini	0.3147	**<0.0001**	0.2871	**<0.0005**	-	-	0.5032	**<0.0001**	-	-	-	-
SA Heroines	-	-	-	-	-	-	-	-	-	-	0.2133	0.3608

D statistic and corresponding significance values of Kolmogorov-Smirnov tests for differences in body size frequency distributions between and among phylogenetic levels. Results shown are for analyses at the tribe and major clade level. P-values in bold indicate significantly different comparisons, dashes indicate pairwise comparisons that were not made between clades of differing tribes (see text).

The BSFDs of all three major tribes were found to be significantly different from that of Cichlinae and from each other using the K-S test (Table 2). The CAS clade was not significantly different from the BSDF of Geophagini while the GGD clade was found to be significantly different from both the BSFD of Geophagini and the CAS clade (Table 2). SA heroines and CA heroines did not significantly differ from the BSFD of Heroini or each other based on the K-S test.

### Divergence in body size space

In addition to looking at phylogenetic autocorrelation of body size among taxonomic levels, we also wanted to explore body size space occupied by closely related taxa. We compared the location of distributions in body size space as well as bootstrap analysis results of divergent taxa to determine if deviations in summary statistics supported different body space occupation. At the tribe and major clade level there is considerable overlap in body size space, with no groups showing complete separation of body size space occupation.

Within Geophagini there are several cases of divergence in body size space within closely related genera. Within the CAS clade, the distributions of *Apistogramma* and *Satanoperca* do not show overlap in body size space (Fig. 3). *Biotoecus* and *Acarichthys* (See Fig. S1), proposed sister-groups, also do not show overlap in body size space, though no bootstrap results support divergence greater than expected at random (Table S4). The sister-groups *Guianacara* and *Mazarunia* occupy a narrow range of body size space around 100 mm (LBS 2.0) and do not show significant divergence from each other (Fig. S1). The small-bodied space dominated by *Apistogramma* is primarily shared with *Teleocichla* and some small-bodied *Crenicichla*, while large body size space is equally shared between *Satanoperca* and *Crenicichla* (Fig.3). In the GGD clade, the sister-groups *Dicrossus* and *Crenicara* show significant divergence based on bootstrap results (Table S4) and non-overlapping distributions in body size space (Fig. S1). While the sister-groups *Geophagus* and *Gymnogeophagus* show considerable overlap in body size space (Fig. S1), bootstrap results suggest that the range of body size in *Gymnogeophagus* is significantly reduced and overlaps only with the lower end of the *Geophagus* distribution (Table S4). In addition, the distribution of *Geophagus* occupies a narrower body size space, shifted towards large body sizes. Smaller bodied taxa (*Mikrogeophagus*, *Dicrossus*, *Crenicara*; Fig. 3) show little or no overlap with the distributions of *Geophagus* and *Gymnogeophagus* while *Biotodoma* occupies a narrow range of body size space around 100 mm (LBS 2.0) (Fig. S1).

Body size distributions of genera within Cichlasomatini commonly overlap in closely related genera. Two exceptions occur between sister-groups *Nannacara* and *Cleithracara* as well as *Acaronia* and *Laetacara* (Fig. S1). In both cases, no overlap is seen between taxa although these divergences are not supported strongly by the bootstrapping analyses (Table S4) due to the low species richness of these genera. In CA heroines, divergence was difficult to assess due to the paraphyletic and unresolved nature of many groups. However, two cases of divergence occur in South America between *Uaru* and its sister clade containing *Symphysodon* and *Heros* as well as between *Hoplarchus* and *Hypselecara* (Fig. S1). No overlap is seen between taxa, but again these divergences are not supported by the bootstrapping analyses likely due to low species richness (Table S4).

## Discussion

### Divergence in body size space

Morphological traits associated with diet in Neotropical cichlids are known to be more similar within genera than among genera [45]. Since body size is often strongly linked to diet and feeding [52] and morphology [53] in fishes, we hypothesized body size would also show this pattern of higher similarity within genera than among genera. A reduction of body size range and variation as compared to a random phylogenetic distribution was expected if species within a genus share more similar body sizes. However, this pattern was only consistent within the CAS clade of Geophagini, perhaps due to the specialized ecological roles within this clade compared to others [43], [46] that is associated with particular body size regions (Table 1; Fig. 3). In addition, we expected a low degree of body size overlap among clades, consistent with ecological divergence and niche partitioning hypotheses [20], [52]. Body size distributions of genera within and among major clades or tribes did not show a high amount of body size divergence. This was supported by results of the K-S test, bootstrap simulations and analysis of phylogenetic autocorrelation. Most differences among genera occur with *Apistogramma* (Geophagini) or *Cichla* (Cichlini) (Fig. 3), which occupy extreme areas of small and large body size space, respectively, and which are rarely occupied by other genera. Body size can be an important determinant of niche partitioning or overlap [54] as well as of trophic level and resource use [52], but among Cichlinae, it is not highly divergent in a strict phylogenetic context, and may in fact be more important within the context of community ecology and assembly [28], [54]. Body size divergence in distantly related taxa may allow partitioning of resources within a community by differing in resource use (e.g. microhabitat, prey type and size), while divergent ecologies in similarly sized cichlids may also allow for coexistence. Analyses at the community level will be needed to understand the role of body size in the ecological and geographic assembly of Neotropical cichlid fishes.

The distributions of SA and CA heroines were also not highly divergent (Fig. 3), but there was a trend towards lower diversity in SA heroines and smaller body size. Heroine species diversity is higher in Central America, which may be expected to increase body size diversity. However, mean body size of CA heroines is higher than expected, suggesting that despite having more species, evolution in CA heroines may be biased towards body size increase. This trend may, in part, be influenced by the higher success of large-bodied species at dispersal [26], [27], [55], [56], and therefore the founders of the Central American colonization may have been primarily larger-bodied cichlids. The recent finding that Heroini body size may be evolving under an adaptive-peak model of evolution [43] suggests that novel environmental pressures or opportunities in Central America could have also acted on dispersing cichlids to drive increases in CA Heroine body size [57], [58]. Restricted body size occupation in SA heroines could be associated with ecological constraint as compared to CA heroines, particularly as a result of interaction with the much more diverse Geophagini [43]. The diversification of ecological roles, particularly the evolution of piscivorous heroines in Central America, could be related to the origin of larger body size in middle American heroines [43], [52].

### Body size diversity and the evolution of extreme body sizes

Body size diversity appears to be higher in Geophagini (Fig. 4), which has a higher standard deviation and IQR than expected (Table 1). However, Heroini spans the largest range of body size space while Cichlasomatini has the narrowest range. Despite having significantly larger body size, less than 4% of heroines have increased in body size beyond the largest Geophagine cichlids, and still do not occupy a unique body size space (Fig. 4). This extremely large body size space is also occupied by *Cichla* and *Astronotus*, large predatory cichlids from two different species-poor tribes (Cichlini and Astronotini, respectively). In contrast, heroine cichlids have not expanded into small bodied space to the extent that Geophagini has (Fig. 4). In Geophagini, approximately 10% of species occupy a region of body size unoccupied by any other tribe while 25% of species occupy a region of body size unoccupied by heroine cichlids. The genus *Apistogramma* (Fig. 3) not only dominates in the extent of body size reduction within Cichlinae as a whole (minimum BS 21 mm SL, LBS 1.32), but also in the total number of species that occupy this unique space (22 spp below 36 mm SL, LBS 1.57; 53 spp below 50 mm SL, LBS 1.70). Comparatively, only ten species from other groups also occupy this space.

The restriction of extremely small-bodied species to particular genera and conservation of body size within these groups suggests that morphological evolution may be constrained at smaller size, which could limit ecological opportunity within small-bodied taxa [52], [59]. Though variance in body size was not correlated with body size (results not shown), in that large-bodied clades do not have higher body size diversity relative to small-bodied clades, evidence for ecomorphological constraint within these small-bodied taxa has been found in Geophagini. Small-bodied genera within Geophagini were found to converge on the same trophic functional morphospace below a body size threshold of 100 mm SL [46] (LBS 2.0, identified in Fig. 3 by a prominent dashed line for comparison). This threshold appears to be supported by the BSFD of the CAS clade, but our results also suggest that at least three regions of body size space may better characterize body space occupation, each with possible size-specific ecological roles in Neotropical cichlids.

Cichlinae is significantly left-skewed compared to other Neotropical fishes, with a higher proportion of large-bodied species. This finding is inconsistent with typical patterns found in tropical riverine fishes [27] where increasingly right-skewed distributions occur in lower latitudes. Below the subfamily level, clades did not typically deviate in skew and therefore extreme body size reduction is rare in Cichlinae. Subsequently, Geophagini offers a unique case to study the ecologies of small-bodied fishes prevalent in the Neotropics and consequences of extreme body size reduction. The most species-rich genus of Neotropical cichlids, *Crenicichla* (including *Teleocichla*) has the largest range of body size, from 39.8 mm SL (LBS 1.60) to 312 mm SL (LBS 2.49). Though this range only occupies one third of the body size range of all Cichlinae and does not reach the lower and upper extremes, this range does encompass 75% of species within the BSFD of Cichlinae (Fig. 3). *Crenicichla* and *Teleocichla*, though not as ecologically diverse as Geophagini, could offer a more practical group to study narrow questions of body size evolution in a strict phylogenetic, ecological and geographic context.

### Is body size adaptive?

Recent studies of BSFDs in North American freshwater fishes found that bimodality in distributions was typically influenced by the presence of small-bodied, resident, habitat specialists and large-bodied, migratory, habitat generalists [27]. Recently, strong partitioning of ecologically meaningful body shape attributes [43] and trophic functional morphospace [46] was found in Cichlinae and it is likely that this ecological differentiation is correlated with patterns in BSFDs across the group. The distribution of body size in the GGD clade of Geophagini was found to be located between the two modes of the CAS clade, producing a third mode of body size that suggests partitioning of total body size morphospace. The CAS clade began diversifying before all other species-rich clades within Cichlinae [43] and may have constrained body size diversification within other lineages. In addition, the distributions of GGD and Cichlasomatini are also located between the two modes of the CAS clade and show narrowing of body size space occupation (Fig. 3). SA heroines occupy a similar body size space to the large-bodied mode of the CAS clade, but have considerably less species diversity. This pattern of roughly complementary body size distribution among clades may further support the hypothesis that competition and ecological opportunity may be important in the diversification of traits, including body size, in South American cichlids. Analyses of ecomorphology [43] and biomechanics [46] have shown a high degree of diversity in Geophagini as compared to South American Heroini and Cichlasomatini, a pattern consistent with our findings in this paper. Interestingly, the BSFD of CA heroines, which are geographically separated from other Neotropical cichlids, occupies the same body size space as the large-bodied CAS Geophagini and show comparable species richness (Fig. 3). This result is also consistent with patterns found in body shape disparity [43], with heroines occupying non-overlapping morphospace with Geophagini in South America, then expanding into these newly available ecomorphospace regions once geographically separated (Fig. 3).

The pattern of BSFDs complementarity at the major clade and tribe levels support a hypothesis of three possible body size optima in Cichlinae: a “miniature” body size optimum around 35 mm (SL), a “ mid-sized” optimum around 100 mm (SL) and a “large-bodied” optimum around 250 mm (SL) (Fig. 2; Fig. 4). The CAS and GGD clades of Geophagini show modes around these three optima that are quantitatively supported as distinct from each other by the K-S tests and bootstrap analyses (Fig. 2, Tables 1 and 2), while the body sizes of Cichlasomatini and South American heroines form a unimodal distribution around the 100 mm optimum (Figs. 3; Fig. 4). Finally the CA heroines have a significantly right shifted but unimodal distribution around the large-bodied optimum of 250 mm SL (Fig. 3). These results are inconsistent with the idea of a phylogenetically directional pattern of evolution towards large body size, as proposed by Cope's rule. Decreases in body size are a common phenomenon in Neotropical cichlids, and are present in both old and young clades, although the phylogenetic directionality of body size changes and number of independent reductions still need to be tested directly. Likewise, testing for body size optima was beyond the scope of this paper as it should be done using phylogenies with complete or near-complete species-level sampling. Instead, until more detailed phylogenies become available, we employed less phylogenetically robust analyses incorporating all species with available body size data to determine patterns of body size within phylogenetic groups while accounting for as much body size variation in each group as possible. We were also unable to directly test the association between body size, morphology and ecology for all Neotropical cichlids since data is largely unavailable for most species used in this study.

This paper outlines a number of broad patterns in body size within a widely distributed group that is both taxonomically and ecologically diverse. Body size distributions of related clades show an unexpected amount of overlap, and an understanding of how body size is associated with geographic separation, community structure and ecological divergence may shed light on why Neotropical cichlids so frequently occupy overlapping body size space. Cichlinae represents the most studied Neotropical freshwater fish lineage to date. With a strong knowledge of the evolutionary processes driving ecological diversification in the context of a robust understanding of the phylogenetic history of the group, we can derive and test hypotheses of diversification in an explicit macroevolutionary context. Such an approach should reveal how body size evolves in Neotropical freshwater fishes and the ecological consequences or opportunities associated with body size space occupation.

## Supporting Information

Figure S1 **Body size diversity and occupation of Cichlinae.** Distributions of body size of genera within Cichlinae following approximate phylogenetic order (See Figure 1, main text). Dots to the left and right of boxplots indicate outliers of the distribution.
(TIF)

Table S1 **Body size data of Neotropical cichlids.** Cichlid species and taxonomic affiliation used within this study.
(DOCX)

Table S2 **Data verification and description of data inconsistencies.** Discrepancies between the maximum sizes on FishBase and maximum sizes in the original literature.
(DOCX)

Table S3 **Testing for effects of TL data in quantifying cichlid body size.** Changes to the summary statistics and bootstrapping results when species represented by total length (TL) data were removed from the analyses.
(DOCX)

Table S4 **Summary statistics of cichlid body size distributions and expectation under random phylogenetic distribution.**
(DOCX)

Appendix S1 **Effects of total length data on body size distributions.** Description of the effects of the inclusion and exclusion of total length data on summary statistics.
(DOCX)
