# Supplementary figures and images for: Body Size Diversity and Frequency Distributions of Neotropical Cichlid Fishes (Cichliformes: Cichlidae: Cichlinae)
Source: PLoS One. 2014 Sep 2;9(9):e106336. doi: 10.1371/journal.pone.0106336 (PMC4152270; doi:10.1371/journal.pone.0106336)

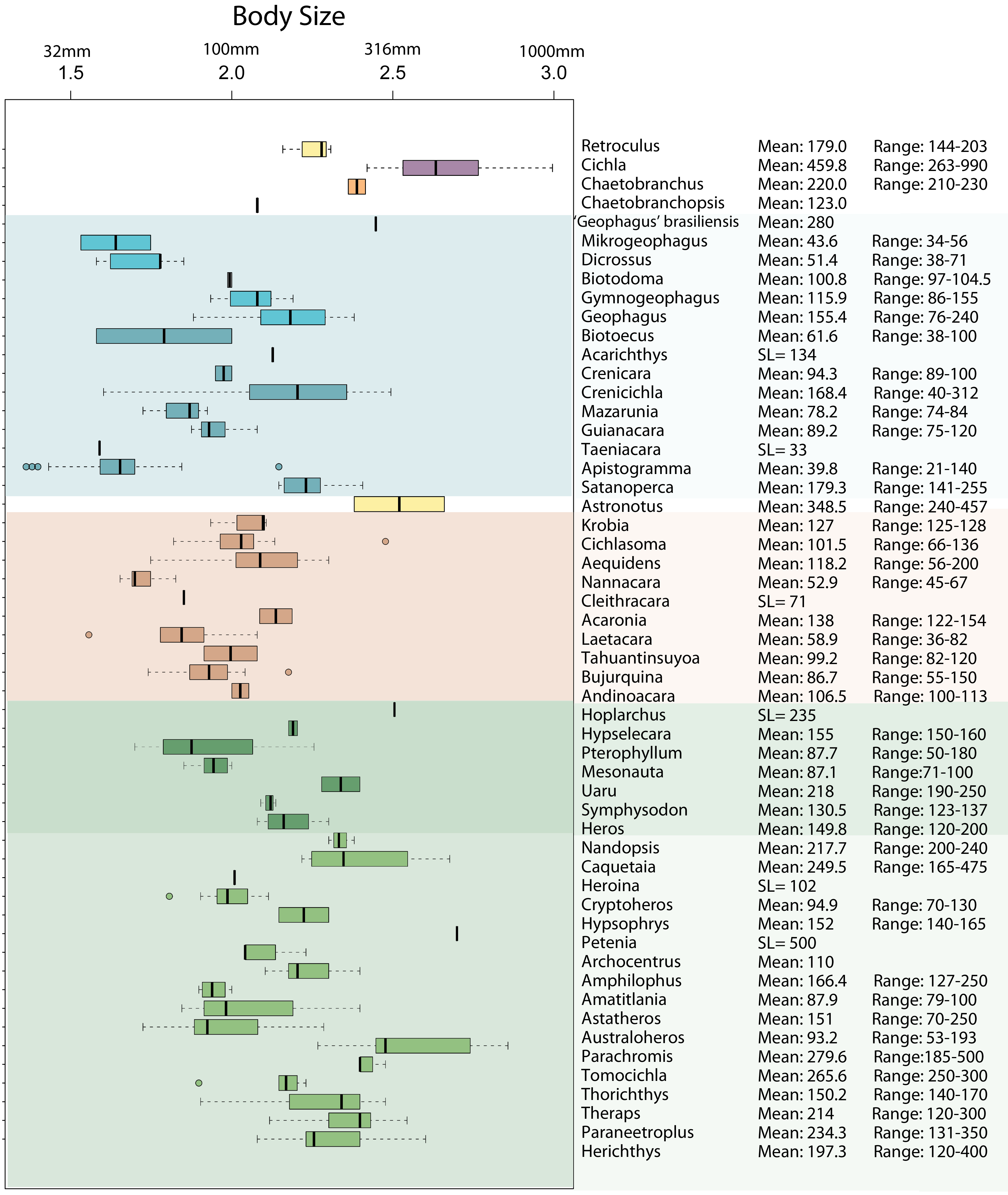

Supplement: Figure S1 — Body size diversity and occupation of Cichlinae. Distributions of body size of genera within Cichlinae following approximate phylogenetic order (See Figure 1, main text). Dots to the left and right of boxplots indicate outliers of the distribution. (TIF) [file pone.0106336.s001.tif]
