# Supplementary material for: Body Size Diversity and Frequency Distributions of Neotropical Cichlid Fishes (Cichliformes: Cichlidae: Cichlinae)
Source: PLoS One. 2014 Sep 2;9(9):e106336. doi: 10.1371/journal.pone.0106336 (PMC4152270; doi:10.1371/journal.pone.0106336)
Supplement: Table S1 — Body size data of Neotropical cichlids. Cichlid species and taxonomic affiliation used within this study. (DOCX) [file pone.0106336.s002.docx]

**Table S1:** **Body size data of Neotropical cichlids.** Cichlid species and taxonomic affiliation used within this study. Standard length and total length values (mm) from FishBase and log_10_ body size are provided. Asterisks indicated published SL measurements not available from FishBase (see Methods, López-Fernández et al. 2013)

| Tribe | Species | Body Size (mm) |  | Log Body Size |
| --- | --- | --- | --- | --- |
| Astronotini | *Astronotus crassipinnis* | 240 | SL | 2.38 |
|  | *Astronotus ocellatus* | 457 | TL | 2.66 |
| Chaetobranchini | *Chaetobranchopsis australis* | 120 | SL | 2.079 |
|  | *Chaetobranchopsis orbicularis* | 120 | SL | 2.079 |
|  | *Chaetobranchus flavescens* | 210 | SL | 2.415 |
|  | *Chaetobranchus semifasciatus* | 230 | SL | 2.362 |
| Cichlasomatini | *Andinoacara sapayensis* | 100 | TL | 2 |
|  | *Andinoacara stalsbergi* | 113 | SL | 2.053 |
|  | *Bujurquina apoparuana* | 77 | SL | 1.886 |
|  | *Bujurquina cordemadi* | 62 | SL | 1.792 |
|  | *Bujurquina eurhinus* | 89 | SL | 1.949 |
|  | *Bujurquina hophrys* | 112* | SL | 2.049 |
|  | *Bujurquina huallagae* | 83 | SL | 1.919 |
|  | *Bujurquina labiosa* | 55 | SL | 1.74 |
|  | *Bujurquina mariae* | 150 | TL | 2.176 |
|  | *Bujurquina megalospilus* | 71 | SL | 1.851 |
|  | *Bujurquina moriorum* | 97 | SL | 1.987 |
|  | *Bujurquina oenolaemus* | 67 | SL | 1.826 |
|  | *Bujurquina ortegai* | 110 | SL | 2.041 |
|  | *Bujurquina peregrinabunda* | 107 | SL | 2.029 |
|  | *Bujurquina robustus* | 88 | SL | 1.944 |
|  | *Bujurquina syspilus* | 103 | SL | 2.013 |
|  | *Bujurquina tambopatae* | 82 | SL | 1.914 |
|  | *Bujurquina vittata* | 90 | SL | 1.954 |
|  | *Bujurquina zamorensis* | 74 | SL | 1.869 |
|  | *Tahuantinsuyoa chipi* | 82 | SL | 1.914 |
|  | *Tahuantinsuyoa macantzatza* | 120 | TL | 2.079 |
|  | *Aequidens biseriatus* | 80 | SL | 1.903 |
|  | *Aequidens chimantanus* | 102 | SL | 2.009 |
|  | *Aequidens coeruleopunctatus* | 145 | TL | 2.161 |
|  | *Aequidens diadema* | 118 | SL | 2.072 |
|  | *Aequidens epae* | 113 | SL | 2.053 |
|  | *Aequidens gerciliae* | 128 | SL | 2.107 |
|  | *Aequidens hoehnei* | 56 | SL | 1.748 |
|  | *Aequidens latifrons* | 170 | TL | 2.23 |
|  | *Aequidens mauesanus* | 134 | SL | 2.127 |
|  | *Aequidens metae* | 125 | SL | 2.097 |
|  | *Aequidens pallidus* | 143 | SL | 2.155 |
|  | *Aequidens paloemeuensis* | 95 | SL | 1.978 |
|  | *Aequidens patricki* | 116 | SL | 2.064 |
|  | *Aequidens plagiozonatus* | 103 | SL | 2.013 |
|  | *Aequidens potaroensis* | 100 | SL | 2 |
|  | *Aequidens pulcher (Andinoacara)* | 107* | SL | 2.029 |
|  | *Aequidens rivulatus* | 200 | TL | 2.301 |
|  | *Aequidens rondoni* | 97 | SL | 1.987 |
|  | *Aequidens tetramerus* | 162 | SL | 2.21 |
|  | *Aequidens tubicen* | 116 | SL | 2.064 |
|  | *Aequidens viridis* | 165 | SL | 2.217 |
|  | *Cichlasoma amazonarum* | 114 | SL | 2.057 |
|  | *Cichlasoma araguaiense* | 92 | SL | 1.964 |
|  | *Cichlasoma bimaculatum* | 123 | SL | 2.09 |
|  | *Cichlasoma boliviense* | 107 | SL | 2.029 |
|  | *Cichlasoma dimerus* | 117 | SL | 2.068 |
|  | *Cichlasoma orientale* | 136 | SL | 2.134 |
|  | *Cichlasoma orinocense* | 109 | SL | 2.037 |
|  | *Cichlasoma paranaense* | 74 | SL | 1.869 |
|  | *Cichlasoma portalegrense* | 103 | SL | 2.013 |
|  | *Cichlasoma pusillum* | 66 | SL | 1.82 |
|  | *Cichlasoma sanctifranciscense* | 79 | SL | 1.898 |
|  | *Cichlasoma taenia* | 128 | SL | 2.107 |
|  | *Cichlasoma zarskei* | 100 | SL | 2 |
|  | *Krobia guianensis* | 128 | SL | 2.107 |
|  | *Krobia itanyi* | 125 | SL | 2.097 |
|  | *Krobia xinguensis* | 128* | SL | 2.107 |
|  | *Acaronia nassa* | 154 | SL | 2.188 |
|  | *Acaronia vultuosa* | 122 | SL | 2.086 |
|  | *Laetacara araguaiae* | 36 | SL | 1.556 |
|  | *Laetacara curviceps* | 46 | SL | 1.663 |
|  | *Laetacara dorsigera* | 60 | SL | 1.778 |
|  | *Laetacara flavilabris* | 82 | SL | 1.914 |
|  | *Laetacara fulcipinnis* | 74 | SL | 1.869 |
|  | *Laetacara thayeri* | 69* | SL | 1.839 |
|  | *Cleithracara maronii* | 71 | SL | 1.851 |
|  | *Nannacara adoketa* | 49 | SL | 1.69 |
|  | *Nannacara anomala* | 56 | SL | 1.748 |
|  | *Nannacara aureocephalus* | 67 | SL | 1.826 |
|  | *Nannacara bimaculata* | 45 | SL | 1.653 |
|  | *Nannacara taenia* | 50 | SL | 1.699 |
| Cichlini | *Cichla intermedia* | 375 | SL | 2.74 |
|  | *Cichla jariina* | 340 | SL | 2.531 |
|  | *Cichla kelberi* | 276 | SL | 2.441 |
|  | *Cichla melaniae* | 290 | SL | 2.462 |
|  | *Cichla mirianae* | 520 | SL | 2.716 |
|  | *Cichla monoculus* | 330 | SL | 2.845 |
|  | *Cichla nigromaculata* | 263 | SL | 2.42 |
|  | *Cichla ocellaris* | 740 | TL | 2.869 |
|  | *Cichla orinocensis* | 750* | SL | 2.79 |
|  | *Cichla pinima* | 520 | SL | 2.716 |
|  | *Cichla piquiti* | 430 | SL | 2.633 |
|  | *Cichla pleiozona* | 340 | SL | 2.531 |
|  | *Cichla temensis* | 990 | TL | 2.996 |
|  | *Cichla thyrorus* | 430 | SL | 2.633 |
|  | *Cichla vazzoleri* | 410 | SL | 2.613 |
| Geophagini- | *Apistogramma agassizii* | 43* | SL | 1.633 |
| CAS Clade | *Apistogramma alacrina* | 55 | SL | 1.74 |
|  | *Apistogramma amoena* | 63 | TL | 1.799 |
|  | *Apistogramma angayuara* | 25 | SL | 1.398 |
|  | *Apistogramma arua* | 44 | SL | 1.643 |
|  | *Apistogramma atahualpa* | 42 | SL | 1.623 |
|  | *Apistogramma baenschi* | 53 | SL | 1.724 |
|  | *Apistogramma barlowi* | 64 | SL | 1.806 |
|  | *Apistogramma bitaeniata* | 46 | SL | 1.663 |
|  | *Apistogramma borelli* | 39 | SL | 1.591 |
|  | *Apistogramma brevis* | 39 | SL | 1.591 |
|  | *Apistogramma cacatuoides* | 50 | SL | 1.699 |
|  | *Apistogramma caetei* | 36 | SL | 1.556 |
|  | *Apistogramma cinilabra* | 54 | SL | 1.732 |
|  | *Apistogramma commbrae* | 35 | TL | 1.519 |
|  | *Apistogramma cruzi* | 51 | SL | 1.708 |
|  | *Apistogramma diplotaenia* | 29 | SL | 1.462 |
|  | *Apistogramma elizabethae* | 40 | SL | 1.602 |
|  | *Apistogramma emnopygae* | 34 | SL | 1.531 |
|  | *Apistogramma erythrura* | 140 | SL | 2.146 |
|  | *Apistogramma eunotus* | 53 | SL | 1.724 |
|  | *Apistogramma geisleri* | 28 | SL | 1.447 |
|  | *Apistogramma gephyra* | 33 | SL | 1.519 |
|  | *Apistogramma gibbiceps* | 45 | SL | 1.653 |
|  | *Apistogramma gossei* | 44 | SL | 1.643 |
|  | *Apistogramma guttata* | 36 | SL | 1.556 |
|  | *Apistogramma hippolytae* | 34 | SL | 1.531 |
|  | *Apistogramma hoignei* | 60 | SL | 1.778 |
|  | *Apistogramma hongsloi* | 34 | SL | 1.531 |
|  | *Apistogramma huascar* | 45 | SL | 1.653 |
|  | *Apistogramma inconspicua* | 38 | SL | 1.58 |
|  | *Apistogramma iniridae* | 36 | SL | 1.556 |
|  | *Apistogramma juruensis* | 24 | SL | 1.38 |
|  | *Apistogramma linkei* | 39 | SL | 1.591 |
|  | *Apistogramma luelingi* | 33 | SL | 1.519 |
|  | *Apistogramma macmasteri* | 55 | SL | 1.74 |
|  | *Apistogramma megaptera* | 59 | SL | 1.771 |
|  | *Apistogramma meinkeni* | 35 | SL | 1.544 |
|  | *Apistogramma moae* | 50 | SL | 1.699 |
|  | *Apistogramma nijsseni* | 39 | SL | 1.591 |
|  | *Apistogramma norberti* | 39 | SL | 1.591 |
|  | *Apistogramma ortmanni* | 41 | SL | 1.613 |
|  | *Apistogramma panduro* | 49 | 49 | 1.69 |
|  | *Apistogramma pantalone* | 36 | SL | 1.556 |
|  | *Apistogramma paucisquamis* | 34 | SL | 1.531 |
|  | *Apistogramma payaminonis* | 40 | SL | 1.602 |
|  | *Apistogramma personata* | 49 | SL | 1.69 |
|  | *Apistogramma pertensis* | 39 | SL | 1.591 |
|  | *Apistogramma piauensis* | 23 | SL | 1.362 |
|  | *Apistogramma playayacu* | 49 | SL | 1.69 |
|  | *Apistogramma pleurotaenia* | 28 | SL | 1.447 |
|  | *Apistogramma pulchra* | 32 | SL | 1.505 |
|  | *Apistogramma regani* | 49 | SL | 1.69 |
|  | *Apistogramma resticulosa* | 27 | SL | 1.431 |
|  | *Apistogramma rositae* | 40 | SL | 1.602 |
|  | *Apistogramma rubrolineata* | 40 | SL | 1.602 |
|  | *Apistogramma rupununi* | 38 | SL | 1.58 |
|  | *Apistogramma salpinction* | 33 | SL | 1.519 |
|  | *Apistogramma staecki* | 21 | SL | 1.322 |
|  | *Apistogramma steindachneri* | 65 | SL | 1.813 |
|  | *Apistogramma taeniata* | 42 | SL | 1.623 |
|  | *Apistogramma trifasciata* | 38 | SL | 1.580 |
|  | *Apistogramma uaupesi* | 28 | SL | 1.447 |
|  | *Apistogramma urteagai* | 41 | SL | 1.613 |
|  | *Apistogramma viejita* | 30 | SL | 1.477 |
|  | *Apistogramma wapisana* | 29 | SL | 1.462 |
|  | *Apistogrammoides pucallpaensis (Apistogramma)* | 27 | SL | 1.431 |
|  | *Satanoperca acuticeps* | 170 | SL | 2.23 |
|  | *Satanoperca daemon* | 190 | SL | 2.279 |
|  | *Satanoperca jurupari* | 201* | SL | 2.303 |
|  | *Satanoperca leucosticta* | 160* | SL | 2.204 |
|  | *Satanoperca lilith* | 255 | SL | 2.407 |
|  | *Satanoperca mapiritensis* | 164* | SL | 2.215 |
|  | *Satanoperca pappaterra* | 174 | SL | 2.241 |
|  | *Satanoperca rhynchitis* | 141 | SL | 2.149 |
|  | *Taeniacara candidi* | 33 | SL | 1.519 |
|  | *Acarichthys heckelii* | 134 | SL | 2.127 |
|  | *Biotoecus dicentrarchus* | 38 | SL | 1.58 |
|  | *Biotoecus opercularis* | 100 | TL | 2 |
|  | *Crenicichla acutirostris* | 230 | TL | 2.362 |
|  | *Crenicichla adspersa* | 290 | SL | 2.462 |
|  | *Crenicichla albopunctata* | 140 | SL | 2.146 |
|  | *Crenicichla alta* | 160 | SL | 2.204 |
|  | *Crenicichla anthurus* | 224 | SL | 2.35 |
|  | *Crenicichla brasiliensis* | 78 | SL | 1.892 |
|  | *Crenicichla britskii* | 145 | SL | 2.161 |
|  | *Crenicichla cametana* | 183 | SL | 2.262 |
|  | *Crenicichla celidochilus* | 268 | TL | 2.428 |
|  | *Crenicichla cincta* | 195 | SL | 2.29 |
|  | *Crenicichla compressiceps* | 55 | SL | 1.74 |
|  | *Crenicichla coppenamensis* | 179 | SL | 2.253 |
|  | *Crenicichla cyanonotus* | 148 | SL | 2.17 |
|  | *Crenicichla cyclostoma* | 96 | SL | 1.982 |
|  | *Crenicichla empheres* | 141 | SL | 2.149 |
|  | *Crenicichla frenata* | 116 | SL | 2.064 |
|  | *Crenicichla gaucho* | 127 | SL | 2.104 |
|  | *Crenicichla geayi* | 136* | SL | 2.134 |
|  | *Crenicichla hadrostigma* | 114 | SL | 2.057 |
|  | *Crenicichla haroldoi* | 98 | SL | 1.991 |
|  | *Crenicichla heckeli* | 52 | SL | 1.716 |
|  | *Crenicichla hemera* | 97 | SL | 1.987 |
|  | *Crenicichla hu* | 153 | SL | 2.185 |
|  | *Crenicichla hummelincki* | 108 | SL | 2.033 |
|  | *Crenicichla igara* | 312 | TL | 2.494 |
|  | *Crenicichla iguapina* | 176 | SL | 2.246 |
|  | *Crenicichla iguassuensis* | 140 | SL | 2.146 |
|  | *Crenicichla inpa* | 168 | SL | 2.225 |
|  | *Crenicichla isbrueckeri* | 95 | SL | 1.978 |
|  | *Crenicichla jaguarensis* | 148 | SL | 2.17 |
|  | *Crenicichla jegui* | 200 | SL | 2.301 |
|  | *Crenicichla johanna* | 283 | SL | 2.452 |
|  | *Crenicichla jupiaensis* | 82 | SL | 1.914 |
|  | *Crenicichla jurubi* | 303 | TL | 2.481 |
|  | *Crenicichla labrina* | 160 | SL | 2.204 |
|  | *Crenicichla lacustris* | 290 | SL | 2.462 |
|  | *Crenicichla lenticulata* | 300 | SL | 2.477 |
|  | *Crenicichla lepidota* | 180 | SL | 2.255 |
|  | *Crenicichla lucius* | 168 | SL | 2.225 |
|  | *Crenicichla lugubris* | 260* | SL | 2.415 |
|  | *Crenicichla macropthalma* | 200 | SL | 2.301 |
|  | *Crenicichla maculata* | 247 | SL | 2.393 |
|  | *Crenicichla mandelburgeri* | 115 | SL | 2.061 |
|  | *Crenicichla marmorata* | 280 | SL | 2.447 |
|  | *Crenicichla menezesi* | 146 | SL | 2.164 |
|  | *Crenicichla minuano* | 260 | TL | 2.415 |
|  | *Crenicichla missioneira* | 283 | TL | 2.452 |
|  | *Crenicichla mucuryna* | 113 | SL | 2.053 |
|  | *Crenicichla multispinosa* | 228* | SL | 2.358 |
|  | *Crenicichla nickeriensis* | 191 | SL | 2.281 |
|  | *Crenicichla niederleinii* | 235 | SL | 2.371 |
|  | *Crenicichla notophthalmus* | 76 | SL | 1.881 |
|  | *Crenicichla pellegrini* | 157 | SL | 2.196 |
|  | *Crenicichla percna* | 220 | SL | 2.342 |
|  | *Crenicichla phaiospilus* | 240 | SL | 2.38 |
|  | *Crenicichla prenda* | 87 | SL | 1.94 |
|  | *Crenicichla proteus* | 155 | SL | 2.19 |
|  | *Crenicichla punctata* | 223 | SL | 2.348 |
|  | *Crenicichla pydanielae* | 178 | SL | 2.25 |
|  | *Crenicichla regani* | 79 | SL | 1.898 |
|  | *Crenicichla reticulata* | 216 | SL | 2.334 |
|  | *Crenicichla rosemariae* | 244 | SL | 2.387 |
|  | *Crenicichla santosi* | 120 | SL | 2.079 |
|  | *Crenicichla saxatilis* | 200 | SL | 2.301 |
|  | *Crenicichla scottii* | 169 | SL | 2.228 |
|  | *Crenicichla sedentaria* | 221 | SL | 2.344 |
|  | *Crenicichla semicincta* | 171 | SL | 2.233 |
|  | *Crenicichla semifasciata* | 150 | SL | 2.176 |
|  | *Crenicichla sipaliwini* | 173 | SL | 2.238 |
|  | *Crenicichla stocki* | 250 | TL | 2.398 |
|  | *Crenicichla strigata* | 300 | SL | 2.477 |
|  | *Crenicichla sveni* | 193* | SL | 2.286 |
|  | *Crenicichla tendyhaguassu* | 152 | SL | 2.182 |
|  | *Crenicichla ternetzi* | 245 | SL | 2.389 |
|  | *Crenicichla tigrina* | 280 | SL | 2.447 |
|  | *Crenicichla tingui* | 195 | SL | 2.29 |
|  | *Crenicichla urosema* | 68 | SL | 1.833 |
|  | *Crenicichla vaillanti* | 126 | SL | 2.1 |
|  | *Crenicichla virgatula* | 66 | SL | 1.82 |
|  | *Crenicichla vittata* | 260 | SL | 2.415 |
|  | *Crenicichla wallacii* | 85 | TL | 1.929 |
|  | *Crenicichla yaha* | 146 | SL | 2.164 |
|  | *Crenicichla ypo* | 137 | SL | 2.137 |
|  | *Crenicichla zebrina* | 264 | SL | 2.422 |
|  | *Teleocichla centisquama* | 40 | SL | 1.602 |
|  | *Teleocichla centrarchus* | 60 | SL | 1.778 |
|  | *Teleocichla cinderella* | 54 | SL | 1.732 |
|  | *Teleocichla gephyrogramma* | 57* | SL | 1.756 |
|  | *Teleocichla monogramma* | 63 | SL | 1.799 |
|  | *Teleocichla prionogenys* | 57 | SL | 1.756 |
|  | *Teleocichla proselytus* | 57 | SL | 1.756 |
|  | *Guianacara cuyunii* | 75 | SL | 1.875 |
|  | *Guianacara dacrya* | 120 | SL | 2.079 |
|  | *Guianacara geayi* | 85 | SL | 1.929 |
|  | *Guianacara oelemariensis* | 81 | SL | 1.908 |
|  | *Guianacara owroewefi* | 107 | SL | 2.029 |
|  | *Guianacara sphenozona* | 85 | SL | 1.929 |
|  | *Guianacara stergiosi* | 80 | SL | 1.903 |
|  | *Mazarunia mazarunii* | 77* | SL | 1.886 |
|  | *Mazarunia pala* | 74 | SL | 1.869 |
|  | *Mazarunia charadrica* | 84 | SL | 1.924 |
| Geophagini – GGD Clade | *Biotodoma cupido* | 97 | SL | 1.987 |
|  | *Biotodoma wavrini* | 104.5* | SL | 2.019 |
|  | *Crenicara latruncaularium* | 89 | SL | 1.949 |
|  | *Crenicara punctulatum* | 100 | SL | 2 |
|  | *Dicrossus filamentosus* | 38 | SL | 1.58 |
|  | *Dicrossus foirni* | 71 | SL | 1.851 |
|  | *Dicrossus gladicauda* | 42 | SL | 1.623 |
|  | *Dicrossus maculatus* | 53 | SL | 1.724 |
|  | *Dicrossus warzeli* | 60 | SL | 1.778 |
|  | *Geophagus abalios* | 211* | SL | 2.324 |
|  | *Geophagus altifrons* | 225 | SL | 2.352 |
|  | *Geophagus argyrostictus* | 180 | SL | 2.255 |
|  | *Geophagus brachybranchus* | 138 | SL | 2.14 |
|  | *Geophagus brokopondo* | 123 | SL | 2.09 |
|  | *Geophagus camopiensis* | 120 | SL | 2.079 |
|  | *Geophagus crassilabris* | 240 | SL | 2.38 |
|  | *Geophagus dicrozoster* | 205* | SL | 2.312 |
|  | *Geophagus gottwaldi* | 198 | SL | 2.297 |
|  | *Geophagus grammapareius* | 103 | SL | 2.013 |
|  | *Geophagus harreri* | 183 | SL | 2.262 |
|  | *Geophagus iporangensis* | 100 | SL | 2 |
|  | *Geophagus megasema* | 175 | SL | 2.243 |
|  | *Geophagus neambi* | 127 | SL | 2.104 |
|  | *Geophagus obscurus* | 100 | SL | 2 |
|  | *Geophagus parnaibae* | 76 | SL | 1.881 |
|  | *Geophagus pellegrini* | 152 | SL | 2.182 |
|  | *Geophagus proximus* | 225 | SL | 2.352 |
|  | *Geophagus steindachneri* | 198 | TL | 2.297 |
|  | *Geophagus surinamensis* | 148 | SL | 2.17 |
|  | *Geophagus sveni* | 167 | SL | 2.223 |
|  | *Geophagus taeniopareius* | 143 | SL | 2.155 |
|  | *Geophagus winemilleri* | 195 | SL | 2.29 |
|  | *Gymnogeophagus australis* | 155 | TL | 2.19 |
|  | *Gymnogeophagus caaguazuensis* | 86 | SL | 1.934 |
|  | *Gymnogeophagus che* | 116 | SL | 2.064 |
|  | *Gymnogeophagus gymnogenys* | 150 | SL | 2.176 |
|  | *Gymnogeophagus labiatus* | 120 | SL | 2.079 |
|  | *Gymnogeophagus lacustris* | 146 | SL | 2.164 |
|  | *Gymnogeophagus meridionalis* | 88 | SL | 1.944 |
|  | *Gymnogeophagus rhabdotus* | 120 | SL | 2.079 |
|  | *Gymnogeophagus setequedas* | 98 | SL | 1.991 |
|  | *Gymnogeophagus tiraparae* | 100 | SL | 2 |
|  | *Gynmogeophagus balzanii* | 120 | SL | 2.079 |
|  | *‘Geophagus’ brasiliensis* | 280 | TL | 2.447 |
|  | *Mikrogeophagus altispinosus* | 56 | SL | 1.748 |
|  | *Mikrogeophagus ramirezi* | 34 | SL | 1.531 |
| Heroini-  Central Americans | *"Cichlasoma" calobrensis (Amphilophus)* | 250 | TL | 2.398 |
|  | *"Cichlasoma" lyonsi (Amphilophus)* | 150 | SL | 2.176 |
|  | *"Cichlasoma" wesseli (Theraps)* | 80 | SL | 1.903 |
|  | *Amatitlania coatepeque* | 91 | SL | 1.959 |
|  | *Amatitlania kanna* | 83 | SL | 1.919 |
|  | *Amatitlania nigrofasciata* | 100 | SL | 2 |
|  | *Amatitlania siquia* | 79 | SL | 1.898 |
|  | *Amphilophus alfari* | 150 | SL | 2.176 |
|  | *Amphilophus altifrons* | 130 | SL | 2.114 |
|  | *Amphilophus amarillo* | 155 | SL | 2.19 |
|  | *Amphilophus astorquii* | 155 | SL | 2.19 |
|  | *Amphilophus bussingi* | 150 | SL | 2.176 |
|  | *Amphilophus chancho* | 242 | SL | 2.384 |
|  | *Amphilophus citrinellus* | 244 | SL | 2.387 |
|  | *Amphilophus flaveolus* | 136 | SL | 2.134 |
|  | *Amphilophus globosus* | 165 | SL | 2.217 |
|  | *Amphilophus hogaboomorum* | 150 | SL | 2.176 |
|  | *Amphilophus labiatus* | 240 | SL | 2.38 |
|  | *Amphilophus margaritifer* | 127 | SL | 2.104 |
|  | *Amphilophus nourissati (Theraps)* | 142* | SL | 2.152 |
|  | *Amphilophus rhytisma* | 135 | SL | 2.13 |
|  | *Amphilophus sagittae* | 160 | SL | 2.204 |
|  | *Amphilophus supercilius* | 168 | SL | 2.225 |
|  | *Amphilophus xiloaensis* | 160 | SL | 2.204 |
|  | *Amphilophus zaliosus* | 200 | TL | 2.301 |
|  | *Archocentrus centrarchus* | 110 | TL | 2.041 |
|  | *Archocentrus spinosissimus* | 110 | SL | 2.041 |
|  | *Australoheros acaroides* | 120 | SL | 2.079 |
|  | *Australoheros angiru* | 77 | SL | 1.886 |
|  | *Australoheros autrani* | 73 | SL | 1.863 |
|  | *Australoheros barbosae* | 96 | SL | 1.982 |
|  | *Australoheros capixaba* | 57 | SL | 1.756 |
|  | *Australoheros charrua* | 78 | SL | 1.892 |
|  | *Australoheros facetus* | 193 | SL | 2.286 |
|  | *Australoheros forquilha* | 110 | SL | 2.041 |
|  | *Australoheros guarani* | 129 | SL | 2.111 |
|  | *Australoheros ipatinguensis* | 53 | SL | 1.724 |
|  | *Australoheros kaaygua* | 94 | SL | 1.973 |
|  | *Australoheros macacuensis* | 83 | SL | 1.919 |
|  | *Australoheros macaensis* | 74 | SL | 1.869 |
|  | *Australoheros mattosi* | 80 | SL | 1.903 |
|  | *Australoheros minuano* | 84 | SL | 1.924 |
|  | *Australoheros montanus* | 103 | SL | 2.013 |
|  | *Australoheros muriae* | 121 | SL | 2.083 |
|  | *Australoheros paraibae* | 61 | SL | 1.785 |
|  | *Australoheros perdi* | 167 | TL | 2.223 |
|  | *Australoheros ribeirae* | 76 | SL | 1.881 |
|  | *Australoheros robustus* | 74 | SL | 1.869 |
|  | *Australoheros saquarema* | 80 | SL | 1.903 |
|  | *Australoheros scitulus* | 89 | SL | 1.949 |
|  | *Australoheros taura* | 124 | SL | 2.093 |
|  | *Australoheros tavaresi* | 79 | SL | 1.898 |
|  | *Australoheros tembe* | 134 | SL | 2.127 |
|  | *Australoheros ykeregua* | 137 | SL | 2.137 |
|  | *Cichlasoma salvini* | 220 | SL | 2.342 |
|  | *Cichlasoma trimaculatus* | 365 | SL | 2.562 |
|  | *Cryptoheros altoflavus* | 90 | SL | 1.954 |
|  | *Cryptoheros chetumalensis* | 97 | SL | 1.987 |
|  | *Cryptoheros cutteri* | 112 | SL | 2.049 |
|  | *Cryptoheros myrnae* | 80 | SL | 1.903 |
|  | *Cryptoheros nanoluteus* | 70* | SL | 1.845 |
|  | *Cryptoheros panamensis* | 130 | TL | 2.114 |
|  | *Cryptoheros sajica* | 90 | SL | 1.954 |
|  | *Cryptoheros septemfasciatus* | 100 | SL | 2 |
|  | *Cryptoheros spilurus* | 97 | TL | 1.987 |
|  | *Hypsophrys nematopus* | 140 | SL | 2.146 |
|  | *Hypsophrys nicaraguensis* | 165 | SL | 2.217 |
|  | *Parachromis dovii* | 500 | SL | 2.699 |
|  | *Parachromis friedrichsthalii* | 280 | SL | 2.447 |
|  | *Parachromis loisellei* | 185 | SL | 2.267 |
|  | *Parachromis managuensis* | 220 | SL | 2.342 |
|  | *Parachromis motaguensis* | 300 | TL | 2.477 |
|  | *Petenia splendida* | 500 | SL | 2.699 |
|  | *Astatheros diquis* | 135 | SL | 2.13 |
|  | *Astatheros longimanus* | 135 | SL | 2.13 |
|  | *Astatheros macracanthus* | 250 | SL | 2.398 |
|  | *Astatheros ocotal* | 96 | SL | 1.982 |
|  | *Astatheros octofasciata* | 250 | TL | 2.398 |
|  | *Astatheros robertsoni* | 190 | SL | 2.279 |
|  | *Astatheros rostratus* | 185 | SL | 2.267 |
|  | *Astatheros gemmata* | 70 | SL | 1.845 |
|  | *Caquetaia kraussii* | 260 | SL | 2.415 |
|  | *Caquetaia myersi* | 190 | SL | 2.279 |
|  | *Caquetaia spectabilis* | 165 | SL | 2.217 |
|  | *Caquetaia umbrifera* | 475 | SL | 2.677 |
|  | *Heroina isonycterina* | 102 | SL | 2.009 |
|  | *Cichlasoma aguadae* | 95 | SL | 1.978 |
|  | *Cichlasoma alborum* | 186 | SL | 2.27 |
|  | *Cichlasoma almarum* | 158 | SL | 2.199 |
|  | *Cichlasoma atromaculatum* | 250 | TL | 2.398 |
|  | *Cichlasoma beani* | 300 | TL | 2.477 |
|  | *Cichlasoma cienagae* | 113 | SL | 2.053 |
|  | *Cichlasoma conchitae* | 64 | SL | 1.806 |
|  | *Cichlasoma ericymba* | 122 | SL | 2.086 |
|  | *Cichlasoma geddesi* | 65 | TL | 1.813 |
|  | *Cichlasoma gephyrum* | 120 | SL | 2.079 |
|  | *Cichlasoma istlanum* | 300 | TL | 2.477 |
|  | *Cichlasoma mayorum* | 96 | SL | 1.982 |
|  | *Cichlasoma microlepis* | 187 | SL | 2.272 |
|  | *Cichlasoma ornatum* | 260 | SL | 2.415 |
|  | *Cichlasoma pearsei* | 200 | SL | 2.301 |
|  | *Cichlasoma stenozonum* | 110 | TL | 2.041 |
|  | *Cichlasoma troschelii* | 160 | SL | 2.204 |
|  | *Cichlasoma tuyrense* | 235 | SL | 2.371 |
|  | *Cichlasoma ufermanni* | 250 | SL | 2.398 |
|  | *Cichlasoma urophthalmum* | 300* | SL | 2.477 |
|  | *Cichlasoma zebra* | 104 | SL | 2.017 |
|  | *‘Cichlasoma' festae* | 250 | TL | 2.398 |
|  | *Cichlasoma grammodes* | 203 | SL | 2.307 |
|  | *Herichthys bartoni* | 180 | SL | 2.255 |
|  | *Herichthys carpintis* | 176* | SL | 2.246 |
|  | *Herichthys cyanoguttatus* | 300 | TL | 2.477 |
|  | *Herichthys deppii* | 120 | TL | 2.079 |
|  | *Herichthys labridens* | 250 | SL | 2.398 |
|  | *Herichthys minckleyi* | 175 | SL | 2.243 |
|  | *Herichthys pantostictus* | 126 | SL | 2.1 |
|  | *Herichthys steindachneri* | 400 | SL | 2.602 |
|  | *Herichthys tamasopoensis* | 180 | TL | 2.255 |
|  | *Paraneetroplus argenteus* | 270 | TL | 2.431 |
|  | *Paraneetroplus bifasciatus* | 300 | SL | 2.477 |
|  | *Paraneetroplus breidohri* | 168 | SL | 2.225 |
|  | *Paraneetroplus bulleri* | 255 | SL | 2.407 |
|  | *Paraneetroplus fenestratus* | 250 | SL | 2.398 |
|  | *Paraneetroplus gibbiceps* | 230 | SL | 2.362 |
|  | *Paraneetroplus guttulatus* | 300 | TL | 2.477 |
|  | *Paraneetroplus hartwegi* | 131 | SL | 2.117 |
|  | *Paraneetroplus maculicauda* | 250 | SL | 2.398 |
|  | *Paraneetroplus melanurus* | 190 | TL | 2.279 |
|  | *Paraneetroplus nebuliferus* | 200 | TL | 2.301 |
|  | *Paraneetroplus regani* | 230 | SL | 2.362 |
|  | *Paraneetroplus synspilus* | 350 | TL | 2.544 |
|  | *araneetroplus zonatus* | 250 | TL | 2.398 |
|  | *Theraps bocourti* | 200 | SL | 2.301 |
|  | *Theraps coeruleus* | 120 | SL | 2.079 |
|  | *Theraps godmanni* | 300 | TL | 2.477 |
|  | *Theraps heterospilus* | 240 | SL | 2.38 |
|  | *Theraps intermedius* | 200 | SL | 2.301 |
|  | *Theraps irregularis* | 190 | SL | 2.279 |
|  | *Theraps lentiginosus* | 250 | SL | 2.398 |
|  | *Theraps microphthalmus* | 250 | SL | 2.398 |
|  | *Thorichthys affinis* | 140 | SL | 2.146 |
|  | *Thorichthys aureus* | 150 | S/l | 2.176 |
|  | *Thorichthys callolepis* | 140 | SL | 2.146 |
|  | *Thorichthys ellioti* | 150 | SL | 2.176 |
|  | *Thorichthys helleri* | 145 | SL | 2.161 |
|  | *Thorichthys meeki* | 170 | TL | 2.23 |
|  | *Thorichthys pasionis* | 170 | SL | 2.23 |
|  | *Thorichthys socolofi* | 79 | SL | 1.898 |
|  | *Nandopsis haitiensis* | 215 | SL | 2.332 |
|  | *Nandopsis ramsdeni* | 240 | SL | 2.38 |
|  | *Nandopsis tetracanthus* | 200 | SL | 2.301 |
|  | *Archocentrus multispinosus (Herotilapia)* | 170 | TL | 2.23 |
|  | *Tomocichla asfraci* | 250 | SL | 2.398 |
|  | *Tomocichla sieboldii* | 250 | SL | 2.398 |
|  | *Tomocichla tuba* | 300 | TL | 2.477 |
| Heroini – South American | *Hoplarchus psittacus* | 235* | SL | 2.371 |
|  | *Hypselecara coryphaenoides* | 160 | SL | 2.204 |
|  | *Hypselecara temporalis* | 150 | SL | 2.176 |
|  | *Pterophyllum altum* | 180 | TL | 2.255 |
|  | *Pterophyllum leopoldi* | 50 | SL | 1.699 |
|  | *Pterophyllum scalare* | 75 | SL | 1.875 |
|  | *Heros efasciatus* | 140 | SL | 2.146 |
|  | *Heros notatus* | 150 | SL | 2.176 |
|  | *Heros severus* | 200 | SL | 2.301 |
|  | *Heros spurius* | 120 | SL | 2.079 |
|  | *Mesonauta acora* | 71 | SL | 1.851 |
|  | *Mesonauta egregius* | 82 | SL | 1.914 |
|  | *Mesonauta festivus* | 82 | SL | 1.914 |
|  | *Mesonauta guyanae* | 100 | SL | 2 |
|  | *Mesonauta insignis* | 94 | SL | 1.973 |
|  | *Mesonauta mirificus* | 97 | SL | 1.987 |
|  | *Symphysodon aequifasciatus* | 137 | SL | 2.137 |
|  | *Symphysodon discus* | 123 | SL | 2.09 |
|  | *Symphysodon tarzoo* | 132 | SL | 2.121 |
|  | *Uaru amphiacanthoides* | 250 | SL | 2.398 |
|  | *Uaru fernandezyepezi* | 190 | SL | 2.279 |
| Retroculini | *Retroculus lapidifer* | 203 | SL | 2.307 |
|  | *Retroculus septentrionalis* | 190 | SL | 2.279 |
|  | *Retroculus xinguensis* | 144 | SL | 2.158 |
