# Supplementary material for: Body Size Diversity and Frequency Distributions of Neotropical Cichlid Fishes (Cichliformes: Cichlidae: Cichlinae)
Source: PLoS One. 2014 Sep 2;9(9):e106336. doi: 10.1371/journal.pone.0106336 (PMC4152270; doi:10.1371/journal.pone.0106336)
Supplement: Table S2 — Data verification and description of data inconsistencies. Discrepancies between the maximum sizes on FishBase and maximum sizes in the original literature. (DOCX) [file pone.0106336.s003.docx]

**Table S2:** **Data verification and description of data inconsistencies.** Discrepancies (72 out of 498 species) between the maximum sizes on FishBase and maximum sizes in the original literature. All body size not reported here were cited from the Checklist of Freshwater Fishes of South and Central America (CLOFFSCA, Reis et al. 2003) and no discrepencies between FishBase and CLOFFSCA were found. Only 6 cases (in bold) were found where a clear error in the maximum body size was recorded on FishBase. Results were unchanged when analyses were rerun with corrected data. Other uncertainties in the data were due to inability to access the original source provided on FishBase (Unavailable source), female lengths without a direct reference and likely from various ecological or life history studies cited for each species on FishBase (Length of female specimen, uncited) or data was collected from angling records. For full citations see FishBase.

| Species | Max  Fishbase | CLOFFSCA | Note | Body Size Used  (App S1) |
| --- | --- | --- | --- | --- |
| Aequidens biseriatus | 120 | 80 | Length of female specimen, uncited | 80 |
| Apistogramma alacrina | 55 | - | Unavailable source^1^ | 55 |
| Apistogramma angayuara | 25 | - | Unavailable source^2^ | 25 |
| Apistogramma baenschi | 53 | - | Unavailable source^3^ | 53 |
| Apistogramma caetei | 40 | 36 | Length of female specimen, uncited | 36 |
| Apistogramma diplotaenia | 45 | 29 | Length of female specimen, uncited | 29 |
| Apistogramma gephyra | 40 | 33 | Length of female specimen, uncited | 33 |
| Apistogramma gibbiceps | 50 | 45 | Length of female specimen, uncited | 45 |
| Apistogramma gossei | 50 | 44 | Length of female specimen, uncited | 44 |
| Apistogramma hippolytae | 50 | 34 | Length of female specimen, uncited | 34 |
| Apistogramma hongsloi | 50 | 34 | Length of female specimen, uncited | 34 |
| Apistogramma iniridae | 50 | 36 | Length of female specimen, uncited | 36 |
| Apistogramma meinkeni | 45 | 35 | Length of female specimen, uncited | 35 |
| Apistogramma ortmanni | 55 | 41 | Length of female specimen, uncited | 41 |
| Apistogramma pertensis | 45 | 39 | Length of female specimen, uncited | 39 |
| Apistogramma pleurotaenia | 50 | 28 | Length of female specimen, uncited | 28 |
| Apistogramma resticulosa | 38 | 27 | Length of female specimen, uncited | 27 |
| Apistogramma rupununi | 50 | 38 | Length of female specimen, uncited | 38 |
| Apistogramma staecki | 40 | 21 | Length of female specimen, uncited | 21 |
| Apistogramma steindachneri | 70 | 65 | Length of female specimen, uncited | 65 |
| Apistogramma trifasciata | 46 | 38 | Length of female specimen, uncited | 38 |
| Apistogramma uaupesi | 60 | 28 | Length of female specimen, uncited | 28 |
| Apistogramma viejita | 46 | 30 | Length of female specimen, uncited | 30 |
| Archocentrus multispinosus | 170 | - | Unavailable source^4^ | 170 |
| Astronotus ocellatus | 457 | 210 | Angling record^5^ | 210 |
| **Australoheros capixaba** | **57** | **-** | **Holotype reported, rather than largest specimen at 120^6^** | **57** |
| **Australoheros forquilha** | **110** | **-** | **Holotype reported, rather than largest specimen at 130^7^** | **110** |
| Australoheros kaaygua | 94 | - | Length of female specimen, uncited | 94 |
| **Australoheros paraibae** | **61** | **-** | **More recent source, largest specimen 71^8^** | **61** |
| **Australoheros perdi** | **167** | **-** | **Possibly an error in data transfer, source reports largest at 122^9^** | **167** |
| **Australoheros scitulus** | **89** | **-** | **Holotype reported, rather than largest specimen at 97^10^** | **89** |
| Australoheros taura | 124 | - | Unavailable source^11^ | 124 |
| **Australoheros tavaresi** | **79** | **-** | **Possibly an error in data transfer, source reports largest at 66^12^** | **79** |
| Australoheros tembe | 134 | - | Unavailable source^13^ | 134 |
| Biotoecus opercularis | 100 | 38 | Unavailable source^14^ | 100 |
| Bujurquina vittata | 90 | 70 | Unavailable source^15^ | 90 |
| Chaetobranchus flavescens | 260 | 210 | Length of female specimen, uncited | 210 |
| Cichla intermedia | 550 | 375 | Angling record^5^ | 375 |
| Cichla jariina | 340 | - | Unavailable source^16^ | 340 |
| Cichla kelberi | 276 | - | Unavailable source^16^ | 276 |
| Cichla melaniae | 290 | - | Unavailable source^16^ | 290 |
| Cichla mirianae | 520 | - | Unavailable source^16^ | 520 |
| Cichla nigromaculata | 263 | - | Unavailable source^16^ | 263 |
| Cichla ocellaris | 740 | 419 | Angling record^5^ | 740 |
| Cichla pinima | 520 | - | Unavailable source^16^ | 520 |
| Cichla piquiti | 430 | - | Unavailable source^16^ | 430 |
| Cichla pleiozona | 340 | - | Unavailable source^16^ | 340 |
| Cichla temensis | 990 | 750 | Angling record^5^ | 990 |
| Cichla thyrorus | 430 | - | Unavailable source^16^ | 430 |
| Cichla vazzoleri | 410 | - | Unavailable source^16^ | 410 |
| Cichlasoma atromaculatum | 250 | 170 | Unavailable source^14^ | 250 |
| Cichlasoma bimaculatum | 300 | 123 | Length of female specimen, uncited | 123 |
| Cichlasoma urophthalmum | 394 | 300 | Angling record^5^ | 300 |
| Crenicichla celidochilus | 268 | 181 | Unavailable source^17^ | 268 |
| Crenicichla igara | 312 | 223 | Unavailable source^17^ | 312 |
| Crenicichla jurubi | 303 | 178 | Unavailable source^17^ | 303 |
| Crenicichla minuano | 260 | 172 | Unavailable source^17^ | 260 |
| Crenicichla missioneira | 283 | 209 | Unavailable source^17^ | 283 |
| Crenicichla sveni | 250 | 150 | Unavailable source^18^ | 193^23^ |
| Crenicichla yaha | 146 | - | Unavailable source^19^ | 146 |
| Dicrossus maculatus | 60 | 53 | Length of female specimen, uncited | 53 |
| Hoplarchus psittacus | 320 | - | Angling record^5^ | 235^23^ |
| Hypsophrys nicaraguensis | 200 | 165 | Length of female specimen, uncited | 165 |
| Laetacara curviceps | 66 | 46 | Length of female specimen, uncited | 46 |
| Laetacara dorsigera | 60 | 45 | Unavailable source^15^ | 60 |
| Laetacara thayeri | 120 | 65 | Unavailable source^20^ | 69^23^ |
| Parachromis dovii | 720 | 500 | Angling record^5^ | 500 |
| Parachromis managuensis | 550 | 220 | Angling record^5^ | 220 |
| Pterophyllum altum | 180 | 65 | Unavailable source^21^ | 180 |
| Satanoperca pappaterra | 192 | 174 | Unavailable source^15^ | 174^23^ |
| Taeniacara candidi | 50 | 33 | Length of female specimen, uncited | 33 |
| Tahuantinsuyoa macantzatza | 120 | 74 | Unavailable source^22^ | 120 |

^1^ Kullander 2004 Ichthyological Exploration of Freshwaters 15: 41-48

^2^ Kullander and Ferreira 2005 Neotropical Ichthyology 3(3): 361-371

^3^ Romer et al. 2004 Das Aquarium 422: 15-30

^4^ Riehl and Baensch 1991 Aquarien Atlas 1: 992

^5^ Database of IGFA angling records until 2001

^6^ Ottoni 2010 Vertebrate Zoology 60:19-25

^7^ Rícan and Kullander 2008 Zootaxa 1724:1-51

^8^ Ottoni et al. 2008 Vertebrate Zoology 58:207-232

^9^ Ottoni et al. 2011 Vertebrate Zoology 61: 137-145

^10^ Rícan and Kullander 2003 Copeia 2003: 794-802

^11^ Ottoni and Cheffe 2009 Spixiana 32:153-159

^12^ Ottoni 2012 Vertebrate Zoology 62: 83-96

^13^ Casciotta et al. 1995 Ichthyological Exploration of Freshwaters

^14^ Axelrod 1993 The most complete colored lexicon of cichlids

^15^ Britski et al. 2007 Peixes do Pantanal

^16^ Kullander and Ferreira 2006 Ichthyological Exploration of Freshwaters 17(4): 289-398

^17^ Filho et al. 2004 Catálogo ilustrado de peixes do alto Rio Uruguai

^18^ Baensch and Riehl 1995 Aquarien Atlas

^19^ Casciotta et al. 2006 Zoologische Abhabdlungen 56:107-112

^20^ Stawikowski and Werner 1998 Die Buntbarsche Amerikas

^21^ Riehl and Baensch 1996 Aquarien Atlas

^22^ Baensch and Riehl 1997 Aquarien Atlas

^23^ López-Fernández et al. 2013 Evolution 67: 1321-1337
