# Supplementary material for: Body Size Diversity and Frequency Distributions of Neotropical Cichlid Fishes (Cichliformes: Cichlidae: Cichlinae)
Source: PLoS One. 2014 Sep 2;9(9):e106336. doi: 10.1371/journal.pone.0106336 (PMC4152270; doi:10.1371/journal.pone.0106336)
Supplement: Table S3 — Testing for effects of TL data in quantifying cichlid body size. Changes to the summary statistics and bootstrapping results when species represented by total length (TL) data were removed from the analyses. (DOCX) [file pone.0106336.s004.docx]

**Table S3: Testing for effects of TL data in quantifying cichlid body size.** Changes to the summary statistics and bootstrapping results when species represented by total length (TL) data were removed from the analyses. For discussion of results see Appendix S1. An asterisk next to subclade names indicates a significant difference from the more inclusive clade above (Kolmogorov-Smirnov Test). An asterisk next to statistical values indicates significantly different values as compared to a random phylogenetic distribution attained by bootstrap simulations (* indicates p<0.05; ** indicates p<0.005). Direction of deviation can be found by comparing the value of summary statistics between clades and respective subclades. Number of species (N); Standard Deviation (St Dev); Interquartile Range (IQR).

| Clade | N | Mean | St Dev | Min | Max | 25% Q | 75% Q | Kurtosis | Skew | IQR |
| --- | --- | --- | --- | --- | --- | --- | --- | --- | --- | --- |
|  |  |  |  |  |  |  |  |  |  |  |
| Heroini (Tribe)* | 176 | 2.18** | 0.21** | 1.70** | 2.70 | 2.01** | 2.35* | -0.57 | -0.014 | 0.34 |
| Without TL | 142 | 2.15** | 0.20** | 1.70** | 2.70 | 1.98** | 2.28 | -0.37 | 0.12 | 0.30 |
|  |  |  |  |  |  |  |  |  |  |  |
| SA Heroines | 21 | 2.09* | 0.18 | 1.70 | 2.40 | 1.97 | 2.20 | -0.81 | -0.22 | 0.23 |
| Without TL | 20 | 2.08* | 0.18 | 1.70 | 1.96** | 1.96 | 2.18 | -0.74 | -0.23 | 0.22 |
|  |  |  |  |  |  |  |  |  |  |  |
| CA Heroines | 153 | 2.19* | 0.21 | 1.72 | 2.70 | 2.04 | 2.38* | -0.63 | -0.01 | 0.34 |
| Without TL | 119 | 2.15** | 0.20 | 1.72 | 2.70 | 1.98 | 2.28** | -0.40 | 0.17 | 0.30 |
