# Supplementary material for: Body Size Diversity and Frequency Distributions of Neotropical Cichlid Fishes (Cichliformes: Cichlidae: Cichlinae)
Source: PLoS One. 2014 Sep 2;9(9):e106336. doi: 10.1371/journal.pone.0106336 (PMC4152270; doi:10.1371/journal.pone.0106336)
Supplement: Table S4 — Summary statistics of cichlid body size distributions and expectation under random phylogenetic distribution. (DOCX) [file pone.0106336.s005.docx]

**Table S4:** **Summary statistics of cichlid body size distributions and expectation under random phylogenetic distribution.** Comparison of clade observed summary statistics to distributions of summary statistics describing 1000 random simulated data from containing clades (expected values, see Methods) at higher taxonomic levels. Upper and lower significance thresholds of simulated expected distributions are provided for P values <0.05 (2.50%-97.50%) and <0.005 (0.25%-99.75%). Observed values that significantly differed from simulated statistics are given in bold.

| Statisitic | | Lower Tail | | Upper Tail | | Lower Tail | | | Upper Tail | | Observed | |  | | Statistic | Lower Tail | Upper Tail | | | Lower Tail | Upper Tail | | Observed |
| --- | --- | --- | --- | --- | --- | --- | --- | --- | --- | --- | --- | --- | --- | --- | --- | --- | --- | --- | --- | --- | --- | --- | --- |
| Genera Comparisons with simulations using Major Clade (if applicable) and Tribe body size distributions | | | | | | | | | | | | | | | | | | | |  |  | |  |
| Apistogramma-CAS | | | |  | |  |  | |  | |  | | Apistogramma-Geophagini | | | | |  | |  |  | |  |
|  | | 2.50% | | 97.50% | | 0.25% | 99.75% | | Observed | |  | |  | | 2.50% | | 97.50% | 0.25% | | 99.75% | Observed | |  |
| Mean | | 1.88099 | | 2.002073 | | 1.853836 | 2.031912 | | **1.600015** | |  | | Mean | | 1.905897 | | 2.031817 | 1.883388 | | 2.060432 | **1.600015** | |  |
| St Dev | | 0.305226 | | 0.356708 | | 0.292573 | 0.364807 | | **0.130845** | |  | | St Dev | | 0.287029 | | 0.342857 | 0.277998 | | 0.351831 | **0.130845** | |  |
| Minimum | | 1.322219 | | 1.447158 | | 1.322219 | 1.491206 | | 1.322219 | |  | | Minimum | | 1.322219 | | 1.462398 | 1.322219 | | 1.518514 | 1.322219 | |  |
| Maximum | | 2.451786 | | 2.494155 | | 2.428135 | 2.494155 | | **2.146128** | |  | | Maximum | | 2.447158 | | 2.494155 | 2.410736 | | 2.494155 | **2.146128** | |  |
| 25% Quantile | | 1.591065 | | 1.724276 | | 1.562143 | 1.757613 | | **1.524996** | |  | | 25% Quantile | | 1.60206 | | 1.788746 | 1.58823 | | 1.88506 | **1.524996** | |  |
| 75% Quantile | | 2.16286 | | 2.295532 | | 2.141424 | 2.338438 | | **1.690196** | |  | | 75% Quantile | | 2.167307 | | 2.295532 | 2.146897 | | 2.310212 | **1.690196** | |  |
| Kurtosis | | -1.55525 | | -1.18554 | | -1.59784 | -1.06313 | | **2.926193** | |  | | Kurtosis | | -1.49742 | | -0.93914 | -1.56463 | | -0.69681 | **2.926193** | |  |
| Skew | | -0.26172 | | 0.30685 | | -0.37602 | 0.447004 | | **0.874964** | |  | | Skew | | -0.45935 | | 0.14217 | -0.56711 | | 0.289576 | **0.874964** | |  |
| IQR | | 0.510938 | | 0.675879 | | 0.485518 | 0.706901 | | **0.1652** | |  | | IQR | | 0.448239 | | 0.644102 | 0.383142 | | 0.682835 | **0.1652** | |  |
|  | |  | |  | |  |  | |  | |  | |  | |  | |  |  | |  |  | |  |
| Biotodoma-GGD | | | |  | |  |  | |  | |  | | Biotodoma-Geophagini | | | | |  | |  |  | |  |
|  | | 2.50% | | 97.50% | | 0.25% | 99.75% | | Observed | |  | |  | | 2.50% | | 97.50% | 0.25% | | 99.75% | Observed | |  |
| Mean | | 1.759076 | | 2.31881 | | 1.639833 | 2.368596 | | 2.002944 | |  | | Mean | | 1.553362 | | 2.347477 | 1.451001 | | 2.438443 | 2.002944 | |  |
| St Dev | | 0.006204 | | 0.477643 | | 0 | 0.581464 | | 0.022871 | |  | | St Dev | | 0.009536 | | 0.626613 | 0 | | 0.718854 | 0.022871 | |  |
| Minimum | | 1.531479 | | 2.290035 | | 1.531479 | 2.352183 | | 1.986772 | |  | | Minimum | | 1.380211 | | 2.296774 | 1.322219 | | 2.40654 | 1.986772 | |  |
| Maximum | | 1.851258 | | 2.447158 | | 1.724276 | 2.447158 | | 2.019116 | |  | | Maximum | | 1.591065 | | 2.462766 | 1.524964 | | 2.494155 | 2.019116 | |  |
| 25% Quantile | | 1.654457 | | 2.300437 | | 1.585656 | 2.355704 | | 1.994858 | |  | | 25% Quantile | | 1.506319 | | 2.314419 | 1.388013 | | 2.41776 | 1.994858 | |  |
| 75% Quantile | | 1.819435 | | 2.361364 | | 1.688153 | 2.407877 | | 2.01103 | |  | | 75% Quantile | | 1.572875 | | 2.398607 | 1.503548 | | 2.459582 | 2.01103 | |  |
| Kurtosis | | -2.75 | | -2.75 | | -2.75 | 0 | | -2.75 | |  | | Kurtosis | | -2.75 | | -2.75 | -2.75 | | 0 | -2.75 | |  |
| Skew | | -9E-15 | | 8.48E-15 | | -3.7E-14 | 5.21E-14 | | 0 | |  | | Skew | | -7.4E-15 | | 5.09E-15 | -2.7E-14 | | 6.64E-14 | 0 | |  |
| IQR | | 0.004387 | | 0.337745 | | 0 | 0.411157 | | 0.016172 | |  | | IQR | | 0.006743 | | 0.443082 | 0 | | 0.508306 | 0.016172 | |  |
|  | |  | |  | |  |  | |  | |  | |  | |  | |  |  | |  |  | |  |
| Biotoecus-GGD | | | |  | |  |  | |  | |  | | Biotoecus-Geophagini | | | | |  | |  |  | |  |
|  | | 2.50% | | 97.50% | | 0.25% | 99.75% | | Observed | |  | |  | | 2.50% | | 97.50% | 0.25% | | 99.75% | Observed | |  |
| Mean | | 1.775298 | | 2.334937 | | 1.65203 | 2.39967 | | 1.789892 | |  | | Mean | | 1.553362 | | 2.347477 | 1.451001 | | 2.438443 | 1.789892 | |  |
| St Dev | | 0.008651 | | 0.476177 | | 0 | 0.556189 | | 0.297138 | |  | | St Dev | | 0.009536 | | 0.626613 | 0 | | 0.718854 | 0.297138 | |  |
| Minimum | | 1.531479 | | 2.296665 | | 1.531479 | 2.352183 | | 1.579784 | |  | | Minimum | | 1.380211 | | 2.296774 | 1.322219 | | 2.40654 | 1.579784 | |  |
| Maximum | | 1.933156 | | 2.447158 | | 1.724276 | 2.447158 | | 2 | |  | | Maximum | | 1.591065 | | 2.462766 | 1.524964 | | 2.494155 | 2 | |  |
| 25% Quantile | | 1.668404 | | 2.314953 | | 1.615907 | 2.375926 | | 1.684838 | |  | | 25% Quantile | | 1.506319 | | 2.314419 | 1.388013 | | 2.41776 | 1.684838 | |  |
| 75% Quantile | | 1.856648 | | 2.359325 | | 1.688153 | 2.423414 | | 1.894946 | |  | | 75% Quantile | | 1.572875 | | 2.398607 | 1.503548 | | 2.459582 | 1.894946 | |  |
| Kurtosis | | -2.75 | | -2.75 | | -2.75 | 0 | | -2.75 | |  | | Kurtosis | | -2.75 | | -2.75 | -2.75 | | 0 | -2.75 | |  |
| Skew | | -9.6E-15 | | 8.49E-15 | | -4.1E-14 | 3.16E-14 | | 0 | |  | | Skew | | -7.4E-15 | | 5.09E-15 | -2.7E-14 | | 6.64E-14 | 0 | |  |
| IQR | | 0.006117 | | 0.336708 | | 0 | 0.393285 | | 0.210108 | |  | | IQR | | 0.006743 | | 0.443082 | 0 | | 0.508306 | 0.210108 | |  |
|  | |  | |  | |  |  | |  | |  | |  | |  | |  |  | |  |  | |  |
| Crenicara-GGD | | | |  | |  |  | |  | |  | | Crenicara-Geophaginni | | | | |  | |  |  | |  |
|  | | 2.50% | | 97.50% | | 0.25% | 99.75% | | Observed | |  | |  | | 2.50% | | 97.50% | 0.25% | | 99.75% | Observed | |  |
| Mean | | 1.740434 | | 2.338232 | | 1.601516 | 2.39967 | | 1.974695 | |  | | Mean | | 1.553362 | | 2.347477 | 1.451001 | | 2.438443 | 1.974695 | |  |
| St Dev | | 0.007039 | | 0.473174 | | 0 | 0.598036 | | 0.035787 | |  | | St Dev | | 0.009536 | | 0.626613 | 0 | | 0.718854 | 0.035787 | |  |
| Minimum | | 1.531479 | | 2.296665 | | 1.531479 | 2.352183 | | 1.94939 | |  | | Minimum | | 1.380211 | | 2.296774 | 1.322219 | | 2.40654 | 1.94939 | |  |
| Maximum | | 1.851258 | | 2.447158 | | 1.623249 | 2.447158 | | 2 | |  | | Maximum | | 1.591065 | | 2.462766 | 1.524964 | | 2.494155 | 2 | |  |
| 25% Quantile | | 1.651738 | | 2.317552 | | 1.579678 | 2.375926 | | 1.962043 | |  | | 25% Quantile | | 1.506319 | | 2.314419 | 1.388013 | | 2.41776 | 1.962043 | |  |
| 75% Quantile | | 1.819435 | | 2.363097 | | 1.612383 | 2.423414 | | 1.987348 | |  | | 75% Quantile | | 1.572875 | | 2.398607 | 1.503548 | | 2.459582 | 1.987348 | |  |
| Kurtosis | | -2.75 | | -2.75 | | -2.75 | 0 | | -2.75 | |  | | Kurtosis | | -2.75 | | -2.75 | -2.75 | | 0 | -2.75 | |  |
| Skew | | -7.6E-15 | | 9.86E-15 | | -3.7E-14 | 7.5E-14 | | 4.62E-15 | |  | | Skew | | -7.4E-15 | | 5.09E-15 | -2.7E-14 | | 6.64E-14 | 4.62E-15 | |  |
| IQR | | 0.004977 | | 0.334584 | | 0 | 0.422875 | | 0.025305 | |  | | IQR | | 0.006743 | | 0.443082 | 0 | | 0.508306 | 0.025305 | |  |
|  | |  | |  | |  |  | |  | |  | |  | |  | |  |  | |  |  | |  |
| Crenicichla-CAS | | | |  | |  |  | |  | |  | | Crenicichla-Geophagini | | | | |  | |  |  | |  |
|  | | 2.50% | | 97.50% | | 0.25% | 99.75% | | Observed | |  | |  | | 2.50% | | 97.50% | 0.25% | | 99.75% | Observed | |  |
| Mean | | 1.896661 | | 1.994681 | | 1.873878 | 2.018001 | | **2.176721** | |  | | Mean | | 1.918536 | | 2.02527 | 1.89233 | | 2.043222 | **2.176721** | |  |
| St Dev | | 0.310641 | | 0.351778 | | 0.299824 | 0.358612 | | **0.221959** | |  | | St Dev | | 0.292045 | | 0.336912 | 0.276278 | | 0.343901 | **0.221959** | |  |
| Minimum | | 1.322219 | | 1.431759 | | 1.322219 | 1.447158 | | **1.60206** | |  | | Minimum | | 1.322219 | | 1.447158 | 1.322219 | | 1.518514 | **1.60206** | |  |
| Maximum | | 2.462398 | | 2.494155 | | 2.449461 | 2.494155 | | 2.494155 | |  | | Maximum | | 2.451786 | | 2.494155 | 2.424853 | | 2.494155 | 2.494155 | |  |
| 25% Quantile | | 1.591065 | | 1.713895 | | 1.588244 | 1.738371 | | **2.054992** | |  | | 25% Quantile | | 1.60206 | | 1.772582 | 1.591065 | | 1.822773 | **2.054992** | |  |
| 75% Quantile | | 2.178241 | | 2.290103 | | 2.162114 | 2.301572 | | **2.354091** | |  | | 75% Quantile | | 2.176091 | | 2.290035 | 2.154914 | | 2.301302 | **2.354091** | |  |
| Kurtosis | | -1.51658 | | -1.23088 | | -1.56731 | -1.14964 | | **-0.530983** | |  | | Kurtosis | | -1.45459 | | -0.98453 | -1.51754 | | -0.73825 | **-0.53098** | |  |
| Skew | | -0.25421 | | 0.224107 | | -0.31041 | 0.324134 | | **-0.626799** | |  | | Skew | | -0.39227 | | 0.100312 | -0.55367 | | 0.185719 | **-0.6268** | |  |
| IQR | | 0.525637 | | 0.662519 | | 0.505976 | 0.695238 | | **0.2991** | |  | | IQR | | 0.467412 | | 0.634905 | 0.40377 | | 0.666014 | **0.2991** | |  |
|  | |  | |  | |  |  | |  | |  | |  | |  | |  |  | |  |  | |  |
| Dicrossus-GGD | | | |  | |  |  | |  | |  | | Dicrossus-Geophagini | | | | |  | |  |  | |  |
|  | | 2.50% | | 97.50% | | 0.25% | 99.75% | | Observed | |  | |  | | 2.50% | | 97.50% | 0.25% | | 99.75% | Observed | |  |
| Mean | | 1.888216 | | 2.234201 | | 1.814692 | 2.285165 | | **1.711344** | |  | | Mean | | 1.695395 | | 2.236477 | 1.622017 | | 2.343663 | 1.711344 | |  |
| St Dev | | 0.078692 | | 0.339278 | | 0.039232 | 0.371127 | | 0.11099 | |  | | St Dev | | 0.119418 | | 0.461478 | 0.065922 | | 0.516157 | **0.11099** | |  |
| Minimum | | 1.531479 | | 2.079181 | | 1.531479 | 2.180347 | | 1.579784 | |  | | Minimum | | 1.322219 | | 2.029384 | 1.322219 | | 2.193038 | 1.579784 | |  |
| Maximum | | 2.089905 | | 2.447158 | | 2 | 2.447158 | | **1.851258** | |  | | Maximum | | 1.991226 | | 2.481443 | 1.763326 | | 2.494155 | **1.851258** | |  |
| 25% Quantile | | 1.623249 | | 2.190332 | | 1.579784 | 2.293367 | | 1.623249 | |  | | 25% Quantile | | 1.51818 | | 2.204388 | 1.431364 | | 2.331097 | 1.623249 | |  |
| 75% Quantile | | 2 | | 2.352183 | | 1.907522 | 2.380211 | | **1.778151** | |  | | 75% Quantile | | 1.724276 | | 2.421604 | 1.573597 | | 2.477121 | 1.778151 | |  |
| Kurtosis | | -2.20755 | | -1.08937 | | -2.24257 | -0.98199 | | -1.994462 | |  | | Kurtosis | | -2.22799 | | -1.06405 | -2.24899 | | -0.95263 | -1.99446 | |  |
| Skew | | -0.92095 | | 0.719846 | | -1.0194 | 0.994721 | | 0.011499 | |  | | Skew | | -0.91402 | | 0.865578 | -1.04584 | | 1.013138 | 0.011499 | |  |
| IQR | | 0.036182 | | 0.514322 | | 0 | 0.629544 | | 0.154902 | |  | | IQR | | 0.048784 | | 0.792099 | 0.011431 | | 0.879122 | 0.154902 | |  |
|  | |  | |  | |  |  | |  | |  | |  | |  | |  |  | |  |  | |  |
| Geophagus-GGD | | | |  | |  |  | |  | |  | | Geophagus-Geophagini | | | | |  | |  |  | |  |
|  | | 2.50% | | 97.50% | | 0.25% | 99.75% | | Observed | |  | |  | | 2.50% | | 97.50% | 0.25% | | 99.75% | Observed | |  |
| Mean | | 2.012402 | | 2.135287 | | 2.000015 | 2.15316 | | **2.191362** | |  | | Mean | | 1.848741 | | 2.086323 | 1.807096 | | 2.12723 | **2.191362** | |  |
| St Dev | | 0.157671 | | 0.257541 | | 0.139764 | 0.272632 | | **0.134981** | |  | | St Dev | | 0.252198 | | 0.366973 | 0.237651 | | 0.393613 | **0.134981** | |  |
| Minimum | | 1.531479 | | 1.778151 | | 1.531479 | 1.934499 | | **1.880814** | |  | | Minimum | | 1.322219 | | 1.579784 | 1.322219 | | 1.607449 | **1.880814** | |  |
| Maximum | | 2.323969 | | 2.447158 | | 2.296665 | 2.447158 | | 2.380211 | |  | | Maximum | | 2.342423 | | 2.494155 | 2.25394 | | 2.494155 | 2.380211 | |  |
| 25% Quantile | | 1.866036 | | 2.032303 | | 1.814705 | 2.079181 | | **2.096854** | |  | | 25% Quantile | | 1.567566 | | 1.940452 | 1.531479 | | 2.040468 | **2.096854** | |  |
| 75% Quantile | | 2.15507 | | 2.293433 | | 2.119539 | 2.30421 | | **2.296665** | |  | | 75% Quantile | | 2.07182 | | 2.349276 | 1.996842 | | 2.391058 | 2.296665 | |  |
| Kurtosis | | -1.0993 | | 1.014651 | | -1.36849 | 1.841599 | | -0.792653 | |  | | Kurtosis | | -1.7052 | | -0.56228 | -1.80344 | | 0.562164 | -0.79265 | |  |
| Skew | | -1.05939 | | -0.00018 | | -1.21653 | 0.394111 | | -0.525809 | |  | | Skew | | -0.73346 | | 0.376185 | -1.09885 | | 0.565531 | -0.52581 | |  |
| IQR | | 0.177076 | | 0.353859 | | 0.156404 | 0.447131 | | 0.199811 | |  | | IQR | | 0.308814 | | 0.684604 | 0.231638 | | 0.75893 | **0.199811** | |  |
|  | |  | |  | |  |  | |  | |  | |  | |  | |  |  | |  |  | |  |
| Guianacara-CAS | | | |  | |  |  | |  | |  | | Guianacara-Geophagini | | | | |  | |  |  | |  |
|  | | 2.50% | | 97.50% | | 0.25% | 99.75% | | Observed | |  | |  | | 2.50% | | 97.50% | 0.25% | | 99.75% | Observed | |  |
| Mean | | 1.701192 | | 2.175673 | | 1.611909 | 2.261969 | | 1.950577 | |  | | Mean | | 1.739392 | | 2.193717 | 1.620713 | | 2.269875 | 1.950577 | |  |
| St Dev | | 0.189248 | | 0.437036 | | 0.096756 | 0.474941 | | **0.074581** | |  | | St Dev | | 0.161946 | | 0.421566 | 0.09782 | | 0.454359 | **0.074581** | |  |
| Minimum | | 1.322219 | | 1.778681 | | 1.322219 | 2.058797 | | **1.875061** | |  | | Minimum | | 1.322219 | | 1.881234 | 1.322219 | | 2.071856 | 1.875061 | |  |
| Maximum | | 2.146128 | | 2.494155 | | 1.869079 | 2.494155 | | **2.079181** | |  | | Maximum | | 2.136641 | | 2.494155 | 1.877818 | | 2.494155 | **2.079181** | |  |
| 25% Quantile | | 1.496939 | | 2.099147 | | 1.447158 | 2.223585 | | 1.905788 | |  | | 25% Quantile | | 1.519096 | | 2.121767 | 1.455719 | | 2.220723 | 1.905788 | |  |
| 75% Quantile | | 1.776554 | | 2.404519 | | 1.609892 | 2.445903 | | 1.979401 | |  | | 75% Quantile | | 1.853772 | | 2.396352 | 1.644231 | | 2.4409 | 1.979401 | |  |
| Kurtosis | | -2.11748 | | -0.09781 | | -2.16345 | 0.452478 | | -1.368941 | |  | | Kurtosis | | -2.09971 | | -0.25285 | -2.14223 | | 0.387777 | -1.36894 | |  |
| Skew | | -0.91598 | | 0.925349 | | -1.4051 | 1.378459 | | 0.683614 | |  | | Skew | | -0.98958 | | 0.775748 | -1.29552 | | 1.403441 | 0.683614 | |  |
| IQR | | 0.148767 | | 0.734796 | | 0.081776 | 0.864543 | | **0.073614** | |  | | IQR | | 0.132987 | | 0.703364 | 0.080477 | | 0.839469 | **0.073614** | |  |
|  | |  | |  | |  |  | |  | |  | |  | |  | |  |  | |  |  | |  |
| Gymnogeophagus-GGD | | | | | |  |  | |  | |  | | Gymnogeophagus-Geophagini | | | | |  | |  |  | |  |
|  | | 2.50% | | 97.50% | | 0.25% | 99.75% | | Observed | |  | |  | | 2.50% | | 97.50% | 0.25% | | 99.75% | Observed | |  |
| Mean | | 1.957529 | | 2.17637 | | 1.919091 | 2.234425 | | 2.063908 | |  | | Mean | | 1.791595 | | 2.149406 | 1.732055 | | 2.223072 | 2.063908 | |  |
| St Dev | | 0.123491 | | 0.284171 | | 0.099895 | 0.306444 | | **0.089294** | |  | | St Dev | | 0.213316 | | 0.391011 | 0.157113 | | 0.426962 | **0.089294** | |  |
| Minimum | | 1.531479 | | 1.950325 | | 1.531479 | 2.012837 | | 1.934498 | |  | | Minimum | | 1.322219 | | 1.716003 | 1.322219 | | 1.892008 | **1.934498** | |  |
| Maximum | | 2.243038 | | 2.447158 | | 2.121751 | 2.447158 | | **2.190332** | |  | | Maximum | | 2.225309 | | 2.494155 | 2.141401 | | 2.494155 | **2.190332** | |  |
| 25% Quantile | | 1.799424 | | 2.08529 | | 1.693172 | 2.163595 | | 1.995613 | |  | | 25% Quantile | | 1.537408 | | 2.068051 | 1.489884 | | 2.165079 | 1.995613 | |  |
| 75% Quantile | | 2.079181 | | 2.321191 | | 2.009558 | 2.338233 | | 2.121767 | |  | | 75% Quantile | | 1.941519 | | 2.37411 | 1.833228 | | 2.434381 | 2.121767 | |  |
| Kurtosis | | -1.68218 | | 0.930714 | | -1.83031 | 1.73563 | | -1.506751 | |  | | Kurtosis | | -1.9393 | | -0.09514 | -2.02704 | | 1.278109 | -1.50675 | |  |
| Skew | | -1.2199 | | 0.493364 | | -1.56288 | 0.952715 | | -0.007513 | |  | | Skew | | -0.95136 | | 0.647753 | -1.38872 | | 1.131487 | -0.00751 | |  |
| IQR | | 0.11139 | | 0.412541 | | 0.084168 | 0.513857 | | 0.126154 | |  | | IQR | | 0.196038 | | 0.707968 | 0.131351 | | 0.782529 | **0.126154** | |  |
|  | |  | |  | |  |  | |  | |  | |  | |  | |  |  | |  |  | |  |
| Mazarunia-CAS | | | |  | |  |  | |  | |  | | Mazarunia-Geophagini | | | | |  | |  |  | |  |
|  | | 2.50% | | 97.50% | | 0.25% | 99.75% | | Observed | |  | |  | | 2.50% | | 97.50% | 0.25% | | 99.75% | Observed | |  |
| Mean | | 1.578384 | | 2.292012 | | 1.508778 | 2.374565 | | 1.893334 | |  | | Mean | | 1.59635 | | 2.30265 | 1.496687 | | 2.381334 | 1.893334 | |  |
| St Dev | | 0.059771 | | 0.529352 | | 0.018772 | 0.603952 | | **0.028155** | |  | | St Dev | | 0.049361 | | 0.512369 | 0.009489 | | 0.574571 | **0.028155** | |  |
| Minimum | | 1.379749 | | 2.181915 | | 1.322219 | 2.268217 | | 1.869232 | |  | | Minimum | | 1.380211 | | 2.176091 | 1.322219 | | 2.306419 | 1.869232 | |  |
| Maximum | | 1.690196 | | 2.481443 | | 1.591065 | 2.494155 | | 1.924279 | |  | | Maximum | | 1.732191 | | 2.477121 | 1.567984 | | 2.494155 | 1.924279 | |  |
| 25% Quantile | | 1.482836 | | 2.235987 | | 1.413685 | 2.325883 | | 1.877861 | |  | | 25% Quantile | | 1.489318 | | 2.241571 | 1.390684 | | 2.342096 | 1.877861 | |  |
| 75% Quantile | | 1.623249 | | 2.406334 | | 1.554789 | 2.454453 | | 1.905385 | |  | | 75% Quantile | | 1.671127 | | 2.406334 | 1.52538 | | 2.459629 | 1.905385 | |  |
| Kurtosis | | -2.33333 | | -2.33333 | | -2.33333 | -2.33333 | | -2.333333 | |  | | Kurtosis | | -2.33333 | | -2.33333 | -2.33333 | | -2.33333 | -2.33333 | |  |
| Skew | | -0.38371 | | 0.383363 | | -0.3849 | 0.3849 | | 0.228698 | |  | | Skew | | -0.3839 | | 0.383847 | -0.3849 | | 0.3849 | 0.228698 | |  |
| IQR | | 0.05617 | | 0.5 | | 0.018256 | 0.569998 | | **0.027524** | |  | | IQR | | 0.045738 | | 0.474448 | 0.009051 | | 0.537676 | **0.027524** | |  |
|  | |  | |  | |  |  | |  | |  | |  | |  | |  |  | |  |  | |  |
| Mikrogeophagus-GGD | | | | | |  |  | |  | |  | | Mikrogeophagus-Geophagini | | | | |  | |  |  | |  |
|  | | 2.50% | | 97.50% | | 0.25% | 99.75% | | Observed | |  | |  | | 2.50% | | 97.50% | 0.25% | | 99.75% | Observed | |  |
| Mean | | 1.763069 | | 2.324424 | | 1.633826 | 2.382604 | | **1.639834** | |  | | Mean | | 1.553362 | | 2.347477 | 1.451001 | | 2.438443 | 1.639834 | |  |
| St Dev | | 0.0083 | | 0.511799 | | 0 | 0.582592 | | 0.153237 | |  | | St Dev | | 0.009536 | | 0.626613 | 0 | | 0.718854 | 0.153237 | |  |
| Minimum | | 1.531479 | | 2.296665 | | 1.531479 | 2.338302 | | **1.531479** | |  | | Minimum | | 1.380211 | | 2.296774 | 1.322219 | | 2.40654 | 1.531479 | |  |
| Maximum | | 1.880075 | | 2.447158 | | 1.736172 | 2.447158 | | **1.748188** | |  | | Maximum | | 1.591065 | | 2.462766 | 1.524964 | | 2.494155 | 1.748188 | |  |
| 25% Quantile | | 1.661801 | | 2.30357 | | 1.582652 | 2.357106 | | **1.585656** | |  | | 25% Quantile | | 1.506319 | | 2.314419 | 1.388013 | | 2.41776 | 1.585656 | |  |
| 75% Quantile | | 1.832794 | | 2.355164 | | 1.684999 | 2.414881 | | **1.694011** | |  | | 75% Quantile | | 1.572875 | | 2.398607 | 1.503548 | | 2.459582 | 1.694011 | |  |
| Kurtosis | | -2.75 | | -2.75 | | -2.75 | 0 | | -2.75 | |  | | Kurtosis | | -2.75 | | -2.75 | -2.75 | | 0 | -2.75 | |  |
| Skew | | -6.4E-15 | | 7.86E-15 | | -3.7E-14 | 3.16E-14 | | 0 | |  | | Skew | | -7.4E-15 | | 5.09E-15 | -2.7E-14 | | 6.64E-14 | 0 | |  |
| IQR | | 0.005869 | | 0.361897 | | 0 | 0.411954 | | 0.108355 | |  | | IQR | | 0.006743 | | 0.443082 | 0 | | 0.508306 | 0.108355 | |  |
|  | |  | |  | |  |  | |  | |  | |  | |  | |  |  | |  |  | |  |
| Satanoperca-CAS | | | |  | |  |  | |  | |  | | Satanoperca-Geophagini | | | | |  | |  |  | |  |
|  | | 2.50% | | 97.50% | | 0.25% | 99.75% | | Observed | |  | |  | | 2.50% | | 97.50% | 0.25% | | 99.75% | Observed | |  |
| Mean | | 1.726731 | | 2.163383 | | 1.623368 | 2.25888 | | **2.253459** | |  | | Mean | | 1.765266 | | 2.175694 | 1.68695 | | 2.260689 | **2.253459** | |  |
| St Dev | | 0.186098 | | 0.426208 | | 0.135057 | 0.450839 | | **0.077492** | |  | | St Dev | | 0.180494 | | 0.411719 | 0.110792 | | 0.439533 | **0.077492** | |  |
| Minimum | | 1.322219 | | 1.756249 | | 1.322219 | 1.995635 | | **2.149219** | |  | | Minimum | | 1.361728 | | 1.851853 | 1.322219 | | 2.002086 | **2.149219** | |  |
| Maximum | | 2.136641 | | 2.494155 | | 1.929419 | 2.494155 | | 2.40654 | |  | | Maximum | | 2.164353 | | 2.494155 | 2.058792 | | 2.494155 | 2.40654 | |  |
| 25% Quantile | | 1.500675 | | 2.086212 | | 1.4331 | 2.169293 | | **2.212163** | |  | | 25% Quantile | | 1.528997 | | 2.102612 | 1.47445 | | 2.185702 | **2.212163** | |  |
| 75% Quantile | | 1.803694 | | 2.404448 | | 1.674225 | 2.44006 | | 2.284864 | |  | | 75% Quantile | | 1.876135 | | 2.383419 | 1.739086 | | 2.429875 | 2.284864 | |  |
| Kurtosis | | -2.05676 | | -0.26679 | | -2.1547 | 0.619421 | | -0.635185 | |  | | Kurtosis | | -2.05212 | | -0.22929 | -2.15217 | | 0.512324 | -0.63518 | |  |
| Skew | | -0.8704 | | 0.912207 | | -1.32902 | 1.341956 | | 0.645183 | |  | | Skew | | -0.94799 | | 0.746938 | -1.33088 | | 1.175175 | 0.645183 | |  |
| IQR | | 0.171217 | | 0.755728 | | 0.110394 | 0.844238 | | **0.072701** | |  | | IQR | | 0.172001 | | 0.717099 | 0.116646 | | 0.811936 | **0.072701** | |  |
|  | |  | |  | |  |  | |  | |  | |  | |  | |  |  | |  |  | |  |
| Acaronia-Cichlasomatini | | | | | |  |  | |  | |  | |  | |  | |  |  | |  |  | |  |
|  | | 2.50% | | 97.50% | | 0.25% | 99.75% | | Observed | |  | |  | |  | |  |  | |  |  | |  |
| Mean | | 1.752676 | | 2.171665 | | 1.650279 | 2.208833 | | 2.13694 | |  | |  | |  | |  |  | |  |  | |  |
| St Dev | | 0.004945 | | 0.341074 | | 0 | 0.415913 | | 0.071532 | |  | |  | |  | |  |  | |  |  | |  |
| Minimum | | 1.653213 | | 2.10721 | | 1.556303 | 2.176091 | | 2.08636 | |  | |  | |  | |  |  | |  |  | |  |
| Maximum | | 1.838849 | | 2.30103 | | 1.694561 | 2.30103 | | 2.187521 | |  | |  | |  | |  |  | |  |  | |  |
| 25% Quantile | | 1.691388 | | 2.142329 | | 1.603291 | 2.19176 | | 2.11165 | |  | |  | |  | |  |  | |  |  | |  |
| 75% Quantile | | 1.803888 | | 2.204636 | | 1.688815 | 2.252575 | | 2.16223 | |  | |  | |  | |  |  | |  |  | |  |
| Kurtosis | | -2.75 | | -2.75 | | -2.75 | 0 | | -2.75 | |  | |  | |  | |  |  | |  |  | |  |
| Skew | | -1.3E-14 | | 8.64E-15 | | -6.4E-14 | 5.86E-14 | | 4.63E-15 | |  | |  | |  | |  |  | |  |  | |  |
| IQR | | 0.003497 | | 0.241176 | | 0 | 0.294095 | | 0.05058 | |  | |  | |  | |  |  | |  |  | |  |
|  | |  | |  | |  |  | |  | |  | |  | |  | |  |  | |  |  | |  |
| Aequidens-Cichlasomatini | | | | | |  |  | |  | |  | |  | |  | |  |  | |  |  | |  |
|  | | 2.50% | | 97.50% | | 0.25% | 99.75% | | Observed | |  | |  | |  | |  |  | |  |  | |  |
| Mean | | 1.923955 | | 2.030894 | | 1.904149 | 2.055559 | | **2.072709** | |  | |  | |  | |  |  | |  |  | |  |
| St Dev | | 0.113229 | | 0.187953 | | 0.100921 | 0.195895 | | 0.122879 | |  | |  | |  | |  |  | |  |  | |  |
| Minimum | | 1.556303 | | 1.792392 | | 1.556303 | 1.851258 | | 1.748188 | |  | |  | |  | |  |  | |  |  | |  |
| Maximum | | 2.127105 | | 2.30103 | | 2.089905 | 2.30103 | | 2.30103 | |  | |  | |  | |  |  | |  |  | |  |
| 25% Quantile | | 1.800919 | | 1.983293 | | 1.773956 | 2.006451 | | **2.0086** | |  | |  | |  | |  |  | |  |  | |  |
| 75% Quantile | | 2.025247 | | 2.122131 | | 2.001564 | 2.149087 | | **2.155336** | |  | |  | |  | |  |  | |  |  | |  |
| Kurtosis | | -1.29982 | | 1.125295 | | -1.4697 | 2.204246 | | 0.417369 | |  | |  | |  | |  |  | |  |  | |  |
| Skew | | -1.06452 | | 0.213842 | | -1.36084 | 0.500768 | | -0.477402 | |  | |  | |  | |  |  | |  |  | |  |
| IQR | | 0.111319 | | 0.26649 | | 0.087763 | 0.314404 | | 0.146736 | |  | |  | |  | |  |  | |  |  | |  |
|  | |  | |  | |  |  | |  | |  | |  | |  | |  |  | |  |  | |  |
| Andinoacara-Cichlasomatini | | | | | |  |  | |  | |  | |  | |  | |  |  | |  |  | |  |
|  | | 2.50% | | 97.50% | | 0.25% | 99.75% | | Observed | |  | |  | |  | |  |  | |  |  | |  |
| Mean | | 1.752676 | | 2.171665 | | 1.650279 | 2.208833 | | 2.026539 | |  | |  | |  | |  |  | |  |  | |  |
| St Dev | | 0.004945 | | 0.341074 | | 0 | 0.415913 | | 0.037532 | |  | |  | |  | |  |  | |  |  | |  |
| Minimum | | 1.653213 | | 2.10721 | | 1.556303 | 2.176091 | | 2 | |  | |  | |  | |  |  | |  |  | |  |
| Maximum | | 1.838849 | | 2.30103 | | 1.694561 | 2.30103 | | 2.053078 | |  | |  | |  | |  |  | |  |  | |  |
| 25% Quantile | | 1.691388 | | 2.142329 | | 1.603291 | 2.19176 | | 2.01327 | |  | |  | |  | |  |  | |  |  | |  |
| 75% Quantile | | 1.803888 | | 2.204636 | | 1.688815 | 2.252575 | | 2.039809 | |  | |  | |  | |  |  | |  |  | |  |
| Kurtosis | | -2.75 | | -2.75 | | -2.75 | 0 | | -2.75 | |  | |  | |  | |  |  | |  |  | |  |
| Skew | | -1.3E-14 | | 8.64E-15 | | -6.4E-14 | 5.86E-14 | | -8.91E-15 | |  | |  | |  | |  |  | |  |  | |  |
| IQR | | 0.003497 | | 0.241176 | | 0 | 0.294095 | | 0.026539 | |  | |  | |  | |  |  | |  |  | |  |
|  | |  | |  | |  |  | |  | |  | |  | |  | |  |  | |  |  | |  |
| Burjurquina-Cichlasomatini | | | | | |  |  | |  | |  | |  | |  | |  |  | |  |  | |  |
|  | | 2.50% | | 97.50% | | 0.25% | 99.75% | | Observed | |  | |  | |  | |  |  | |  |  | |  |
| Mean | | 1.911454 | | 2.036829 | | 1.890092 | 2.065585 | | 1.937795 | |  | |  | |  | |  |  | |  |  | |  |
| St Dev | | 0.104791 | | 0.195492 | | 0.091957 | 0.207243 | | 0.107573 | |  | |  | |  | |  |  | |  |  | |  |
| Minimum | | 1.556303 | | 1.826075 | | 1.556303 | 1.86029 | | 1.740363 | |  | |  | |  | |  |  | |  |  | |  |
| Maximum | | 2.10721 | | 2.30103 | | 2.082753 | 2.30103 | | 2.176091 | |  | |  | |  | |  |  | |  |  | |  |
| 25% Quantile | | 1.792036 | | 2 | | 1.748188 | 2.012837 | | 1.869232 | |  | |  | |  | |  |  | |  |  | |  |
| 75% Quantile | | 2.012837 | | 2.133539 | | 2 | 2.168766 | | 2.012837 | |  | |  | |  | |  |  | |  |  | |  |
| Kurtosis | | -1.40908 | | 1.205187 | | -1.58361 | 2.595683 | | -0.448003 | |  | |  | |  | |  |  | |  |  | |  |
| Skew | | -1.18571 | | 0.352912 | | -1.64337 | 0.647219 | | 0.191452 | |  | |  | |  | |  |  | |  |  | |  |
| IQR | | 0.09293 | | 0.278813 | | 0.067344 | 0.329059 | | 0.143606 | |  | |  | |  | |  |  | |  |  | |  |
|  | |  | |  | |  |  | |  | |  | |  | |  | |  |  | |  |  | |  |
| Cichlasoma-Cichlasomatini | | | | | |  |  | |  | |  | |  | |  | |  |  | |  |  | |  |
|  | | 2.50% | | 97.50% | | 0.25% | 99.75% | | Observed | |  | |  | |  | |  |  | |  |  | |  |
| Mean | | 1.896637 | | 2.047312 | | 1.868355 | 2.070787 | | 2.006583 | |  | |  | |  | |  |  | |  |  | |  |
| St Dev | | 0.094596 | | 0.208223 | | 0.072048 | 0.217897 | | 0.09514 | |  | |  | |  | |  |  | |  |  | |  |
| Minimum | | 1.556303 | | 1.869232 | | 1.556303 | 1.916459 | | 1.819544 | |  | |  | |  | |  |  | |  |  | |  |
| Maximum | | 2.089905 | | 2.30103 | | 2.053078 | 2.30103 | | 2.133539 | |  | |  | |  | |  |  | |  |  | |  |
| 25% Quantile | | 1.748188 | | 2.000215 | | 1.69897 | 2.03942 | | 1.963788 | |  | |  | |  | |  |  | |  |  | |  |
| 75% Quantile | | 2 | | 2.155336 | | 1.916433 | 2.168766 | | 2.068186 | |  | |  | |  | |  |  | |  |  | |  |
| Kurtosis | | -1.62582 | | 1.110222 | | -1.7777 | 2.24177 | | -0.97526 | |  | |  | |  | |  |  | |  |  | |  |
| Skew | | -1.21486 | | 0.442417 | | -1.65923 | 0.964734 | | -0.577073 | |  | |  | |  | |  |  | |  |  | |  |
| IQR | | 0.077826 | | 0.314818 | | 0.054208 | 0.366171 | | 0.104398 | |  | |  | |  | |  |  | |  |  | |  |
|  | |  | |  | |  |  | |  | |  | |  | |  | |  |  | |  |  | |  |
| Krobia-Cichlasomatini | | | | | |  |  | |  | |  | |  | |  | |  |  | |  |  | |  |
|  | | 2.50% | | 97.50% | | 0.25% | 99.75% | | Observed | |  | |  | |  | |  |  | |  |  | |  |
| Mean | | 1.796891 | | 2.132364 | | 1.708651 | 2.184014 | | 2.103777 | |  | |  | |  | |  |  | |  |  | |  |
| St Dev | | 0.027302 | | 0.286155 | | 0.009419 | 0.346488 | | **0.005947** | |  | |  | |  | |  |  | |  |  | |  |
| Minimum | | 1.556303 | | 2.064458 | | 1.556303 | 2.10721 | | **2.09691** | |  | |  | |  | |  |  | |  |  | |  |
| Maximum | | 1.913814 | | 2.30103 | | 1.826075 | 2.30103 | | 2.10721 | |  | |  | |  | |  |  | |  |  | |  |
| 25% Quantile | | 1.705431 | | 2.096413 | | 1.623249 | 2.153437 | | **2.10206** | |  | |  | |  | |  |  | |  |  | |  |
| 75% Quantile | | 1.870944 | | 2.19593 | | 1.763721 | 2.252218 | | 2.10721 | |  | |  | |  | |  |  | |  |  | |  |
| Kurtosis | | -2.33333 | | -2.33333 | | -2.33333 | -2.33333 | | -2.333333 | |  | |  | |  | |  |  | |  |  | |  |
| Skew | | -0.38406 | | 0.382084 | | -0.3849 | 0.3849 | | **-0.3849** | |  | |  | |  | |  |  | |  |  | |  |
| IQR | | 0.027053 | | 0.275454 | | 0.008966 | 0.32289 | | **0.00515** | |  | |  | |  | |  |  | |  |  | |  |
|  | |  | |  | |  |  | |  | |  | |  | |  | |  |  | |  |  | |  |
| Laetacara-Cichlasomatini | | | | | |  |  | |  | |  | |  | |  | |  |  | |  |  | |  |
|  | | 2.50% | | 97.50% | | 0.25% | 99.75% | | Observed | |  | |  | |  | |  |  | |  |  | |  |
| Mean | | 1.85402 | | 2.080199 | | 1.814803 | 2.120255 | | **1.769851** | |  | |  | |  | |  |  | |  |  | |  |
| St Dev | | 0.063498 | | 0.23632 | | 0.037533 | 0.276466 | | 0.136017 | |  | |  | |  | |  |  | |  |  | |  |
| Minimum | | 1.556303 | | 1.963788 | | 1.556303 | 2.025193 | | 1.556303 | |  | |  | |  | |  |  | |  |  | |  |
| Maximum | | 2.029384 | | 2.30103 | | 1.993353 | 2.30103 | | **1.913814** | |  | |  | |  | |  |  | |  |  | |  |
| 25% Quantile | | 1.730428 | | 2.044334 | | 1.674371 | 2.072143 | | **1.691606** | |  | |  | |  | |  |  | |  |  | |  |
| 75% Quantile | | 1.948602 | | 2.175107 | | 1.882828 | 2.201555 | | **1.861636** | |  | |  | |  | |  |  | |  |  | |  |
| Kurtosis | | -2.08837 | | -0.54478 | | -2.21358 | -0.28393 | | -1.655603 | |  | |  | |  | |  |  | |  |  | |  |
| Skew | | -1.04842 | | 0.701522 | | -1.24131 | 0.956815 | | -0.441928 | |  | |  | |  | |  |  | |  |  | |  |
| IQR | | 0.043909 | | 0.33466 | | 0.023329 | 0.404295 | | 0.17003 | |  | |  | |  | |  |  | |  |  | |  |
|  | |  | |  | |  |  | |  | |  | |  | |  | |  |  | |  |  | |  |
| Nannacara-Cichlasomatini | | | | | |  |  | |  | |  | |  | |  | |  |  | |  |  | |  |
|  | | 2.50% | | 97.50% | | 0.25% | 99.75% | | Observed | |  | |  | |  | |  |  | |  |  | |  |
| Mean | | 1.85402 | | 2.080199 | | 1.814803 | 2.120255 | | **1.723328** | |  | |  | |  | |  |  | |  |  | |  |
| St Dev | | 0.063498 | | 0.23632 | | 0.037533 | 0.276466 | | 0.066675 | |  | |  | |  | |  |  | |  |  | |  |
| Minimum | | 1.556303 | | 1.963788 | | 1.556303 | 2.025193 | | 1.653213 | |  | |  | |  | |  |  | |  |  | |  |
| Maximum | | 2.029384 | | 2.30103 | | 1.993353 | 2.30103 | | **1.826075** | |  | |  | |  | |  |  | |  |  | |  |
| 25% Quantile | | 1.730428 | | 2.044334 | | 1.674371 | 2.072143 | | **1.690196** | |  | |  | |  | |  |  | |  |  | |  |
| 75% Quantile | | 1.948602 | | 2.175107 | | 1.882828 | 2.201555 | | **1.748188** | |  | |  | |  | |  |  | |  |  | |  |
| Kurtosis | | -2.08837 | | -0.54478 | | -2.21358 | -0.28393 | | -1.607937 | |  | |  | |  | |  |  | |  |  | |  |
| Skew | | -1.04842 | | 0.701522 | | -1.24131 | 0.956815 | | 0.475373 | |  | |  | |  | |  |  | |  |  | |  |
| IQR | | 0.043909 | | 0.33466 | | 0.023329 | 0.404295 | | 0.057992 | |  | |  | |  | |  |  | |  |  | |  |
|  | |  | |  | |  |  | |  | |  | |  | |  | |  |  | |  |  | |  |
| Tahuantinsuyoa-Cichlasomatini | | | | | | |  | |  | |  | |  | |  | |  |  | |  |  | |  |
|  | | 2.50% | | 97.50% | | 0.25% | 99.75% | | Observed | |  | |  | |  | |  |  | |  |  | |  |
| Mean | | 1.752676 | | 2.171665 | | 1.650279 | 2.208833 | | 1.996498 | |  | |  | |  | |  |  | |  |  | |  |
| St Dev | | 0.004945 | | 0.341074 | | 0 | 0.415913 | | 0.116932 | |  | |  | |  | |  |  | |  |  | |  |
| Minimum | | 1.653213 | | 2.10721 | | 1.556303 | 2.176091 | | 1.913814 | |  | |  | |  | |  |  | |  |  | |  |
| Maximum | | 1.838849 | | 2.30103 | | 1.694561 | 2.30103 | | 2.079181 | |  | |  | |  | |  |  | |  |  | |  |
| 25% Quantile | | 1.691388 | | 2.142329 | | 1.603291 | 2.19176 | | 1.955156 | |  | |  | |  | |  |  | |  |  | |  |
| 75% Quantile | | 1.803888 | | 2.204636 | | 1.688815 | 2.252575 | | 2.037839 | |  | |  | |  | |  |  | |  |  | |  |
| Kurtosis | | -2.75 | | -2.75 | | -2.75 | 0 | | -2.75 | |  | |  | |  | |  |  | |  |  | |  |
| Skew | | -1.3E-14 | | 8.64E-15 | | -6.4E-14 | 5.86E-14 | | 0 | |  | |  | |  | |  |  | |  |  | |  |
| IQR | | 0.003497 | | 0.241176 | | 0 | 0.294095 | | 0.082684 | |  | |  | |  | |  |  | |  |  | |  |
|  | |  | |  | |  |  | |  | |  | |  | |  | |  |  | |  |  | |  |
| Amatitlania-SAClade | | | | | |  |  | |  | |  | | Amatitilania-Heroini | | | | |  | |  |  | |  |
|  | | 2.50% | | 97.50% | | 0.25% | 99.75% | | Observed | |  | |  | | 2.50% | | 97.50% | 0.25% | | 99.75% | Observed | |  |
| Mean | | 1.985239 | | 2.390636 | | 1.926607 | 2.460198 | | **1.943937** | |  | | Mean | | 2.000117 | | 2.374588 | 1.94 | | 2.439884 | **1.943937** | |  |
| St Dev | | 0.064887 | | 0.344814 | | 0.037344 | 0.393993 | | **0.045217** | |  | | St Dev | | 0.056272 | | 0.359428 | 0.033396 | | 0.431096 | **0.045217** | |  |
| Minimum | | 1.755875 | | 2.230449 | | 1.724276 | 2.316813 | | 1.897627 | |  | | Minimum | | 1.724276 | | 2.225309 | 1.69897 | | 2.366421 | 1.897627 | |  |
| Maximum | | 2.133459 | | 2.69897 | | 2.041393 | 2.69897 | | **2** | |  | | Maximum | | 2.133539 | | 2.69897 | 2.041393 | | 2.69897 | **2** | |  |
| 25% Quantile | | 1.863173 | | 2.332312 | | 1.814893 | 2.382232 | | 1.913715 | |  | | 25% Quantile | | 1.863121 | | 2.326967 | 1.829216 | | 2.391209 | 1.913715 | |  |
| 75% Quantile | | 2.036532 | | 2.508356 | | 1.963254 | 2.564683 | | **1.969281** | |  | | 75% Quantile | | 2.047103 | | 2.48617 | 1.969908 | | 2.582794 | **1.969281** | |  |
| Kurtosis | | -2.40435 | | -1.69922 | | -2.43479 | -1.68838 | | -2.108198 | |  | | Kurtosis | | -2.40558 | | -1.70607 | -2.43182 | | -1.68884 | -2.1082 | |  |
| Skew | | -0.71678 | | 0.68431 | | -0.74843 | 0.742409 | | 0.175724 | |  | | Skew | | -0.70341 | | 0.685824 | -0.74753 | | 0.7448 | 0.175724 | |  |
| IQR | | 0.053685 | | 0.459002 | | 0.029608 | 0.608406 | | 0.055566 | |  | | IQR | | 0.05587 | | 0.475769 | 0.021835 | | 0.627615 | **0.055566** | |  |
|  | |  | |  | |  |  | |  | |  | |  | |  | |  |  | |  |  | |  |
| Amphilophus-CAClade | | | | | |  |  | |  | |  | | Amphilophus-Heroini | | | | |  | |  |  | |  |
|  | | 2.50% | | 97.50% | | 0.25% | 99.75% | | Observed | |  | |  | | 2.50% | | 97.50% | 0.25% | | 99.75% | Observed | |  |
| Mean | | 2.110582 | | 2.273473 | | 2.087214 | 2.303713 | | 2.221018 | |  | | Mean | | 2.091715 | | 2.261256 | 2.062755 | | 2.287468 | 2.221018 | |  |
| St Dev | | 0.155222 | | 0.263522 | | 0.125494 | 0.281561 | | **0.095584** | |  | | St Dev | | 0.151656 | | 0.261193 | 0.134141 | | 0.277679 | **0.095584** | |  |
| Minimum | | 1.724276 | | 1.954243 | | 1.724276 | 2.041393 | | **2.103804** | |  | | Minimum | | 1.69897 | | 1.94939 | 1.69897 | | 2 | **2.103804** | |  |
| Maximum | | 2.39794 | | 2.69897 | | 2.380211 | 2.69897 | | 2.39794 | |  | | Maximum | | 2.39794 | | 2.69897 | 2.34701 | | 2.69897 | 2.39794 | |  |
| 25% Quantile | | 1.915047 | | 2.168601 | | 1.890694 | 2.207682 | | **2.170141** | |  | | 25% Quantile | | 1.909767 | | 2.157612 | 1.892052 | | 2.189202 | 2.170141 | |  |
| 75% Quantile | | 2.220725 | | 2.416721 | | 2.173806 | 2.465941 | | 2.244239 | |  | | 75% Quantile | | 2.204636 | | 2.410245 | 2.153486 | | 2.465941 | 2.244239 | |  |
| Kurtosis | | -1.53317 | | 0.256597 | | -1.65244 | 0.999006 | | -0.832532 | |  | | Kurtosis | | -1.53637 | | 0.121597 | -1.68506 | | 0.920888 | -0.83253 | |  |
| Skew | | -0.72744 | | 0.690251 | | -1.04909 | 0.984483 | | **0.801347** | |  | | Skew | | -0.68612 | | 0.668368 | -0.926 | | 1.042156 | **0.801347** | |  |
| IQR | | 0.166677 | | 0.431478 | | 0.115542 | 0.481553 | | **0.074099** | |  | | IQR | | 0.15715 | | 0.432797 | 0.131555 | | 0.488499 | **0.074099** | |  |
|  | |  | |  | |  |  | |  | |  | |  | |  | |  |  | |  |  | |  |
| Archocentrus-CAClade | | | | | |  |  | |  | |  | | Archocentrus-Heroini | | | | |  | |  |  | |  |
|  | | 2.50% | | 97.50% | | 0.25% | 99.75% | | Observed | |  | |  | | 2.50% | | 97.50% | 0.25% | | 99.75% | Observed | |  |
| Mean | | 1.905586 | | 2.462361 | | 1.826407 | 2.604866 | | 2.041393 | |  | | Mean | | 1.899405 | | 2.437531 | 1.792532 | | 2.548455 | 2.041393 | |  |
| St Dev | | 0 | | 0.430058 | | 0 | 0.595289 | | 0 | |  | | St Dev | | 0.004874 | | 0.476353 | 0 | | 0.598503 | **0** | |  |
| Minimum | | 1.78533 | | 2.39794 | | 1.724276 | 2.510762 | | 2.041393 | |  | | Minimum | | 1.724276 | | 2.39794 | 1.69897 | | 2.414973 | 2.041393 | |  |
| Maximum | | 1.954121 | | 2.677251 | | 1.874994 | 2.69897 | | 2.041393 | |  | | Maximum | | 1.959041 | | 2.676694 | 1.854165 | | 2.69897 | 2.041393 | |  |
| 25% Quantile | | 1.862895 | | 2.417897 | | 1.784116 | 2.557814 | | NA | |  | | 25% Quantile | | 1.840977 | | 2.417735 | 1.747915 | | 2.473198 | NA | |  |
| 75% Quantile | | 1.923111 | | 2.558432 | | 1.856761 | 2.651918 | | NA | |  | | 75% Quantile | | 1.918815 | | 2.516791 | 1.832741 | | 2.623713 | NA | |  |
| Kurtosis | | -2.75 | | 0 | | -2.75 | 0 | | NA | |  | | Kurtosis | | -2.75 | | -2.75 | -2.75 | | 0 | NA | |  |
| Skew | | -8.8E-15 | | 8.75E-15 | | -3.6E-14 | 5.05E-14 | | NA | |  | | Skew | | -8.7E-15 | | 8.64E-15 | -9.5E-14 | | 4.5E-14 | NA | |  |
| IQR | | 0 | | 0.304097 | | 0 | 0.420933 | | 0 | |  | | IQR | | 0.003447 | | 0.336832 | 0 | | 0.423206 | **0** | |  |
|  | |  | |  | |  |  | |  | |  | |  | |  | |  |  | |  |  | |  |
| Astatheros-CAClade | | | | | |  |  | |  | |  | | Astatheros-Heroini | | | |  |  | |  |  | |  |
|  | | 2.50% | | 97.50% | | 0.25% | 99.75% | | Observed | |  | |  | | 2.50% | | 97.50% | 0.25% | | 99.75% | Observed | |  |
| Mean | | 2.053328 | | 2.32579 | | 1.99612 | 2.370788 | | 2.17873 | |  | | Mean | | 2.041345 | | 2.319021 | 1.987705 | | 2.377717 | 2.17873 | |  |
| St Dev | | 0.113155 | | 0.302369 | | 0.089192 | 0.351671 | | 0.195858 | |  | | St Dev | | 0.1078 | | 0.305633 | 0.078219 | | 0.346207 | 0.195858 | |  |
| Minimum | | 1.724276 | | 2.113943 | | 1.724276 | 2.20412 | | 1.845098 | |  | | Minimum | | 1.69897 | | 2.120737 | 1.69897 | | 2.203976 | 1.845098 | |  |
| Maximum | | 2.278754 | | 2.69897 | | 2.190036 | 2.69897 | | 2.39794 | |  | | Maximum | | 2.269513 | | 2.69897 | 2.197191 | | 2.69897 | 2.39794 | |  |
| 25% Quantile | | 1.877899 | | 2.253088 | | 1.848412 | 2.295484 | | 2.093318 | |  | | 25% Quantile | | 1.877669 | | 2.25313 | 1.834507 | | 2.341295 | 2.093318 | |  |
| 75% Quantile | | 2.146108 | | 2.477121 | | 2.087012 | 2.552508 | | 2.30855 | |  | | 75% Quantile | | 2.115749 | | 2.442803 | 2.05007 | | 2.501143 | 2.30855 | |  |
| Kurtosis | | -1.97395 | | -0.19839 | | -2.14107 | 0.372635 | | -1.414055 | |  | | Kurtosis | | -1.97831 | | 0.020259 | -2.07746 | | 0.949426 | -1.41405 | |  |
| Skew | | -0.86061 | | 0.844329 | | -1.28999 | 1.192641 | | -0.36912 | |  | | Skew | | -0.87935 | | 1.0372 | -1.51525 | | 1.405416 | -0.36912 | |  |
| IQR | | 0.092196 | | 0.484397 | | 0.059478 | 0.558406 | | 0.215232 | |  | | IQR | | 0.095158 | | 0.457009 | 0.055637 | | 0.566928 | 0.215232 | |  |
|  | |  | |  | |  |  | |  | |  | |  | |  | |  |  | |  |  | |  |
| Australoheros-CAClade | | | | | |  |  | |  | |  | | Australoheros-Heroini | | | | |  | |  |  | |  |
|  | | 2.50% | | 97.50% | | 0.25% | 99.75% | | Observed | |  | |  | | 2.50% | | 97.50% | 0.25% | | 99.75% | Observed | |  |
| Mean | | 2.12095 | | 2.262014 | | 2.094227 | 2.284151 | | **1.969426** | |  | | Mean | | 2.10742 | | 2.243552 | 2.061168 | | 2.280632 | **1.969426** | |  |
| St Dev | | 0.166777 | | 0.249765 | | 0.15333 | 0.264062 | | **0.138089** | |  | | St Dev | | 0.16232 | | 0.253262 | 0.142787 | | 0.269983 | **0.138089** | |  |
| Minimum | | 1.724276 | | 1.90309 | | 1.724276 | 1.956654 | | 1.724276 | |  | | Minimum | | 1.69897 | | 1.90309 | 1.69897 | | 1.982271 | 1.724276 | |  |
| Maximum | | 2.414973 | | 2.69897 | | 2.39794 | 2.69897 | | **2.285557** | |  | | Maximum | | 2.39794 | | 2.69897 | 2.39794 | | 2.69897 | **2.285557** | |  |
| 25% Quantile | | 1.939261 | | 2.147497 | | 1.901717 | 2.19681 | | **1.883652** | |  | | 25% Quantile | | 1.916446 | | 2.131913 | 1.902936 | | 2.173713 | **1.883652** | |  |
| 75% Quantile | | 2.242861 | | 2.406457 | | 2.19688 | 2.437531 | | **2.082771** | |  | | 75% Quantile | | 2.226539 | | 2.39794 | 2.176091 | | 2.449897 | **2.082771** | |  |
| Kurtosis | | -1.45693 | | 0.124367 | | -1.66701 | 0.53429 | | -0.566511 | |  | | Kurtosis | | -1.42729 | | 0.104139 | -1.56288 | | 0.740818 | -0.56651 | |  |
| Skew | | -0.58533 | | 0.565577 | | -0.95538 | 0.777699 | | 0.389844 | |  | | Skew | | -0.57581 | | 0.553741 | -0.75448 | | 0.771117 | 0.389844 | |  |
| IQR | | 0.193766 | | 0.427441 | | 0.173307 | 0.466025 | | 0.199118 | |  | | IQR | | 0.187535 | | 0.418426 | 0.146503 | | 0.453403 | 0.199118 | |  |
|  | |  | |  | |  |  | |  | |  | |  | |  | |  |  | |  |  | |  |
| Caquetaia-CAClade | | | |  | |  |  | |  | |  | | Caquetaia-Heroini | | | |  |  | |  |  | |  |
|  | | 2.50% | | 97.50% | | 0.25% | 99.75% | | Observed | |  | |  | | 2.50% | | 97.50% | 0.25% | | 99.75% | Observed | |  |
| Mean | | 1.985239 | | 2.390636 | | 1.926607 | 2.460198 | | **2.396976** | |  | | Mean | | 1.983691 | | 2.382145 | 1.909385 | | 2.452125 | **2.396976** | |  |
| St Dev | | 0.064887 | | 0.344814 | | 0.037344 | 0.393993 | | 0.203928 | |  | | St Dev | | 0.059406 | | 0.355309 | 0.0256 | | 0.428401 | 0.203928 | |  |
| Minimum | | 1.755875 | | 2.230449 | | 1.724276 | 2.316813 | | 2.217484 | |  | | Minimum | | 1.69897 | | 2.225309 | 1.69897 | | 2.390913 | 2.217484 | |  |
| Maximum | | 2.133459 | | 2.69897 | | 2.041393 | 2.69897 | | 2.676694 | |  | | Maximum | | 2.136641 | | 2.69897 | 2.041393 | | 2.69897 | 2.676694 | |  |
| 25% Quantile | | 1.863173 | | 2.332312 | | 1.814893 | 2.382232 | | 2.263436 | |  | | 25% Quantile | | 1.862137 | | 2.319356 | 1.811344 | | 2.39794 | 2.263436 | |  |
| 75% Quantile | | 2.036532 | | 2.508356 | | 1.963254 | 2.564683 | | 2.480403 | |  | | 75% Quantile | | 2.036492 | | 2.498414 | 1.943631 | | 2.620718 | 2.480403 | |  |
| Kurtosis | | -2.40435 | | -1.69922 | | -2.43479 | -1.68838 | | -1.936768 | |  | | Kurtosis | | -2.4028 | | -1.70146 | -2.43197 | | -1.68798 | -1.93677 | |  |
| Skew | | -0.71678 | | 0.68431 | | -0.74843 | 0.742409 | | 0.426155 | |  | | Skew | | -0.70207 | | 0.6779 | -0.74906 | | 0.740554 | 0.426155 | |  |
| IQR | | 0.053685 | | 0.459002 | | 0.029608 | 0.608406 | | 0.216967 | |  | | IQR | | 0.047718 | | 0.47023 | 0.023595 | | 0.588879 | 0.216967 | |  |
|  | |  | |  | |  |  | |  | |  | |  | |  | |  |  | |  |  | |  |
| Cryptoheros-CAClade | | | | | |  |  | |  | |  | | Cryptoheros-Heroini | | | | |  | |  |  | |  |
|  | | 2.50% | | 97.50% | | 0.25% | 99.75% | | Observed | |  | |  | | 2.50% | | 97.50% | 0.25% | | 99.75% | Observed | |  |
| Mean | | 2.061296 | | 2.328774 | | 2.028444 | 2.388821 | | **1.977042** | |  | | Mean | | 2.047278 | | 2.311348 | 1.991944 | | 2.359206 | **1.977042** | |  |
| St Dev | | 0.122227 | | 0.294508 | | 0.096859 | 0.327959 | | **0.077931** | |  | | St Dev | | 0.119926 | | 0.294033 | 0.09066 | | 0.332712 | **0.077931** | |  |
| Minimum | | 1.724276 | | 2.100371 | | 1.724276 | 2.164249 | | 1.845098 | |  | | Minimum | | 1.69897 | | 2.08636 | 1.69897 | | 2.146128 | 1.845098 | |  |
| Maximum | | 2.30103 | | 2.69897 | | 2.244269 | 2.69897 | | **2.113943** | |  | | Maximum | | 2.30103 | | 2.69897 | 2.217484 | | 2.69897 | **2.113943** | |  |
| 25% Quantile | | 1.891955 | | 2.245757 | | 1.845098 | 2.337456 | | 1.954243 | |  | | 25% Quantile | | 1.880814 | | 2.2431 | 1.845098 | | 2.334748 | 1.954243 | |  |
| 75% Quantile | | 2.146128 | | 2.477121 | | 2.086266 | 2.562293 | | **2** | |  | | 75% Quantile | | 2.133539 | | 2.477121 | 2.027044 | | 2.477121 | **2** | |  |
| Kurtosis | | -1.93159 | | 0.008445 | | -2.04604 | 0.950479 | | -0.854437 | |  | | Kurtosis | | -1.90523 | | 0.0464 | -1.99882 | | 1.005139 | -0.85444 | |  |
| Skew | | -0.92163 | | 0.861735 | | -1.30442 | 1.242934 | | 0.054127 | |  | | Skew | | -0.84952 | | 0.88049 | -1.43572 | | 1.271883 | 0.054127 | |  |
| IQR | | 0.083521 | | 0.49485 | | 0.050865 | 0.548477 | | **0.045757** | |  | | IQR | | 0.084311 | | 0.490254 | 0.040961 | | 0.551923 | **0.045757** | |  |
|  | |  | |  | |  |  | |  | |  | |  | |  | |  |  | |  |  | |  |
| Herichthys-CAClade | | | |  | |  |  | |  | |  | | Herichthys-Heroini | | | |  |  | |  |  | |  |
|  | | 2.50% | | 97.50% | | 0.25% | 99.75% | | Observed | |  | |  | | 2.50% | | 97.50% | 0.25% | | 99.75% | Observed | |  |
| Mean | | 2.061296 | | 2.328774 | | 2.028444 | 2.388821 | | 2.295085 | |  | | Mean | | 2.047278 | | 2.311348 | 1.991944 | | 2.359206 | 2.295085 | |  |
| St Dev | | 0.122227 | | 0.294508 | | 0.096859 | 0.327959 | | 0.169858 | |  | | St Dev | | 0.119926 | | 0.294033 | 0.09066 | | 0.332712 | 0.169858 | |  |
| Minimum | | 1.724276 | | 2.100371 | | 1.724276 | 2.164249 | | 2.079181 | |  | | Minimum | | 1.69897 | | 2.08636 | 1.69897 | | 2.146128 | 2.079181 | |  |
| Maximum | | 2.30103 | | 2.69897 | | 2.244269 | 2.69897 | | 2.60206 | |  | | Maximum | | 2.30103 | | 2.69897 | 2.217484 | | 2.69897 | 2.60206 | |  |
| 25% Quantile | | 1.891955 | | 2.245757 | | 1.845098 | 2.337456 | | 2.243038 | |  | | 25% Quantile | | 1.880814 | | 2.2431 | 1.845098 | | 2.334748 | 2.243038 | |  |
| 75% Quantile | | 2.146128 | | 2.477121 | | 2.086266 | 2.562293 | | 2.39794 | |  | | 75% Quantile | | 2.133539 | | 2.477121 | 2.027044 | | 2.477121 | 2.39794 | |  |
| Kurtosis | | -1.93159 | | 0.008445 | | -2.04604 | 0.950479 | | -1.168849 | |  | | Kurtosis | | -1.90523 | | 0.0464 | -1.99882 | | 1.005139 | -1.16885 | |  |
| Skew | | -0.92163 | | 0.861735 | | -1.30442 | 1.242934 | | 0.412905 | |  | | Skew | | -0.84952 | | 0.88049 | -1.43572 | | 1.271883 | 0.412905 | |  |
| IQR | | 0.083521 | | 0.49485 | | 0.050865 | 0.548477 | | 0.154902 | |  | | IQR | | 0.084311 | | 0.490254 | 0.040961 | | 0.551923 | 0.154902 | |  |
|  | |  | |  | |  |  | |  | |  | |  | |  | |  |  | |  |  | |  |
| Heros-SAClade | | | |  | |  |  | |  | |  | | Heros-Heroini | | | |  |  | |  |  | |  |
|  | |  | |  | | 0.25% | 99.75% | | Observed | |  | |  | | 2.50% | | 97.50% | 0.25% | | 99.75% | Observed | |  |
| Mean | |  | |  | | 1.863967 | 2.298511 | | 2.175608 | |  | | Mean | | 1.983691 | | 2.382145 | 1.909385 | | 2.452125 | 2.175608 | |  |
| St Dev | |  | |  | | 0.040325 | 0.332051 | | 0.092912 | |  | | St Dev | | 0.059406 | | 0.355309 | 0.0256 | | 0.428401 | 0.092912 | |  |
| Minimum | | | |  | | 1.69897 | 2.20412 | | 2.079181 | |  | | Minimum | | 1.69897 | | 2.225309 | 1.69897 | | 2.390913 | 2.079181 | |  |
| Maximum | | | |  | | 1.973128 | 2.39794 | | 2.30103 | |  | | Maximum | | 2.136641 | | 2.69897 | 2.041393 | | 2.69897 | 2.30103 | |  |
| 25% Quantile | | | |  | | 1.813186 | 2.26497 | | 2.129391 | |  | | 25% Quantile | | 1.862137 | | 2.319356 | 1.811344 | | 2.39794 | 2.129391 | |  |
| 75% Quantile | | | |  | | 1.928642 | 2.377786 | | 2.207326 | |  | | 75% Quantile | | 2.036492 | | 2.498414 | 1.943631 | | 2.620718 | 2.207326 | |  |
| Kurtosis | |  | |  | | -2.43208 | -1.70205 | | -1.877314 | |  | | Kurtosis | | -2.4028 | | -1.70146 | -2.43197 | | -1.68798 | -1.87731 | |  |
| Skew | |  | |  | | -0.72742 | 0.715492 | | 0.32752 | |  | | Skew | | -0.70207 | | 0.6779 | -0.74906 | | 0.740554 | 0.32752 | |  |
| IQR | |  | |  | | 0.031259 | 0.473198 | | 0.077935 | |  | | IQR | | 0.047718 | | 0.47023 | 0.023595 | | 0.588879 | 0.077935 | |  |
|  | |  | |  | |  |  | |  | |  | |  | |  | |  |  | |  |  | |  |
| Mesonauta-SAClade | | | | | |  |  | |  | |  | | Mesonauta-Heroini | | | |  |  | |  |  | |  |
|  | | 2.50% | | 97.50% | | 0.25% | 99.75% | | Observed | |  | |  | | 2.50% | | 97.50% | 0.25% | | 99.75% | Observed | |  |
| Mean | | 1.970995 | | 2.206525 | | 1.933487 | 2.247687 | | **1.939798** | |  | | Mean | | 2.00971 | | 2.332167 | 1.934124 | | 2.381028 | **1.939798** | |  |
| St Dev | | 0.090477 | | 0.258448 | | 0.06948 | 0.278444 | | **0.0568** | |  | | St Dev | | 0.093917 | | 0.323935 | 0.053581 | | 0.36551 | **0.0568** | |  |
| Minimum | | 1.69897 | | 2 | | 1.69897 | 2.120574 | | 1.851258 | |  | | Minimum | | 1.69897 | | 2.146128 | 1.69897 | | 2.230449 | 1.851258 | |  |
| Maximum | | 2.146128 | | 2.39794 | | 2.099774 | 2.39794 | | **2** | |  | | Maximum | | 2.20412 | | 2.69897 | 2.103631 | | 2.69897 | **2** | |  |
| 25% Quantile | | 1.866897 | | 2.153619 | | 1.857209 | 2.189525 | | 1.913814 | |  | | 25% Quantile | | 1.877696 | | 2.272322 | 1.816104 | | 2.384643 | 1.913814 | |  |
| 75% Quantile | | 2.056079 | | 2.347989 | | 1.978453 | 2.353558 | | **1.983361** | |  | | 75% Quantile | | 2.081017 | | 2.457326 | 1.950954 | | 2.581032 | **1.983361** | |  |
| Kurtosis | | -2.09905 | | -0.75892 | | -2.21959 | -0.337 | | -1.693353 | |  | | Kurtosis | | -2.11182 | | -0.60774 | -2.2568 | | -0.24705 | -1.69335 | |  |
| Skew | | -0.84012 | | 0.680429 | | -1.19819 | 0.966148 | | -0.336777 | |  | | Skew | | -0.89142 | | 0.890352 | -1.1362 | | 1.172191 | -0.33678 | |  |
| IQR | | 0.068676 | | 0.368386 | | 0.04399 | 0.420506 | | 0.069547 | |  | | IQR | | 0.063096 | | 0.442611 | 0.028008 | | 0.545362 | 0.069547 | |  |
|  | |  | |  | |  |  | |  | |  | |  | |  | |  |  | |  |  | |  |
| Parachromis-CA Clade | | | | | |  |  | |  | |  | | Parachromis-Heroini | | | | |  | |  |  | |  |
|  | | 2.50% | | 97.50% | | 0.25% | 99.75% | | Observed | |  | |  | | 2.50% | | 97.50% | 0.25% | | 99.75% | Observed | |  |
| Mean | | 2.0164 | | 2.364696 | | 1.946853 | 2.425129 | | **2.446569** | |  | | Mean | | 2.008439 | | 2.360828 | 1.946012 | | 2.453369 | **2.446569** | |  |
| St Dev | | 0.07883 | | 0.320628 | | 0.043744 | 0.373226 | | 0.164066 | |  | | St Dev | | 0.082559 | | 0.335975 | 0.045862 | | 0.407895 | 0.164066 | |  |
| Minimum | | 1.724276 | | 2.176091 | | 1.724276 | 2.278754 | | **2.267172** | |  | | Minimum | | 1.69897 | | 2.190332 | 1.69897 | | 2.293332 | **2.267172** | |  |
| Maximum | | 2.198657 | | 2.69897 | | 2.096879 | 2.69897 | | 2.69897 | |  | | Maximum | | 2.176091 | | 2.69897 | 2.098414 | | 2.69897 | 2.69897 | |  |
| 25% Quantile | | 1.869084 | | 2.332439 | | 1.78533 | 2.39794 | | **2.342423** | |  | | 25% Quantile | | 1.851258 | | 2.30103 | 1.739996 | | 2.39794 | **2.342423** | |  |
| 75% Quantile | | 2.079181 | | 2.477121 | | 1.977677 | 2.60206 | | 2.477121 | |  | | 75% Quantile | | 2.041393 | | 2.477121 | 1.973128 | | 2.60206 | 2.477121 | |  |
| Kurtosis | | -2.21084 | | -1.07594 | | -2.24418 | -0.94225 | | -1.561098 | |  | | Kurtosis | | -2.22046 | | -1.06014 | -2.24231 | | -0.97369 | -1.5611 | |  |
| Skew | | -0.89354 | | 0.819236 | | -1.05565 | 0.990734 | | 0.416867 | |  | | Skew | | -0.87554 | | 0.864609 | -1.02437 | | 1.008546 | 0.416867 | |  |
| IQR | | 0.036895 | | 0.482584 | | 0.006804 | 0.585027 | | 0.134699 | |  | | IQR | | 0.033424 | | 0.48736 | 0.01252 | | 0.621169 | 0.134699 | |  |
|  | |  | |  | |  |  | |  | |  | |  | |  | |  |  | |  |  | |  |
| Paraneetroplus-CA Clade | | | | | |  |  | |  | |  | | Paraneetroplus-Heroini | | | | |  | |  |  | |  |
|  | | 2.50% | | 97.50% | | 0.25% | 99.75% | | Observed | |  | |  | | 2.50% | | 97.50% | 0.25% | | 99.75% | Observed | |  |
| Mean | | 2.09099 | | 2.297941 | | 2.056295 | 2.346228 | | **2.369704** | |  | | Mean | | 2.070329 | | 2.279336 | 2.015755 | | 2.316529 | **2.369704** | |  |
| St Dev | | 0.142756 | | 0.273733 | | 0.113375 | 0.295646 | | **0.110495** | |  | | St Dev | | 0.13823 | | 0.268433 | 0.111868 | | 0.29944 | **0.110495** | |  |
| Minimum | | 1.724276 | | 2 | | 1.724276 | 2.118889 | | **2.117271** | |  | | Minimum | | 1.69897 | | 1.982271 | 1.69897 | | 2.102049 | **2.117271** | |  |
| Maximum | | 2.39794 | | 2.69897 | | 2.30103 | 2.69897 | | 2.544068 | |  | | Maximum | | 2.371068 | | 2.69897 | 2.26298 | | 2.69897 | 2.544068 | |  |
| 25% Quantile | | 1.907087 | | 2.203413 | | 1.875636 | 2.254474 | | **2.316205** | |  | | 25% Quantile | | 1.903088 | | 2.183098 | 1.872111 | | 2.22465 | **2.316205** | |  |
| 75% Quantile | | 2.196085 | | 2.457326 | | 2.123758 | 2.477121 | | 2.425158 | |  | | 75% Quantile | | 2.168441 | | 2.423062 | 2.112134 | | 2.460535 | **2.425158** | |  |
| Kurtosis | | -1.70727 | | 0.228753 | | -1.846 | 1.092677 | | -0.223232 | |  | | Kurtosis | | -1.71072 | | 0.384977 | -1.8372 | | 0.996303 | -0.22323 | |  |
| Skew | | -0.80895 | | 0.758046 | | -1.06762 | 1.010063 | | -0.637566 | |  | | Skew | | -0.75194 | | 0.749755 | -1.0293 | | 1.147227 | -0.63757 | |  |
| IQR | | 0.141771 | | 0.451378 | | 0.086906 | 0.516702 | | **0.108953** | |  | | IQR | | 0.133716 | | 0.444315 | 0.107244 | | 0.508284 | **0.108953** | |  |
|  | |  | |  | |  |  | |  | |  | |  | |  | |  |  | |  |  | |  |
| Pterophyllum-SA Clade | | | | | |  |  | |  | |  | | Pterophyllum-Heroini | | | | |  | |  |  | |  |
|  | | 2.50% | | 97.50% | | 0.25% | 99.75% | | Observed | |  | |  | | 2.50% | | 97.50% | 0.25% | | 99.75% | Observed | |  |
| Mean | | 1.892962 | | 2.276864 | | 1.818804 | 2.321244 | | 1.943101 | |  | | Mean | | 1.940119 | | 2.40525 | 1.869543 | | 2.492278 | 1.943101 | |  |
| St Dev | | 0.038121 | | 0.33667 | | 0.02164 | 0.368758 | | 0.284324 | |  | | St Dev | | 0.035783 | | 0.40459 | 0.004038 | | 0.490213 | 0.284324 | |  |
| Minimum | | 1.69897 | | 2.176091 | | 1.69897 | 2.255273 | | 1.69897 | |  | | Minimum | | 1.724276 | | 2.30103 | 1.69897 | | 2.39794 | **1.69897** | |  |
| Maximum | | 1.986772 | | 2.39794 | | 1.894341 | 2.39794 | | 2.255273 | |  | | Maximum | | 2.049218 | | 2.69897 | 1.916433 | | 2.69897 | 2.255273 | |  |
| 25% Quantile | | 1.787016 | | 2.227422 | | 1.775114 | 2.282896 | | 1.787016 | |  | | 25% Quantile | | 1.849545 | | 2.352745 | 1.794732 | | 2.433121 | **1.787016** | |  |
| 75% Quantile | | 1.943471 | | 2.349485 | | 1.87872 | 2.384504 | | 2.065167 | |  | | 75% Quantile | | 2.007113 | | 2.519707 | 1.897241 | | 2.588046 | 2.065167 | |  |
| Kurtosis | | -2.33333 | | -2.33333 | | -2.33333 | -2.33333 | | -2.333333 | |  | | Kurtosis | | -2.33333 | | -2.33333 | -2.33333 | | -2.33333 | -2.33333 | |  |
| Skew | | -0.38079 | | 0.382563 | | -0.3849 | 0.3849 | | 0.2256 | |  | | Skew | | -0.3849 | | 0.384576 | -0.3849 | | 0.3849 | 0.2256 | |  |
| IQR | | 0.036409 | | 0.301906 | | 0.019685 | 0.349485 | | 0.278151 | |  | | IQR | | 0.03256 | | 0.390062 | 0.003687 | | 0.446439 | 0.278151 | |  |
|  | |  | |  | |  |  | |  | |  | |  | |  | |  |  | |  |  | |  |
| Symphysodon-SA Clade | | | | | |  |  | |  | |  | | Symphysodon-Heroini | | | | |  | |  |  | |  |
|  | | 2.50% | | 97.50% | | 0.25% | 99.75% | | Observed | |  | |  | | 2.50% | | 97.50% | 0.25% | | 99.75% | Observed | |  |
| Mean | | 1.892962 | | 2.276864 | | 1.818804 | 2.321244 | | 2.115733 | |  | | Mean | | 1.940119 | | 2.40525 | 1.869543 | | 2.492278 | 2.115733 | |  |
| St Dev | | 0.038121 | | 0.33667 | | 0.02164 | 0.368758 | | **0.02378** | |  | | St Dev | | 0.035783 | | 0.40459 | 0.004038 | | 0.490213 | **0.02378** | |  |
| Minimum | | 1.69897 | | 2.176091 | | 1.69897 | 2.255273 | | 2.089905 | |  | | Minimum | | 1.724276 | | 2.30103 | 1.69897 | | 2.39794 | 2.089905 | |  |
| Maximum | | 1.986772 | | 2.39794 | | 1.894341 | 2.39794 | | 2.136721 | |  | | Maximum | | 2.049218 | | 2.69897 | 1.916433 | | 2.69897 | 2.136721 | |  |
| 25% Quantile | | 1.787016 | | 2.227422 | | 1.775114 | 2.282896 | | 2.10524 | |  | | 25% Quantile | | 1.849545 | | 2.352745 | 1.794732 | | 2.433121 | 2.10524 | |  |
| 75% Quantile | | 1.943471 | | 2.349485 | | 1.87872 | 2.384504 | | 2.128647 | |  | | 75% Quantile | | 2.007113 | | 2.519707 | 1.897241 | | 2.588046 | 2.128647 | |  |
| Kurtosis | | -2.33333 | | -2.33333 | | -2.33333 | -2.33333 | | -2.333333 | |  | | Kurtosis | | -2.33333 | | -2.33333 | -2.33333 | | -2.33333 | -2.33333 | |  |
| Skew | | -0.38079 | | 0.382563 | | -0.3849 | 0.3849 | | -0.195127 | |  | | Skew | | -0.3849 | | 0.384576 | -0.3849 | | 0.3849 | -0.19513 | |  |
| IQR | | 0.036409 | | 0.301906 | | 0.019685 | 0.349485 | | **0.023408** | |  | | IQR | | 0.03256 | | 0.390062 | 0.003687 | | 0.446439 | **0.023408** | |  |
|  | |  | |  | |  |  | |  | |  | |  | |  | |  |  | |  |  | |  |
| Theraps-CA Clade | | | |  | |  |  | |  | |  | | Theraps-Heroini | | | |  |  | |  |  | |  |
|  | | 2.50% | | 97.50% | | 0.25% | 99.75% | | Observed | |  | |  | | 2.50% | | 97.50% | 0.25% | | 99.75% | Observed | |  |
| Mean | | 2.031225 | | 2.345705 | | 1.987378 | 2.387793 | | 2.330311 | |  | | Mean | | 2.02846 | | 2.340457 | 1.983509 | | 2.393769 | 2.330311 | |  |
| St Dev | | 0.101517 | | 0.308088 | | 0.068935 | 0.333168 | | 0.128952 | |  | | St Dev | | 0.09991 | | 0.303122 | 0.078166 | | 0.340854 | 0.128952 | |  |
| Minimum | | 1.724276 | | 2.146128 | | 1.724276 | 2.236775 | | 2.079181 | |  | | Minimum | | 1.69897 | | 2.136956 | 1.69897 | | 2.223999 | 2.079181 | |  |
| Maximum | | 2.266874 | | 2.69897 | | 2.146128 | 2.69897 | | 2.477121 | |  | | Maximum | | 2.245451 | | 2.69897 | 2.161035 | | 2.69897 | 2.477121 | |  |
| 25% Quantile | | 1.883652 | | 2.260904 | | 1.831872 | 2.363893 | | **2.289892** | |  | | 25% Quantile | | 1.883424 | | 2.261889 | 1.824474 | | 2.346938 | **2.289892** | |  |
| 75% Quantile | | 2.124911 | | 2.477121 | | 2.027024 | 2.55083 | | 2.39794 | |  | | 75% Quantile | | 2.112557 | | 2.477121 | 2.046594 | | 2.556853 | 2.39794 | |  |
| Kurtosis | | -2.06419 | | -0.30851 | | -2.15135 | 0.415512 | | -0.676317 | |  | | Kurtosis | | -2.05418 | | -0.26672 | -2.13411 | | 0.199449 | -0.67632 | |  |
| Skew | | -0.91012 | | 0.889704 | | -1.36752 | 1.162098 | | -0.805641 | |  | | Skew | | -0.95354 | | 0.912461 | -1.29558 | | 1.223328 | -0.80564 | |  |
| IQR | | 0.080714 | | 0.458053 | | 0.046716 | 0.538295 | | 0.108048 | |  | | IQR | | 0.088249 | | 0.449154 | 0.051154 | | 0.543017 | 0.108048 | |  |
|  | |  | |  | |  |  | |  | |  | |  | |  | |  |  | |  |  | |  |
| Thorichthys-CA Clade | | | | | |  |  | |  | |  | | Thorichthys-Heroini | | | |  |  | |  |  | |  |
|  | | 2.50% | | 97.50% | | 0.25% | 99.75% | | Observed | |  | |  | | 2.50% | | 97.50% | 0.25% | | 99.75% | Observed | |  |
| Mean | | 2.053328 | | 2.32579 | | 1.99612 | 2.370788 | | 2.176604 | |  | | Mean | | 2.041345 | | 2.319021 | 1.987705 | | 2.377717 | 2.176604 | |  |
| St Dev | | 0.113155 | | 0.302369 | | 0.089192 | 0.351671 | | **0.035492** | |  | | St Dev | | 0.1078 | | 0.305633 | 0.078219 | | 0.346207 | **0.035492** | |  |
| Minimum | | 1.724276 | | 2.113943 | | 1.724276 | 2.20412 | | 2.146128 | |  | | Minimum | | 1.69897 | | 2.120737 | 1.69897 | | 2.203976 | **2.146128** | |  |
| Maximum | | 2.278754 | | 2.69897 | | 2.190036 | 2.69897 | | **2.230449** | |  | | Maximum | | 2.269513 | | 2.69897 | 2.197191 | | 2.69897 | **2.230449** | |  |
| 25% Quantile | | 1.877899 | | 2.253088 | | 1.848412 | 2.295484 | | 2.146128 | |  | | 25% Quantile | | 1.877669 | | 2.25313 | 1.834507 | | 2.341295 | 2.146128 | |  |
| 75% Quantile | | 2.146108 | | 2.477121 | | 2.087012 | 2.552508 | | 2.189681 | |  | | 75% Quantile | | 2.115749 | | 2.442803 | 2.05007 | | 2.501143 | 2.189681 | |  |
| Kurtosis | | -1.97395 | | -0.19839 | | -2.14107 | 0.372635 | | -1.467491 | |  | | Kurtosis | | -1.97831 | | 0.020259 | -2.07746 | | 0.949426 | -1.46749 | |  |
| Skew | | -0.86061 | | 0.844329 | | -1.28999 | 1.192641 | | 0.625648 | |  | | Skew | | -0.87935 | | 1.0372 | -1.51525 | | 1.405416 | 0.625648 | |  |
| IQR | | 0.092196 | | 0.484397 | | 0.059478 | 0.558406 | | **0.043553** | |  | | IQR | | 0.095158 | | 0.457009 | 0.055637 | | 0.566928 | **0.043553** | |  |
|  | |  | |  | |  |  | |  | |  | |  | |  | |  |  | |  |  | |  |
| Uaru -SA Clade | | | |  | |  |  | |  | |  | | Uaru-Heroini | | | |  |  | |  |  | |  |
|  | | 2.50% | | 97.50% | | 0.25% | 99.75% | | Observed | |  | |  | | 2.50% | | 97.50% | 0.25% | | 99.75% | Observed | |  |
| Mean | | 1.836049 | | 2.326606 | | 1.775114 | 2.384504 | | **2.338347** | |  | | Mean | | 1.899405 | | 2.437531 | 1.792532 | | 2.548455 | 2.338347 | |  |
| St Dev | | 0.007583 | | 0.386562 | | 0 | 0.475245 | | 0.084278 | |  | | St Dev | | 0.004874 | | 0.476353 | 0 | | 0.598503 | 0.084278 | |  |
| Minimum | | 1.69897 | | 2.278754 | | 1.69897 | 2.371068 | | 2.278754 | |  | | Minimum | | 1.724276 | | 2.39794 | 1.69897 | | 2.414973 | 2.278754 | |  |
| Maximum | | 1.913814 | | 2.39794 | | 1.851258 | 2.39794 | | **2.39794** | |  | | Maximum | | 1.959041 | | 2.676694 | 1.854165 | | 2.69897 | 2.39794 | |  |
| 25% Quantile | | 1.767509 | | 2.301832 | | 1.737042 | 2.377786 | | **2.30855** | |  | | 25% Quantile | | 1.840977 | | 2.417735 | 1.747915 | | 2.473198 | 2.30855 | |  |
| 75% Quantile | | 1.869111 | | 2.353558 | | 1.813186 | 2.391222 | | **2.368143** | |  | | 75% Quantile | | 1.918815 | | 2.516791 | 1.832741 | | 2.623713 | 2.368143 | |  |
| Kurtosis | | -2.75 | | -2.75 | | -2.75 | 0 | | -2.75 | |  | | Kurtosis | | -2.75 | | -2.75 | -2.75 | | 0 | -2.75 | |  |
| Skew | | -8.5E-15 | | 1.02E-14 | | -2.1E-14 | 1.75E-14 | | 0 | |  | | Skew | | -8.7E-15 | | 8.64E-15 | -9.5E-14 | | 4.5E-14 | 0 | |  |
| IQR | | 0.005362 | | 0.273341 | | 0 | 0.336049 | | 0.059593 | |  | | IQR | | 0.003447 | | 0.336832 | 0 | | 0.423206 | 0.059593 | |  |
|  | |  | |  | |  |  | |  | |  | |  | |  | |  |  | |  |  | |  |
|  | |  | |  | |  |  | |  | |  | |  | |  | |  |  | |  |  | |  |
| Comparisons at higher taxonomic levels | | | | | | |  | |  | |  | |  | |  | |  |  | |  |  | |  |
|  | |  | |  | |  |  | |  | |  | |  | |  | |  |  | |  |  | |  |
| Geophagini - All Cichlids | | | | | |  |  | |  | |  | |  | |  | |  |  | |  |  | |  |
|  | | 2.50% | | 97.50% | | 0.25% | 99.75% | | Observed | |  | |  | |  | |  |  | |  |  | |  |
| 1 | | 2.052422 | | 2.107937 | | 2.041332 | 2.119929 | | **1.969968** | |  | |  | |  | |  |  | |  |  | |  |
| 2 | | 0.269915 | | 0.305892 | | 0.261802 | 0.312102 | | **0.314872** | |  | |  | |  | |  |  | |  |  | |  |
| 3 | | 1.361728 | | 1.50515 | | 1.361728 | 1.518514 | | **1.322219** | |  | |  | |  | |  |  | |  |  | |  |
| 4 | | 2.740363 | | 2.995635 | | 2.69897 | 2.995635 | | **2.494155** | |  | |  | |  | |  |  | |  |  | |  |
| 5 | | 1.846638 | | 1.920378 | | 1.818492 | 1.947559 | | **1.690196** | |  | |  | |  | |  |  | |  |  | |  |
| 6 | | 2.248575 | | 2.30103 | | 2.230449 | 2.341432 | | **2.236784** | |  | |  | |  | |  |  | |  |  | |  |
| 7 | | -0.6329 | | 0.076216 | | -0.76477 | 0.233699 | | **-1.252109** | |  | |  | |  | |  |  | |  |  | |  |
| 8 | | -0.23276 | | 0.17984 | | -0.31703 | 0.282059 | | -0.156872 | |  | |  | |  | |  |  | |  |  | |  |
| 9 | | 0.355096 | | 0.441742 | | 0.332768 | 0.464058 | | **0.546588** | |  | |  | |  | |  |  | |  |  | |  |
|  | |  | |  | |  |  | |  | |  | |  | |  | |  |  | |  |  | |  |
| CAS - Geophagini | | | |  | |  |  | |  | |  | |  | |  | |  |  | |  |  | |  |
|  | | 2.50% | | 97.50% | | 0.25% | 99.75% | | Observed | |  | |  | |  | |  |  | |  |  | |  |
| 1 | | 1.9491 | | 1.992467 | | 1.941217 | 1.999118 | | **1.943721** | |  | |  | |  | |  |  | |  |  | |  |
| 2 | | 0.305464 | | 0.323186 | | 0.30255 | 0.32676 | | **0.331088** | |  | |  | |  | |  |  | |  |  | |  |
| 3 | | 1.322219 | | 1.380211 | | 1.322219 | 1.39794 | | **1.322219** | |  | |  | |  | |  |  | |  |  | |  |
| 4 | | 2.477121 | | 2.494155 | | 2.477121 | 2.494155 | | **2.494155** | |  | |  | |  | |  |  | |  |  | |  |
| 5 | | 1.643453 | | 1.724276 | | 1.627062 | 1.730364 | | **1.623249** | |  | |  | |  | |  |  | |  |  | |  |
| 6 | | 2.216627 | | 2.255273 | | 2.197955 | 2.262451 | | **2.34259** | |  | |  | |  | |  |  | |  |  | |  |
| 7 | | -1.33049 | | -1.15684 | | -1.35918 | -1.11328 | | **-1.381065** | |  | |  | |  | |  |  | |  |  | |  |
| 8 | | -0.25582 | | -0.05915 | | -0.29602 | -0.01997 | | **0.005359** | |  | |  | |  | |  |  | |  |  | |  |
| 9 | | 0.515342 | | 0.593537 | | 0.507359 | 0.607576 | | **0.611009** | |  | |  | |  | |  |  | |  |  | |  |
|  | |  | |  | |  |  | |  | |  | |  | |  | |  |  | |  |  | |  |
| GGD - Geophagini | | | |  | |  |  | |  | |  | |  | |  | |  |  | |  |  | |  |
|  | | 2.50% | | 97.50% | | 0.25% | 99.75% | | Observed | |  | |  | |  | |  |  | |  |  | |  |
| 1 | | 1.891144 | | 2.052902 | | 1.862295 | 2.080005 | | **2.072677** | |  | |  | |  | |  |  | |  |  | |  |
| 2 | | 0.276039 | | 0.34836 | | 0.260795 | 0.356683 | | **0.21482** | |  | |  | |  | |  |  | |  |  | |  |
| 3 | | 1.322219 | | 1.518514 | | 1.322219 | 1.531479 | | **1.531479** | |  | |  | |  | |  |  | |  |  | |  |
| 4 | | 2.414973 | | 2.494155 | | 2.361578 | 2.494155 | | 2.447158 | |  | |  | |  | |  |  | |  |  | |  |
| 5 | | 1.593814 | | 1.855752 | | 1.566361 | 1.925564 | | **1.987885** | |  | |  | |  | |  |  | |  |  | |  |
| 6 | | 2.141372 | | 2.309073 | | 2.075028 | 2.348761 | | 2.237958 | |  | |  | |  | |  |  | |  |  | |  |
| 7 | | -1.53192 | | -0.8106 | | -1.62388 | -0.64385 | | **-0.104954** | |  | |  | |  | |  |  | |  |  | |  |
| 8 | | -0.56862 | | 0.230635 | | -0.69621 | 0.332199 | | **-0.620946** | |  | |  | |  | |  |  | |  |  | |  |
| 9 | | 0.400893 | | 0.663768 | | 0.347953 | 0.692137 | | **0.250072** | |  | |  | |  | |  |  | |  |  | |  |
|  | |  | |  | |  |  | |  | |  | |  | |  | |  |  | |  |  | |  |
| Cichlasomatini | | | |  | |  |  | |  | |  | |  | |  | |  |  | |  |  | |  |
|  | | 2.50% | | 97.50% | | 0.25% | 99.75% | | Observed | |  | |  | |  | |  |  | |  |  | |  |
| 1 | | 2.025686 | | 2.143467 | | 1.994244 | 2.169302 | | **1.976018** | |  | |  | |  | |  |  | |  |  | |  |
| 2 | | 0.246971 | | 0.328426 | | 0.234454 | 0.340124 | | **0.15273** | |  | |  | |  | |  |  | |  |  | |  |
| 3 | | 1.361728 | | 1.60206 | | 1.361728 | 1.658009 | | 1.556303 | |  | |  | |  | |  |  | |  |  | |  |
| 4 | | 2.481443 | | 2.995635 | | 2.457066 | 2.995635 | | **2.30103** | |  | |  | |  | |  |  | |  |  | |  |
| 5 | | 1.772582 | | 1.984538 | | 1.743255 | 2.008897 | | 1.869232 | |  | |  | |  | |  |  | |  |  | |  |
| 6 | | 2.20412 | | 2.366349 | | 2.176807 | 2.39794 | | **2.080976** | |  | |  | |  | |  |  | |  |  | |  |
| 7 | | -1.0104 | | 0.513669 | | -1.20023 | 1.180057 | | -0.206061 | |  | |  | |  | |  |  | |  |  | |  |
| 8 | | -0.47207 | | 0.432968 | | -0.61351 | 0.584645 | | -0.466712 | |  | |  | |  | |  |  | |  |  | |  |
| 9 | | 0.297527 | | 0.492789 | | 0.260455 | 0.547883 | | **0.211744** | |  | |  | |  | |  |  | |  |  | |  |
|  | |  | |  | |  |  | |  | |  | |  | |  | |  |  | |  |  | |  |
| Heroini - All Cichlids | | | | | |  |  | |  | |  | |  | |  | |  |  | |  |  | |  |
|  | | 2.50% | | 97.50% | | 0.25% | 99.75% | | Observed | |  | |  | |  | |  |  | |  |  | |  |
| 1 | | 2.047941 | | 2.115242 | | 2.029814 | 2.128026 | | **2.179034** | |  | |  | |  | |  |  | |  |  | |  |
| 2 | | 0.262497 | | 0.310083 | | 0.254718 | 0.320113 | | **0.20776** | |  | |  | |  | |  |  | |  |  | |  |
| 3 | | 1.361728 | | 1.518514 | | 1.361728 | 1.543953 | | **1.69897** | |  | |  | |  | |  |  | |  |  | |  |
| 4 | | 2.716003 | | 2.995635 | | 2.659916 | 2.995635 | | **2.69897** | |  | |  | |  | |  |  | |  |  | |  |
| 5 | | 1.819544 | | 1.933229 | | 1.78083 | 1.971326 | | **2.011778** | |  | |  | |  | |  |  | |  |  | |  |
| 6 | | 2.232317 | | 2.334935 | | 2.229483 | 2.353847 | | **2.347249** | |  | |  | |  | |  |  | |  |  | |  |
| 7 | | -0.6971 | | 0.211449 | | -0.82651 | 0.415223 | | -0.565788 | |  | |  | |  | |  |  | |  |  | |  |
| 8 | | -0.27978 | | 0.237226 | | -0.38972 | 0.345585 | | -0.014476 | |  | |  | |  | |  |  | |  |  | |  |
| 9 | | 0.344118 | | 0.454412 | | 0.324056 | 0.482588 | | **0.335471** | |  | |  | |  | |  |  | |  |  | |  |
|  | |  | |  | |  |  | |  | |  | |  | |  | |  |  | |  |  | |  |
| Heroini - SA Clade | | | |  | |  |  | |  | |  | |  | |  | |  |  | |  |  | |  |
|  | | 2.50% | | 97.50% | | 0.25% | 99.75% | | Observed | |  | |  | |  | |  |  | |  |  | |  |
| 1 | | 2.097759 | | 2.262207 | | 2.053737 | 2.296592 | | **2.092652** | |  | |  | |  | |  |  | |  |  | |  |
| 2 | | 0.160087 | | 0.258108 | | 0.143545 | 0.276624 | | 0.182481 | |  | |  | |  | |  |  | |  |  | |  |
| 3 | | 1.69897 | | 1.919078 | | 1.69897 | 1.984533 | | 1.69897 | |  | |  | |  | |  |  | |  |  | |  |
| 4 | | 2.39794 | | 2.69897 | | 2.371068 | 2.69897 | | 2.39794 | |  | |  | |  | |  |  | |  |  | |  |
| 5 | | 1.90309 | | 2.146128 | | 1.880814 | 2.187431 | | 1.973128 | |  | |  | |  | |  |  | |  |  | |  |
| 6 | | 2.20412 | | 2.39794 | | 2.149193 | 2.447158 | | 2.20412 | |  | |  | |  | |  |  | |  |  | |  |
| 7 | | -1.53892 | | 0.252431 | | -1.69089 | 1.123547 | | -0.816251 | |  | |  | |  | |  |  | |  |  | |  |
| 8 | | -0.64077 | | 0.695212 | | -0.91154 | 1.05357 | | -0.21848 | |  | |  | |  | |  |  | |  |  | |  |
| 9 | | 0.1648 | | 0.428212 | | 0.135763 | 0.477996 | | 0.230992 | |  | |  | |  | |  |  | |  |  | |  |
|  | |  | |  | |  |  | |  | |  | |  | |  | |  |  | |  |  | |  |
| Heroini - CA Clade | | | |  | |  |  | |  | |  | |  | |  | |  |  | |  |  | |  |
|  | | 2.50% | | 97.50% | | 0.25% | 99.75% | | Observed | |  | |  | |  | |  |  | |  |  | |  |
| 1 | | 2.166831 | | 2.19151 | | 2.160939 | 2.196138 | | **2.191668** | |  | |  | |  | |  |  | |  |  | |  |
| 2 | | 0.20009 | | 0.214015 | | 0.195574 | 0.216415 | | 0.209557 | |  | |  | |  | |  |  | |  |  | |  |
| 3 | | 1.69897 | | 1.724276 | | 1.69897 | 1.770676 | | 1.724276 | |  | |  | |  | |  |  | |  |  | |  |
| 4 | | 2.69897 | | 2.69897 | | 2.676694 | 2.69897 | | 2.69897 | |  | |  | |  | |  |  | |  |  | |  |
| 5 | | 2 | | 2.041393 | | 1.986772 | 2.051158 | | 2.041393 | |  | |  | |  | |  |  | |  |  | |  |
| 6 | | 2.30103 | | 2.371068 | | 2.30103 | 2.380211 | | **2.380211** | |  | |  | |  | |  |  | |  |  | |  |
| 7 | | -0.73158 | | -0.42107 | | -0.79277 | -0.35906 | | -0.627253 | |  | |  | |  | |  |  | |  |  | |  |
| 8 | | -0.10742 | | 0.110477 | | -0.15723 | 0.163694 | | -0.008269 | |  | |  | |  | |  |  | |  |  | |  |
| 9 | | 0.291046 | | 0.367374 | | 0.266103 | 0.374956 | | 0.338819 | |  | |  | |  | |  |  | |  |  | |  |
|  | |  | |  | |  |  | |  | |  | |  | |  | |  |  | |  |  | |  |
| Astronotini-All Cichlids | | | | | |  |  | |  | |  | |  | |  | |  |  | |  |  | |  |
|  | | 2.50% | | 97.50% | | 0.25% | 99.75% | | Observed | |  | |  | |  | |  |  | |  |  | |  |
| 1 | | 1.681047 | | 2.462204 | | 1.566112 | 2.701067 | | **2.520064** | |  | |  | |  | |  |  | |  |  | |  |
| 2 | | 0.008184 | | 0.626661 | | 0 | 0.773984 | | 0.197781 | |  | |  | |  | |  |  | |  |  | |  |
| 3 | | 1.447158 | | 2.392697 | | 1.361728 | 2.48783 | | 2.380211 | |  | |  | |  | |  |  | |  |  | |  |
| 4 | | 1.747992 | | 2.740363 | | 1.596535 | 2.995635 | | 2.659916 | |  | |  | |  | |  |  | |  |  | |  |
| 5 | | 1.59174 | | 2.406371 | | 1.502147 | 2.585189 | | **2.450137** | |  | |  | |  | |  |  | |  |  | |  |
| 6 | | 1.72094 | | 2.572464 | | 1.590971 | 2.848351 | | **2.58999** | |  | |  | |  | |  |  | |  |  | |  |
| 7 | | -2.75 | | -2.75 | | -2.75 | 0 | | -2.75 | |  | |  | |  | |  |  | |  |  | |  |
| 8 | | -7E-15 | | 7.05E-15 | | -5.4E-14 | 2.73E-14 | | 0 | |  | |  | |  | |  |  | |  |  | |  |
| 9 | | 0.005787 | | 0.443116 | | 0 | 0.54729 | | 0.139853 | |  | |  | |  | |  |  | |  |  | |  |
|  | |  | |  | |  |  | |  | |  | |  | |  | |  |  | |  |  | |  |
| Chaetobranchini-All Cichlids | | | | | |  |  | |  | |  | |  | |  | |  |  | |  |  | |  |
|  | | 2.50% | | 97.50% | | 0.25% | 99.75% | | Observed | |  | |  | |  | |  |  | |  |  | |  |
| 1 | | 1.826529 | | 2.344956 | | 1.71103 | 2.465322 | | 2.210577 | |  | |  | |  | |  |  | |  |  | |  |
| 2 | | 0.081867 | | 0.493762 | | 0.037836 | 0.578475 | | 0.152578 | |  | |  | |  | |  |  | |  |  | |  |
| 3 | | 1.39794 | | 2.146205 | | 1.361728 | 2.311343 | | 2.079181 | |  | |  | |  | |  |  | |  |  | |  |
| 4 | | 2.041393 | | 2.845404 | | 1.778151 | 2.995635 | | 2.361728 | |  | |  | |  | |  |  | |  |  | |  |
| 5 | | 1.599255 | | 2.260237 | | 1.534025 | 2.377457 | | 2.079181 | |  | |  | |  | |  |  | |  |  | |  |
| 6 | | 1.923826 | | 2.49288 | | 1.758547 | 2.701336 | | 2.332096 | |  | |  | |  | |  |  | |  |  | |  |
| 7 | | -2.38363 | | -1.70492 | | -2.42504 | -1.68878 | | **-2.412567** | |  | |  | |  | |  |  | |  |  | |  |
| 8 | | -0.70939 | | 0.666083 | | -0.74296 | 0.743033 | | 0.021653 | |  | |  | |  | |  |  | |  |  | |  |
| 9 | | 0.070448 | | 0.599477 | | 0.03354 | 0.845083 | | 0.252915 | |  | |  | |  | |  |  | |  |  | |  |
|  | |  | |  | |  |  | |  | |  | |  | |  | |  |  | |  |  | |  |
| Cichlini-All Cichlids | | | |  | |  |  | |  | |  | |  | |  | |  |  | |  |  | |  |
|  | | 2.50% | | 97.50% | | 0.25% | 99.75% | | Observed | |  | |  | |  | |  |  | |  |  | |  |
| 1 | | 1.935767 | | 2.213509 | | 1.895119 | 2.269503 | | **2.629601** | |  | |  | |  | |  |  | |  |  | |  |
| 2 | | 0.194976 | | 0.382765 | | 0.163322 | 0.424447 | | **0.175769** | |  | |  | |  | |  |  | |  |  | |  |
| 3 | | 1.380211 | | 1.845098 | | 1.361728 | 1.939454 | | **2.419956** | |  | |  | |  | |  |  | |  |  | |  |
| 4 | | 2.334403 | | 2.995635 | | 2.231716 | 2.995635 | | 2.995635 | |  | |  | |  | |  |  | |  |  | |  |
| 5 | | 1.678967 | | 2.091493 | | 1.604728 | 2.163423 | | **2.521755** | |  | |  | |  | |  |  | |  |  | |  |
| 6 | | 2.098185 | | 2.409797 | | 2.023683 | 2.485587 | | **2.69537** | |  | |  | |  | |  |  | |  |  | |  |
| 7 | | -1.58869 | | 0.667174 | | -1.71971 | 1.985798 | | -0.810136 | |  | |  | |  | |  |  | |  |  | |  |
| 8 | | -0.86604 | | 0.842708 | | -1.24449 | 1.410927 | | 0.71283 | |  | |  | |  | |  |  | |  |  | |  |
| 9 | | 0.183234 | | 0.586837 | | 0.139727 | 0.67688 | | **0.173614** | |  | |  | |  | |  |  | |  |  | |  |
|  | |  | |  | |  |  | |  | |  | |  | |  | |  |  | |  |  | |  |
| Retroculini-All Cichlids | | | | | |  |  | |  | |  | |  | |  | |  |  | |  |  | |  |
|  | | 2.50% | | 97.50% | | 0.25% | 99.75% | | Observed | |  | |  | |  | |  |  | |  |  | |  |
| 1 | | 1.74248 | | 2.376443 | | 1.599928 | 2.523274 | | 2.248204 | |  | |  | |  | |  |  | |  |  | |  |
| 2 | | 0.050775 | | 0.564068 | | 0.015183 | 0.666273 | | 0.079121 | |  | |  | |  | |  |  | |  |  | |  |
| 3 | | 1.431364 | | 2.230513 | | 1.361728 | 2.30103 | | 2.158362 | |  | |  | |  | |  |  | |  |  | |  |
| 4 | | 1.913546 | | 2.845098 | | 1.69897 | 2.995635 | | 2.307496 | |  | |  | |  | |  |  | |  |  | |  |
| 5 | | 1.596999 | | 2.288887 | | 1.488307 | 2.415564 | | 2.218558 | |  | |  | |  | |  |  | |  |  | |  |
| 6 | | 1.829482 | | 2.541635 | | 1.653603 | 2.698754 | | 2.293125 | |  | |  | |  | |  |  | |  |  | |  |
| 7 | | -2.33333 | | -2.33333 | | -2.33333 | -2.33333 | | **-2.33333** | |  | |  | |  | |  |  | |  |  | |  |
| 8 | | -0.38436 | | 0.383413 | | -0.3849 | 0.3849 | | -0.328549 | |  | |  | |  | |  |  | |  |  | |  |
| 9 | | 0.048455 | | 0.525662 | | 0.014383 | 0.652233 | | 0.074567 | |  | |  | |  | |  |  | |  |  | |  |
