# Supplementary material for: Body Size Diversity and Frequency Distributions of Neotropical Cichlid Fishes (Cichliformes: Cichlidae: Cichlinae)
Source: PLoS One. 2014 Sep 2;9(9):e106336. doi: 10.1371/journal.pone.0106336 (PMC4152270; doi:10.1371/journal.pone.0106336)
Supplement: Appendix S1 — Effects of total length data on body size distributions. Description of the effects of the inclusion and exclusion of total length data on summary statistics. (DOCX) [file pone.0106336.s006.docx]

**Appendix S1: Effects of total length data on body size distributions.**

The majority of total length data used were concentrated in the tribe Heroini. In total 176 species were used in the analysis, and 142 when total length data were removed from the analysis. When TL data were removed from the analysis, there were few changes in the summary statistics describing the distribution of Heroini or during the bootstrapping simulation comparing the distribution to all cichlids. The only notable difference was that the 75% quantile of Heroini was no longer significantly larger than expected from random data, however still was trending towards this direction.

From the South American (SA) Clade, only one species was recorded in total length (*Hoplarchus psittacus)*. The removal of this species did not result in any significant changes to the summary statistics describing the distribution of the SA Clade, with the exception of the maximum body size becoming significantly lower. This is due to the fact that *H. psittacus* was the largest species in this clade, and happens to be largely different in body size compared to other species.

From the Central American (CA) Clade, total length data were found in a number of clades. However, the removal of species represented by total length is more likely to cause biased results rather than incorporating data in the analyses due to the taxonomic bias of TL representation. By taking out TL data, the distributions of 12 genera would be affected (*Tomocichla* – 33.3% loss of species, *Amphilophus* – 15% loss, *Archocentrus* – 100% loss, *Australoheros* – 4% loss, *Cryptoheros* – 22% loss, *Hypsophrys* – 50% loss, *Parachromis* – 60% loss, *Astatheros* – 11% loss, *Herichthys* – 33% loss, *Paraneetroplus* – 43% loss, *Theraps* – 29% loss, *Thorichthys* – 13% loss) including 3 genera with greater than 50% species loss. This data is typically restricted to most or all species of a few clades ( noted above), which are mostly large-bodied cichlids at the upper range of the body size distribution. Removing these species and clades would cause a larger difference in the results than the slight differences between TL and SL obtained when data are log-transformed. The inclusion, however, of species represented by total length allowed us to incorporate all species with body size data in order to capture the extent of body size diversity in cichlids. Inclusion of these species had minor quantitative changes in summary statistics of larger clades (Table S3), but did not affect the overall patterns of body size that we observed.
